# Molecular metal nanoparticles with pseudo-plasma absorption band

**DOI:** 10.1093/nsr/nwag001

**Published:** 2026-01-10

**Authors:** Liang Fang, Rong Liu, Zongbing He, Guowei Guan, Lingwen Liao, Runguo Wang, Wanmiao Gu, Chengming Wang, Jin Li, Haiteng Deng, Shengli Zhuang, Zhou Lu, Zhikun Wu

**Affiliations:** Key Laboratory of Materials Physics, Anhui Key Laboratory of Nanomaterials and Nanotechnology, CAS Center for Excellence in Nanoscience, Institute of Solid State Physics, HFIPS, Chinese Academy of Sciences, Hefei 230031, China; Institute of Physical Science and Information Technology, Anhui University, Hefei 230601, China; Key Laboratory of Precision and Intelligent Chemistry, University of Science and Technology of China, Hefei 230026, China; Anhui Province Key Laboratory for Control and Applications of Optoelectronic Information Materials, School of Physics and Electronic Information, Key Laboratory of Functional Molecular Solids, Ministry of Education, Anhui Normal University, Wuhu 241002, China; Key Laboratory of Materials Physics, Anhui Key Laboratory of Nanomaterials and Nanotechnology, CAS Center for Excellence in Nanoscience, Institute of Solid State Physics, HFIPS, Chinese Academy of Sciences, Hefei 230031, China; Institute of Physical Science and Information Technology, Anhui University, Hefei 230601, China; Key Laboratory of Precision and Intelligent Chemistry, University of Science and Technology of China, Hefei 230026, China; Key Laboratory of Materials Physics, Anhui Key Laboratory of Nanomaterials and Nanotechnology, CAS Center for Excellence in Nanoscience, Institute of Solid State Physics, HFIPS, Chinese Academy of Sciences, Hefei 230031, China; Institute of Physical Science and Information Technology, Anhui University, Hefei 230601, China; Key Laboratory of Precision and Intelligent Chemistry, University of Science and Technology of China, Hefei 230026, China; Key Laboratory of Materials Physics, Anhui Key Laboratory of Nanomaterials and Nanotechnology, CAS Center for Excellence in Nanoscience, Institute of Solid State Physics, HFIPS, Chinese Academy of Sciences, Hefei 230031, China; Institute of Physical Science and Information Technology, Anhui University, Hefei 230601, China; Key Laboratory of Precision and Intelligent Chemistry, University of Science and Technology of China, Hefei 230026, China; Key Laboratory of Materials Physics, Anhui Key Laboratory of Nanomaterials and Nanotechnology, CAS Center for Excellence in Nanoscience, Institute of Solid State Physics, HFIPS, Chinese Academy of Sciences, Hefei 230031, China; Institute of Physical Science and Information Technology, Anhui University, Hefei 230601, China; Key Laboratory of Precision and Intelligent Chemistry, University of Science and Technology of China, Hefei 230026, China; Key Laboratory of Materials Physics, Anhui Key Laboratory of Nanomaterials and Nanotechnology, CAS Center for Excellence in Nanoscience, Institute of Solid State Physics, HFIPS, Chinese Academy of Sciences, Hefei 230031, China; Institute of Physical Science and Information Technology, Anhui University, Hefei 230601, China; Key Laboratory of Precision and Intelligent Chemistry, University of Science and Technology of China, Hefei 230026, China; Instruments Center for Physical Science, University of Science and Technology of China, Hefei 230026, China; Tsinghua University-Peking University Joint Center for Life Sciences, School of Life Sciences, Tsinghua University, Beijing 100084, China; MOE Key Laboratory of Bioinformatics, School of Life Sciences, Tsinghua University, Beijing 100084, China; Key Laboratory of Materials Physics, Anhui Key Laboratory of Nanomaterials and Nanotechnology, CAS Center for Excellence in Nanoscience, Institute of Solid State Physics, HFIPS, Chinese Academy of Sciences, Hefei 230031, China; Institute of Physical Science and Information Technology, Anhui University, Hefei 230601, China; Key Laboratory of Precision and Intelligent Chemistry, University of Science and Technology of China, Hefei 230026, China; Anhui Province Key Laboratory for Control and Applications of Optoelectronic Information Materials, School of Physics and Electronic Information, Key Laboratory of Functional Molecular Solids, Ministry of Education, Anhui Normal University, Wuhu 241002, China; Key Laboratory of Materials Physics, Anhui Key Laboratory of Nanomaterials and Nanotechnology, CAS Center for Excellence in Nanoscience, Institute of Solid State Physics, HFIPS, Chinese Academy of Sciences, Hefei 230031, China; Institute of Physical Science and Information Technology, Anhui University, Hefei 230601, China; Key Laboratory of Precision and Intelligent Chemistry, University of Science and Technology of China, Hefei 230026, China

**Keywords:** critical size, anti-galvanic reduction, molecular state, crystallization-induced enhancement, size dependence, ultrafast dynamics

## Abstract

Metal nanoparticles include molecular nanoclusters and metallic nanocrystals. Investigating the critical transition sizes from nanoclusters to nanocrystals is appealing. However, achieving precise size control near the critical size region remains challenging, especially for not-so-noble metal nanoparticles (Ag, Cu etc.). Herein, we introduced an active metal anti-galvanic doping strategy to resolve both stability and multi-dispersity issues and demonstrated the gram-scale synthesis (2.40 g of crystals, more than 200 times the existing crystal output record for over 100-metal-atom nanoparticles) of a 1796-atom Ag–Zn nanoparticle. Furthermore, we successfully de-alloyed the Ag–Zn nanoparticles with the remaining structure essentially unchanged via a ligand-exchange method, obtaining 1.03 g of mono-Ag nanoparticle crystals in a one-pot reaction. Such a surgery-like de-alloying was not previously reported. Both of the as-obtained nanoparticles exhibit penta-twinned face-centered cubic (*fcc*) structures with well-defined shape–number arrangements and display plasmon-like absorptions yet exist in molecular states, as evidenced by ultrafast dynamics measurements. Furthermore, crystallization-induced photothermal enhancement and size-dependent absorbance were observed.

## Teaser

Two molecular giant nanoclusters with pseudo-plasma absorption bands and perfect shape-number structures were obtained in gram scale crystal, and the crystallization-induced photothermal enhancement and size-dependent absorbance were observed.

## INTRODUCTION

The transition from the molecular to the continuum state is a core issue in nanoscience and nanotechnology [1–6]. With advances in precise nanochemistry, the pursuit of atomically precise transition sizes has continued, and great progress has been achieved despite persistent research gaps and ongoing debates owing to experimental and theoretical challenges [7–13]. For example, many groups such as Goodson’s [14], Jin’s [15–18], Tsukuda’s [19,20], Pettersson’s [21], Knappenberger’s [22], Dass’s [23,24], Wang’s [25,26] and Wu’s [27,28] have proposed various transition size ranges for gold nanoparticles (including molecular nanoclusters and continuum-state nanocrystals; note that cation clusters are not considered here). To date, most discussions on critical size have focused on Au nanoparticles owing to their exceptional stability [29–32]. Atomically precise, considerably large Cu nanoclusters (Cu*_n_, n* > 100) have rarely been reported, making it difficult to discuss the critical size of Cu nanoparticles. Although substantially large Ag nanoparticles (Ag*_n_, n* > 100) have been reported [33, 34], critical-size investigations of Ag nanoparticles have been severely hindered by their instability and the difficulty of synthesizing atomically precise [5], near-critical-sized monodisperse Ag nanoparticles in sufficient quantities. The high-quality synthesis of stable Ag nanoparticles is critical not only for resolving this fundamental issue but also for practical applications (e.g. photoelectric devices), which stimulated our interest.

Alloying is an efficient strategy to enhance the stability of metal nanoparticles [5,35–40]. Moreover, alloying can strengthen other properties such as luminescence and waveguiding [9,41–43]. Unfortunately, only a few Ag-based alloy nanoparticles with a precise metal atom number larger than 100 have been reported to date (Table S1). Owing to the similarity between Au (or Cu) and Ag atoms, it is difficult to control their nucleation process, and the resulting Ag–Au(Cu) nanoparticles are generally multi-disperse [38,44,45]. Thus, to obtain monodisperse alloy nanoparticles, doping Ag nanoparticles with analog metals (e.g. Au or Cu) is best avoided. Alternatively, the incorporation of active and abundant metals such as Zn and iron is particularly attractive for both fundamental studies and practical applications. However, reducing Zn or iron ions to their metallic forms is challenging under the common conditions adopted for coinage metal nanocluster synthesis. Fortunately, the recently introduced ‘anti-galvanic reaction’ provides an unparalleled method for synthesizing Ag nanoparticles doped with markedly active metals [46–50].

## RESULTS

### Gram-scale synthesis and characterization

**Figure 1.**
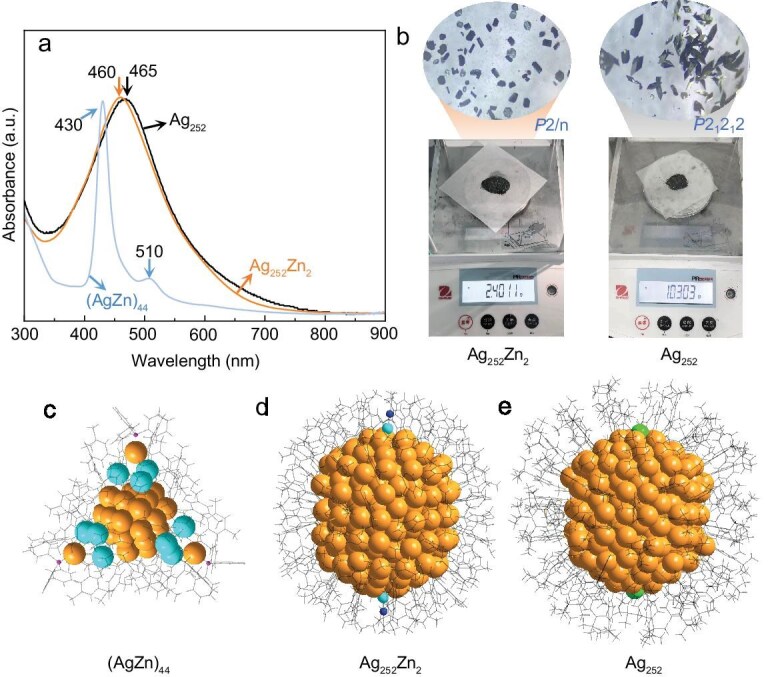
Optical properties, gram-scale crystals and X-ray single-crystal structures of the as-synthesized NPs. (a) UV-Vis-NIR absorption spectra and (b) gram-scale crystals of **Ag_252_Zn_2_** and **Ag_252_**. Total structures of (c) **(AgZn)_44_**, (d) **Ag_252_Zn_2_** and (e) **Ag_252_**. For clarity, H atoms are omitted. Surface-protecting motifs are shown in ball-and-stick form, and carbon tails are in wireframe form. Color labels: gold, Ag; cyan, Zn or Cl; blue, N; green, Cl.

Herein, we successfully obtained a Ag–Zn bimetal nanoparticle, Ag_252_Zn_2_(3,5-DMBT)_85_(MeCN)_2_ (hereafter referred to as **Ag_252_Zn_2_**), by exploiting the strong reducibility and affinity of nucleating Ag nanoparticles. For experimental details, see the Supplementary  data. During the reaction, an intermediate product **(AgZn)_44_** can be isolated and crystallized (Figs S1 and S2). Notably, Zn ions cannot be reduced to Zn nanoclusters under similar conditions without the presence of Ag ions, demonstrating the occurrence of the anti-galvanic reaction during the alloying, consistently reported in previous works [48–50]. **(AgZn)_44_** itself can also be transformed to **Ag_252_Zn_2_** under similar conditions; however, the reaction mixture without isolating **(AgZn)_44_** results in a high conversion rate to **Ag_252_Zn_2_**, indicating additional size-focusing processes during the synthesis of **Ag_252_Zn_2_** [29]. Without the coexistence of Zn^2+^ in the reaction mixture, high-quality crystals of **Ag_252_Zn_2_** are difficult to obtain, indicating that doped Zn plays an important role in the formation and crystallization of **Ag_252_Zn_2_**. Interestingly, the doped Zn can be precisely and completely removed through a ligand-exchange process. Briefly, **Ag_252_Zn_2_** crystals were dissolved in a mixture of toluene and dichloromethane, followed by the addition of excess 4-tert-butylbenzenethiol (TBBT) and reaction at 80°C for over 12 h, with subsequent purification similar to that used for **Ag_252_Zn_2_** (see Materials and methods in the Supplementary data for details). Notably, the synthesis can be scaled to obtain 2.40 g of **Ag_252_Zn_2_** crystals (52.8% yield) and 1.03 g of **Ag_252_** crystals (21.3% yield) for single-crystal X-ray diffraction (SCXRD) analysis (Fig. 1b and c; larger-scale reactions were not attempted). Gram-scale production of high-quality single crystals suitable for SCXRD has not previously been reported; the highest output thus far is 11.54 mg (in 10% yield) reported by the Sun group in the crystallization of Ag_102_ (Table S1) for complex systems (>100 metal atoms, not limited to Ag) [51]. Large scale, high-quality syntheses provide abundant pure materials for subsequent studies and applications, as partially shown below.

To determine the molecular formulas and exact structures of the three nanoparticles, electrospray ionization mass spectrometry (ESI-MS) and SCXRD analyses were preferentially conducted. In the ESI-MS characterization of **(AgZn)_44_**, cesium acetate (CsOAc) was added to assist the ionization. As shown in Fig. S3, the dominant species is assigned to [Ag_32_Zn_12_(TPP)_4-_*_x_*(3,5-DMBT)_36_ + 3Cs]^3+^ (0 ≤ *x* ≤ 4). Other peaks with identical spacing fit perfectly with the molecular formula [Ag_32-_*_y_*Zn_12+_*_y_*(TPP)_4-_*_x_*(3,5-DMBT)_36_ + 3Cs]^3+^ (*y* = 0 and 1; Figs S4 and S5). The charge numbers of the cationic adducts match the numbers of Cs^+^, indicating that the analyzed nanoparticle is charge-neutral. Thus, the molecular formula was concluded to be Ag_32_Zn_12_(TPP)_3_(3,5-DMBT)_36_, as confirmed via SCXRD along with supporting thermal gravimetric analysis (TGA) and X-ray photoelectron spectroscopy (XPS) (Figs S6–S8). Mass spectrometry is a strong tool to identify the monodispersity and composition of metal nanoparticles; however, considerably large Ag nanoparticles are rarely characterized precisely via mass spectrometry. Fortunately, the composition of **Ag_252_** was determined using ESI-MS in a toluene/dichloromethane solution. As shown in Fig. S9, a dominant peak was observed at a mass/charge ratio (*m*/*z*) of 10284.54, which was readily attributed to [Ag_252_(3,5-DMBT)_4_(TBBT)_81_]^4+^ (calculated *m*/*z* = 10284.42; deviation = 0.12). Interestingly, when CsOAc was added to the **Ag_252_** analyte before ESI-MS characterization, two major peaks at 10284.45 and 8227.96 *m*/*z* appeared (Fig. S10), corresponding to [Ag_252_Cl_2_(3,5-DMBT)_4_(TBBT)_81_]^4+^ and [Ag_252_Cl_2_(3,5-DMBT)_4_(TBBT)_81_ + H]^5+^, respectively, with no Cs^+^ adsorption detected. This indicates that while CsOAc can aid in the ionization of **Ag_252_**, it exhibits weak affinity toward **Ag_252_**. The presence of the counterion NO_3_^−^ (*v*_N-O_ = 1380 cm^−1^) was verified using infrared (IR) spectroscopy (Fig. S11), supporting the assignment of **Ag_252_** as [Ag_252_Cl_2_(3,5-DMBT)_4_(TBBT)_81_]^4+^(NO_3_^−^)_4_, further validated via SCXRD, TGA and XPS (Figs S6–S8). Unfortunately, the molecular composition of **Ag_252_Zn_2_** could not be determined via ESI-MS despite multiple attempts, including the CsOAc-assisted ionization. However, SCXRD combined with TGA and XPS analyses confirmed its formula as Ag_252_Zn_2_(3,5-DMBT)_85_(MeCN)_2_ (Figs S6–S8). Acquiring high-quality SCXRD data for giant nanoclusters is challenging (Table S1), but both **Ag_252_Zn_2_** and **Ag_252_** crystals yielded data of sufficiently high quality, displaying low residual factor *R*_1_ values (5.7% for **Ag_252_Zn_2_** and 3.6% for **Ag_252_**), likely owing to their high purity and yield. The spatial dimensions of the three nanoparticles determined via SCXRD were further supported using high-resolution transmission electron microscopy (HR-TEM), as shown in Figs S12–S14.

### Single-crystal structure

**Figure 2.**
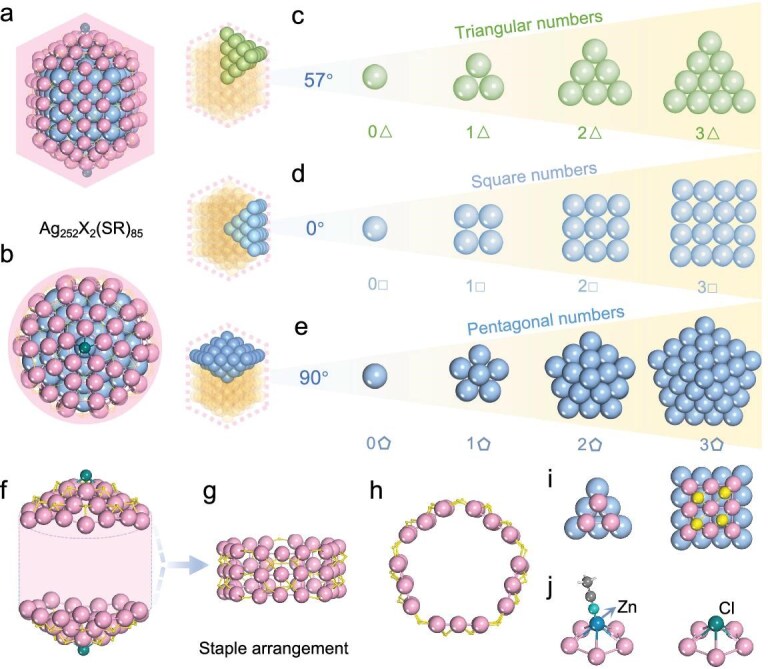
Single-crystal structures of **Ag_252_X_2_(SR)_85_** (X = Zn(MeCN) or Cl; SR = 3,5-DMBT or TBBT). (a) Side and (b) top views of **Ag_252_X_2_(SR)_85_** structure; compositions of Ag_147_ from inside to outside viewed along (c) 57°, (d) 0° and (e) 90°. The outer Ag_105_X_2_(SR)_85_ staple structures comprise (f) two apical units and (g and h) one side-ring unit. (i) Triangular and square number gradients in the arrangement of Ag and S atoms within the staple structure. (j) Connections of Zn(MeCN) and Cl atoms at the top-end positions of **Ag_252_Zn_2_** and **Ag_252_**, respectively. Color labels: light blue, light gold and pink, Ag; dark cyan, Zn; yellow, S; light cyan, N; dark green, Cl.

SCXRD reveals that **(AgZn)_44_** consists of an Ag_28_ kernel containing an Ag_4_ tetrahedron, capped by an exterior shell comprising four AgZn_3_(TPP)(3,5-DMBT)_9_ staples, as shown in Figs S15 and S16. The Ag_28_ kernel structure of **(AgZn)_44_** is identical to that of the reported Ag_28_Cu_12_ [52], also reminiscent of the truncated tetrahedral Au_28_ kernel in the structure of Au_36_(TBBT)_24_ [53], with main differences residing in the compositions of the peripheral protective structures (Fig. S17). Owing to its high reactivity, Zn is usually difficult to incorporate into nanoclusters, and our study represents the first successful case of Zn doping into Ag nanoclusters. The doping of Zn notably strengthens the stability of Ag nanoparticles, enabling purification via preparative thin-layer chromatography (PTLC)—a process previously unreported for Ag-based nanoclusters, unlike their Au-based counterparts. **Ag_252_Zn_2_** and **Ag_252_** nanoparticles share similar structures, with the main differences being their outer shell staples and space groups (Figs S18 and S19). A detailed structural dissection of **Ag_252_Zn_2_** or **Ag_252_** is illustrated in Fig. 2. Their overall architectures can be regarded as simplified Ag_147_@Ag_105_X_2_(SR)_85_ kernel-shell structure [X = Zn(MeCN) or Cl] (Fig. 2a), both sharing a *C*_5_ axis passing through two X units at opposite poles, as shown in Fig. 2b. The multiangle atomic projection arrangements from the inside to the outside of the Ag_147_ kernels in both nanoparticles align perfectly with the characteristics of *shape number*—a concept proposed by the Greek mathematician Eudoxos, who believed that numbers are the origin of all things and that a law combining numbers and shapes reveals the potential mysteries of nature [54]. The atomic layers exhibit distinct planar polygonal patterns when viewed from different angles (57°, 0° and 90°; note that the angle is defined between the polygon pattern plane and the equatorial plane of the cluster) in an inward-to-outward cross-sectional fashion (Fig. 2c–e). For example, when viewed at a 57° angle, the metal atoms appear to progress outward in a triangular number pattern (Fig. 2c), with each layer following a segmented gradient of triangular numbers (0 × 0, 1 × 1, 2 × 2 and 3 × 3). Similar patterns are observed in Fig. 2d and e, where the atoms conform to square and pentagonal number gradients, respectively. In both nanoparticles, the largest shape number observed is 3 × 3, which may enlarge (e.g. 4 × 4 and 5 × 5) with increasing nanoparticle size—a hypothesis to be verified in future studies. Notably, such a shape–number phenomenon has not previously mentioned in metal nanoparticles, but it does exist in some nanoparticles such as Ag_155_ [55], Ag_307_ [33] and Au_130_ (with two-shell Ino decahedron) [56], indicating its universality. For nanocrystals without precise compositions and structures, there are no shape–number phenomenon reports despite them exhibiting distinct morphologies or phases [57,58]. Interestingly, increases in these gradients do not profoundly affect Ag–Ag bond distances. The Ag–Ag average bond length in the 1 × 1 graded layer is 2.88 Å, closely matching those in the 2 × 2 (2.88 Å) and 3 × 3 (2.89 Å) graded layers (Figs S20 and S21, Tables S2 and S3). The outer staple structures of **Ag_252_Zn_2_** and **Ag_252_** include two apical units and one side-ring unit (Fig. 2f and g). As displayed in Fig. S22, each apical staple structure features five *μ*_4_-S atoms in the inner circle and five *μ*_4_-S and four *μ*_3_-S in the outer circle. A *C*_5_ symmetry is observed in the side view of the staple (Fig. 2g and h), with each minimum symmetrical structural unit containing nine Ag atoms coordinated by four S atoms (Fig. S23). Adjacent structural units are connected via three *μ*_2_-S atoms, while the side and top/bottom staples are linked through a total of 20 *μ*_2_-S atoms. Interestingly, disregarding atom type differences, the Ag and S atoms in the staple structure, as well as the kernel Ag atoms, exhibit triangular and square number gradients (Fig. 2i). These gradients from the kernel to the periphery do not result in profound changes in Ag–Ag or Ag–S bond distances (Fig. S24). In the staple structures of **Ag_252_Zn_2_** and **Ag_252_**, the average Ag–Ag bond length is 3.11 Å, and the average Ag–S bond length is 2.60 Å for both (Tables S4 and S5). The primary differences between **Ag_252_Zn_2_** and **Ag_252_** lie in their outer ligands (3,5-DMBT vs. TBBT) and the terminal atoms at the bottom and top: **Ag_252_Zn_2_** terminates with Zn(MeCN) at both ends, while **Ag_252_** terminates with Cl atoms (Fig. 2j, Figs S6 and S25). These structural (compositional) differences lead to notable deviations in their stability: **Ag_252_** starts to decompose at 246°C, while **Ag_252_Zn_2_** decomposes at a relatively lower temperature of 197°C (Fig. S7). The dilute solution of **Ag_252_** remains stable for over 72 h at 100°C, while prominent changes are observed in the dilute solution of **Ag_252_Zn_2_** under similar conditions (Fig. S26) after 36 h of heating. Notably, at considerably lower temperature (e.g. 80°C), both dilute solutions remain stable for over 72 h (Fig. S27). Compared with **Ag_252_Zn_2_** and **Ag_252_**, the relatively small **(AgZn)_44_** nanoclusters show obviously lower stability, and they can decompose at 106° even in solid state (Fig. S7). It is known that metal nanoparticle stability is a complex issue, involving multiple factors such as metal type, ligand type, structure (spatial and electrical) and size, which corporately rather than alone determine the nanoparticle stability. Considering a single factor alone, generally speaking, the nanoparticle stability increases with the size (ligand protective capability) increase, and tends to follow the order of Au > Ag > Cu; the close-packed (shell-closure) structure is more stable than the incompact (shell-open) structure etc.

### Ultrafast electron dynamics and EPR measurement

**Figure 3.**
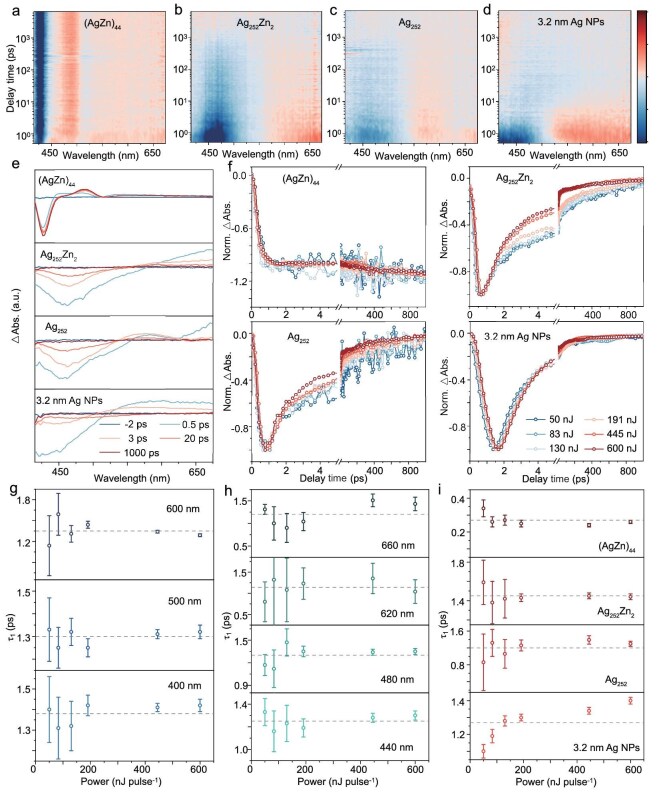
TA data maps of (a) **(AgZn)_44_**, (b) **Ag_252_Zn_2_**, (c) **Ag_252_** and (d) Ag NPs with pump at 400 nm in toluene; orange represents ESA, and blue represents GSB. (e) Absorption difference spectra of **(AgZn)_44_, Ag_252_Zn_2_, Ag_252_** and Ag NPs after pulsed laser excitation at several representative time delays. (f) Normalized decay kinetics as a function of laser fluence with 400 nm pump. Electron–phonon relaxation lifetimes (τ_1_) of **Ag_252_Zn_2_** under (g) different pump wavelengths and (h) different probe wavelengths at 400 nm excitation. (i) Extracted τ_1_ of **(AgZn)_44_, Ag_252_Zn_2_, Ag_252_** and Ag NPs as a function of pump fluence.

Although the two nanoclusters show typical single absorption peaks centered at 460 nm (**Ag_252_Zn_2_**) and 465 nm (**Ag_252_**) in a wider wavelength range from 200 to 1600 nm (Fig. 1a and Fig. S28), they lack the full *fcc* kernel structure common to Ag nanocrystals, raising questions about their plasmonic properties. To testify this, ultrafast transient absorption (TA) measurements were performed to determine the metallic or molecular properties of **Ag_252_Zn_2_** and **Ag_252_**. For comparison, TA dynamics of ∼3.2 nm Ag nanoparticles (Fig. S29) and molecular **(AgZn)_44_** were also investigated, as shown in Fig. 3a–e. **(AgZn)_44_** displays a positive excited state absorption (ESA) feature centered at 490 nm and two ground-state bleaching (GSB) bands centered at 430 and 510 nm. By contrast, **Ag_252_Zn_2_** and **Ag_252_** exhibit a GSB peak centered at ∼460 nm, a shoulder peak centered at ∼525 nm and an ESA peak located at >550 nm. Ag nanocrystals exhibit a GSB peak centered at ∼450 nm and a broad ESA peak distributed at >525 nm. TA spectra of **Ag_252_Zn_2_**, with excitation at 600 nm, also exhibit a shoulder peak at around 525 nm. No emission signals were detected around 525 nm (Fig. S30). Moreover, both **Ag_252_Zn_2_** and **Ag_252_** display significant optical absorption around 525 nm. Therefore, the shoulder peak centered at ∼525 nm can still be attributed to the GSB. TA measurements at different pump fluences were also tested (Figs S31–S34). Kinetics at the GSB peak were extracted from the femtosecond TA (fs-TA) spectra acquired after excitation at 400 nm (Fig. 3f), and fitting of four samples yielded corresponding time constants (Figs S35–S39 and Table S6). The typical molecular-like **(AgZn)_44_** was fitted using a triexponential function, including a subpicosecond internal conversion (IC) or intramolecular vibrational relaxation, several-hundred picosecond intersystem crossing, and a triplet relaxing process of >7.5 ns. For **Ag_252_Zn_2_** and **Ag_252_**, a triexponential function was also adopted to fit the kinetic traces, where τ_1_ denotes the IC time constant from high to low excited states, and τ_2_ and τ_3_ denote slower electronic relaxation from the lowest excited state to the ground state and the hot ground state relaxation time constants, respectively. By contrast, the kinetic curves of Ag nanoparticles (NPs) were fitted with a double-exponential function, which included a 1–2 ps electron–phonon coupling process and a several-hundred picosecond energy dissipation process. The τ_1_ versus power is shown in Fig. 3g, revealing no prominent power-dependence for **(AgZn)_44_, Ag_252_Zn_2_** and **Ag_252_**. Using **Ag_252_Zn_2_** as an example, additional excitation wavelengths (Figs S40 and S41) and various pump (Fig. 3g, Figs S39–S42 and Table S7) and probe (Fig. 3h, Fig. S43 and Table S8) wavelengths under 400 nm excitation were employed to probe its electron dynamics. In all these cases, results show that the electron dynamics of **Ag_252_Zn_2_** are power-independent. On the contrary, ∼3.2 nm Ag nanocrystals exhibited a power-dependent electron–phonon coupling process (Fig. 3i). Together, these findings demonstrate that ∼3.2 nm Ag nanocrystals are metallic, while **Ag_252_Zn_2_** and **Ag_252_** remain non-metallic. Low-temperature electron paramagnetic resonance (EPR) measurements further confirm the molecular states of both **Ag_252_** and **Ag_252_Zn_2_** with unpaired electrons on the basis of the superatom theory (for **Ag_252_**, the nominal shell electron count is 252 − 2 − 85 − 4 = 161), since an *S* = 1/2 spin state was revealed by the quantitative analysis for both NPs (Fig. S44), which is consistent with the molecular magnetism character rather than the metallic band’s Pauli magnetism one. Note that it was reported that relatively small NPs compared with **Ag_252_** displayed metallic behavior [2,29], which indicates that the size is not the sole decisive factor for the metallic behavior; however, the size increase definitely helps the transition from non-metallic to metallic behavior. For such a penta-twinned *fcc* structure, the detailed transition size waits to be further probed.

### Density functional theory (DFT) calculations

**Figure 4.**
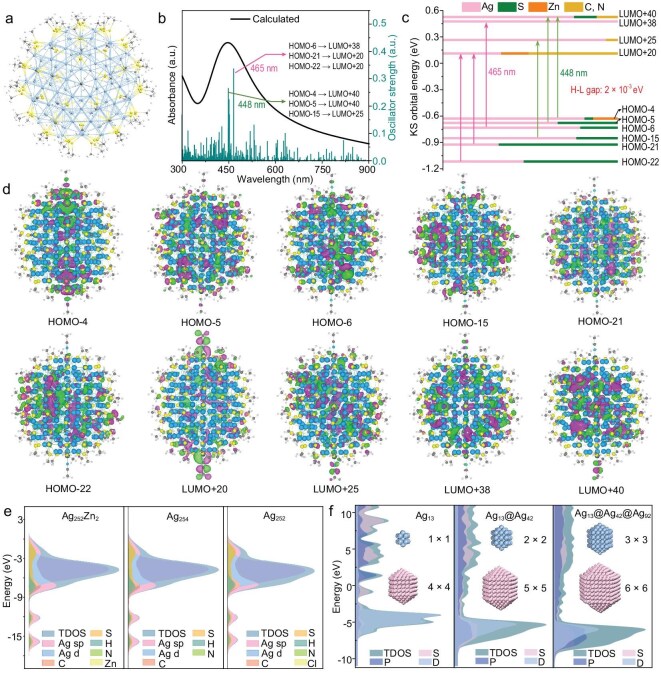
(a) Computational model of **Ag_252_Zn_2_** with all alkyl groups replaced with R = CH_3_. (b) Calculated UV–vis–near infrared (NIR) absorption spectrum of **Ag_252_Zn_2_**. (c) KS molecular energy level diagram and (d) the HOMO and LUMO distributions of **Ag_252_Zn_2_**. For clarity, only molecular orbitals corresponding to the absorption peaks are displayed. Additional orbital information can be found in Fig. S47 and Table S9. (e) Calculated angular momentum PDOS values for **Ag_252_Zn_2_, Ag_254_**(two Zn atoms in **Ag_252_Zn_2_** replaced by two Ag atoms) and **Ag_252_**. (f) PDOS of differently ordered gradient layers (Ag_13_, Ag_13_@Ag_42_ and Ag_13_@Ag_42_@Ag_92_) excluding ligand shell, with illustration of multiple predicted structures featuring high gradient layers. Color labels: light blue and pink, Ag; dark cyan, Zn; yellow, S; light cyan, N; dark green, Cl.

To interpret the absorption origin, theoretical calculations were performed. As shown in Fig. 4a and b, the steady-state absorption spectral profile and peak positions of **Ag_252_Zn_2_** are well reproduced via time-dependent DFT (TD-DFT) calculations [59–62]. Similarly, theoretical absorption spectra of **(AgZn)_44_** and **Ag_252_** exhibit sound agreement with corresponding experimental spectra (Figs S45 and S46). The consistency between the theoretical and experimental absorption profiles across all three NPs confirms the reliability of both TD-DFT calculations and atomic structures revealed via SCXRD (see Supplementary data for details). As shown in Fig. 4c, theoretical analysis of atomic orbital components in the frontier Kohn–Sham (KS) molecular orbitals (MOs) of **Ag_252_Zn_2_** reveals that the strongest oscillator strength at 465 nm originates from degenerate electronic transitions from highest occupied molecular orbital (HOMO)−6 → lowest unoccupied molecular orbital (LUMO)+38, HOMO−21 → LUMO+20, and HOMO−22 → LUMO+20. Similarly, the prominent oscillator strength at 448 nm arises from HOMO−4 → LUMO+40, HOMO−5 → LUMO+40, and HOMO−15 → LUMO+25 electronic transitions. The additional molecular orbitals can be found in Fig. S47 and Table S9. The HOMO–LUMO transitions are primarily distributed on the Ag and S atoms (Fig. 4c and d). It is worth noting that the d band, which varies in the energy range from −8.5 to 0 eV, contains the orbitals from HOMO−20 to HOMO−4 (Fig. S48). In addition, atomic orbital components of the frontier KS MOs of **(AgZn)_44_** and **Ag_252_** are analyzed (Figs S49–S53). For **(AgZn)_44_**, the sharp absorption peak at 405 nm is attributed to the degenerate transitions such as HOMO−2 → LUMO+5 and HOMO−1 → LUMO+4 (corresponding to 3p → 5sp excitation), while the prominent peak at 456 nm is assigned to HOMO−1 → LUMO+1, and HOMO−2 → LUMO electronic transitions (Figs S49 and S50). For **Ag_252_**, theoretical analysis reveals that the strongest oscillator strength at 475 nm originates from degenerate electronic transitions HOMO−58 → LUMO+38 and HOMO−28 → LUMO+51; the prominent oscillator strength at 450 nm arises from HOMO−8 → LUMO+74, and HOMO−6 → LUMO+77; the oscillator strength at 488 nm arises from HOMO−8 → LUMO+71, and HOMO−54 → LUMO+40 electronic transitions (Figs S51–S53). Although the transition orbitals of **Ag_252_** contributing to absorption centered at 465 nm differ from those of **Ag_252_Zn_2_**, their overall absorption profiles and excitation mechanisms remain highly similar. For **(AgZn)_44_, Ag_252_** and **Ag_252_Zn_2_**, the primary transition pathways all involve electron transitions from S to Ag atoms.

The total density of states (DOS) and partial DOS (PDOS) spectra of **Ag_252_Zn_2_** or **Ag_252_** based on the energy-optimized X-ray structure, reveal an extremely small HOMO–LUMO gap (*E*_g_) as indicated near the vertical dashed line at *E* − *E*_F_ = 0 (Fig. 4e), where *E*_F_ represents the Fermi level. For comparison, two Zn atoms in **Ag_252_Zn_2_** were replaced with two Ag atoms to calculate *E*_g_ and PDOS (Fig. 4e, Figs S54 and S55). The calculations yield *E*_g_ values of 2 × 10⁻^3^ eV for **Ag_252_Zn_2_**, 4.3 × 10⁻^3^ eV for **Ag_25__4_** and 6.5 × 10⁻^5^ eV for **Ag_252_**, all remarkably smaller than 1.52 eV for **(AgZn)_44_**. For **Ag_252_Zn_2_, Ag_25__4_** and **Ag_252_**, their *E*_g_ values are even smaller than *k*_B_*T* (*k*_B_: Boltzmann constant; *T*: temperature ≥ 298.15 K), indicating theoretical calculation deviations. Thus, compared with steady-state absorption and calculated *E*_g_, the pump-power dependence of TA dynamics widely used to determine the metallicity of Au NPs provides a more reliable criterion for determining the metallicity of Ag NPs. Notably, various metals possess distinct electron exchange and correlation effects, spin-orbit coupling etc. Therefore, extending to other metals from Au will reveal fundamental details about the emergence of the metallic state, and criteria may differ accordingly. Regardless of the experimental criterion employed, high-quality and sufficient samples are essential for reliable measurements. Incorrect samples can produce false information. For example, the coexistence of plasmon nanocrystals in molecular nanoclusters caused by instability and impurities can influence the excitation electron dynamics of the measured samples. In our case, the high stability and purity of measured samples ensure the reliability of measurement results, explaining why large-scale synthesis of metal-nanocluster single crystals is vital for fundamental research.

The relatively low energy of **Ag_252_Zn_2_** compared with **Ag_25__4_** explains the enhanced stability of **Ag_25__4_** after doping with two Zn atoms. The relatively low energy of **Ag_252_** compared with **Ag_252_Zn_2_** and **Ag_25__4_** indicates the influence of the ligand on the stability of metal nanoclusters. Further *Ab initio* Molecular Dynamics (AIMD) simulations confirmed that **Ag_252_** possessed the highest structural stability among the three, as evidenced by the smallest fluctuations in its Ag–Ag and Ag–S bond lengths. In contrast, **Ag_254_** exhibited the largest bond length fluctuations, indicating the lowest stability among the three investigated NPs (Fig. S56). The presence of μ₅-coordinated Cl atoms at the two terminal positions of **Ag_252_** contributes to the structural stabilization by achieving a lower energy state, as shown in Fig. S57. To evaluate the contribution of varying-order gradients to electronic structure, the DOS graphs of different metallic kernel-shell structures were calculated. As shown in Fig. 4f, the occupied orbital-to-unoccupied orbital transition primarily involves Ag *d* → Ag *sp* transition, providing *E*_g_ values of 8.0 × 10^−2^, 4.0 × 10^−2^ and 4.4 × 10^−3^ eV for Ag_13_, Ag_13_@Ag_42_ and Ag_13_@Ag_42_@Ag_92_ structures, respectively, demonstrating the influence of the gradient order on electronic transitions. The surprisingly low *E*_g_ values of bare Ag kernel-shell structures demonstrate the important influence of thiolate ligands on the *E*_g_ values of metal nanoclusters, which notably decreases as the shell number of kernel-shell structures increases. Based on this gradient structure, we predict high-order gradient layers (4 × 4 for Ag_162_, 5 × 5 for Ag_252_ and 6 × 6 for Ag_367_, as shown in the inset of Fig. 4g and Fig. S58) and potentially even more complex architectures, pending further experimental verification.

Although **Ag_252_Zn_2_** and **Ag_252_** are not metallic states but exhibit pseudo-plasma absorption bands (Fig. 1a), they show higher extinction coefficients (808.6 L·g^−1^·cm^−1^, 2.33 × 10^7^ M^−1^·cm^−1^ for **Ag_252_Zn_2_**; 648.1 L·g^−1^·cm^−1^, 1.95 × 10^7^ M^−1^·cm^−1^ for **Ag_252_**, Figs S59–S61) compared to ∼3.2 nm Ag nanocrystals (76.1 L·g^−1^·cm^−1^, Fig. S62) and **(AgZn)_44_** (9.2 L·g^−1^·cm^−1^ or 3.8 × 10^4^ M^−1^·cm^−1^, Figs S61 and S63) at 450 nm, indicating that the absorbance of metal NPs does not scale monotonically with the NP size, and metal NPs near the critical size may possess optimal absorption efficiency. In particular, an intriguing issue pertains to the influence of the solid state (crystalline state vs. amorphous state) of metal NPs on the photothermal performance, rarely investigated owing to crystallization challenges. The acquisition of large amounts of NP crystals enables this investigation. The photothermal conversion efficiencies for **Ag_252_Zn_2_, Ag_252_** and ∼3.2 nm Ag nanocrystals in toluene were determined to be 53.2%, 54.5% and 41.8%, respectively (Fig. S64 and Table S10). Although **Ag_252_** and **Ag_252_Zn_2_** show similar photothermal performance in solution (Fig. S64), the crystalline-state performance of **Ag_252_** outperformed that of **Ag_252_Zn_2_** (maximum temperature rise: 242.4°C vs. 210.7°C within 2 s, Fig. S65), indicating that a compact arrangement favors solid-state photothermy, as **Ag_252_** crystals have shorter interparticle H···H interaction distances than **Ag_252_Zn_2_** crystals (average 2.403 vs. 2.729 Å, Figs S66 and S67). This is further confirmed by stronger photothermy in the crystalline state than that in the amorphous state for all the three NPs: **(AgZn)_44_, Ag_252_** and **Ag_252_Zn_2_** (crystallization-induced enhancement of photothermy, Fig. S68). Another interesting finding is that both **Ag_252_Zn_2_** and **Ag_252_** show stronger photothermy than **(AgZn)_44_** and ∼3.2 nm Ag NPs, indicating that the photothermy of metal NPs does not scale monotonically with the NP size. NPs near the critical size may have optimal photothermal effects, which manifests the uniqueness of these critical-size NPs and their potential for practical applications.

## CONCLUDING REMARKS

In summary, by introducing an anti-galvanic doping method, we for the first time achieve gram-scale, high-yield synthesis of a giant Ag–Zn nanocluster [Ag_252_Zn_2_(3,5-DMBT)_85_(MeCN)_2_, totaling 1796 atoms] and grow high-quality single crystals (2.4011 g, 52.8% yield) suitable for SCXRD. Furthermore, we successfully performed atomically precise de-alloying of the bimetal nanocluster via ligand exchange, and grew high-quality mono-Ag nanocluster single crystals at a gram scale (1.0303 g, 21.3% yield), demonstrating the feasibility of synthesizing and tuning giant metal nanoclusters with atomic precision, large scale and high yield. Their atomic structures were well resolved using SCXRD data with low residual factor *R*_1_ (5.7% for **Ag_252_Zn_2_** and 3.6% for **Ag_252_**). Interestingly, these NPs feature similar penta-twinned *fcc* structures and perfect shape–number characteristics (0 × 0, 1 × 1, 2 × 2 and 3 × 3) in multiple directions, based on which high shape–number layers (4 × 4, 5 × 5 and 6 × 6) were predicted. Despite low *E*_g_ values (<*k*_B_*T, T* ≥ 298.15 K) and plasmon-like absorptions (centered at 460 nm for **Ag_252_Zn_2_** and 465 nm for **Ag_252_**), the as-obtained NPs remain in molecular state as definitively determined via TA measurements, revealing that steady-state absorption and theoretical calculations are less reliable than power-dependent electron excitation dynamics. Furthermore, absorbency (photothermy) of metal NPs does not positively correlate with the NP size; metal NPs near the critical size exhibit optimal absorption efficiency (photothermal effect) compared to investigated small nanoclusters and large nanocrystals. Additionally, crystallization-induced enhancement of photothermy for metal NPs was first revealed. Other achievements include the first successful doping of active abundant metal Zn atoms into Ag nanoclusters with enhanced stability, and atomically precise transformation from subnanometer nanoclusters to >2 nm nanoclusters, paving the way for precise manufacturing from subnanomaterials with the well-defined compositions and structures. Thus, this study is expected to advance the synthesis and tuning of giant metal nanoclusters, and studies of critical size and beyond.

## MATERIALS AND METHODS

Details of the Materials and Methods are provided in the Supplementary data.

## Supplementary Material

nwag001_Supplemental_Files

## Data Availability

Experimental details, computational details, detailed crystallographic structure and data including the CIF file, ESI-MS and UV/vis/NIR are available within the article and its Supplementary data files. Other relevant data are available from the corresponding author upon request. The X-ray crystallographic coordinates for structures reported in this article have been deposited at the Cambridge Crystallographic Data Centre, under deposited numbers CCDC 2518296, 2518300 and 2518303 for **(AgZn)_44_, Ag_252_Zn_2_** and **Ag_252_**, respectively. These data can be obtained free of charge from the Cambridge Crystallographic Data Centre via www.ccdc.cam.ac.uk/data_request/cif.
