# Supplementary material for: Molecular metal nanoparticles with pseudo-plasma absorption band
Source: Natl Sci Rev. 2026 Jan 10;13(4):nwag001. doi: 10.1093/nsr/nwag001 (PMC12906343; doi:10.1093/nsr/nwag001)
Supplement: nwag001_Supplemental_Files [file nwag001_supplemental_files.zip › nwag001-Suppoting inforamtion-updated.pdf]

Supplementary Information (SI)

Molecular metal nanoparticles with pseudo-plasma absorption band

Liang Fang[1,2,3]†, Rong Liu[4]†, Zongbing He[1,2,3]†, Guowei Guan[1,2,3]†, Lingwen Liao[1,2,3], Runguo Wang[1,2,3], Wanmiao Gu[1,2,3], Chengming Wang[5], Jin Li[6], Haiteng Deng[7], Shengli Zhuang[1,2,3]*, Zhou Lu[4]*, and Zhikun Wu[1,2,3]*

[1]Key Laboratory of Materials Physics, Anhui Key Laboratory of Nanomaterials and Nanotechnology, CAS Center for Excellence in Nanoscience, Institute of Solid State Physics, HFIPS, Chinese Academy of Sciences, Hefei, 230031, P. R. China.

[2]Institute of Physical Science and Information Technology, Anhui University, Hefei, 230601, P. R. China.

[3]Key Laboratory of Precision and Intelligent Chemistry, University of Science and Technology of China, Hefei, 230026, P. R. China.

[4]Anhui Province Key Laboratory for Control and Applications of Optoelectronic Information Materials, School of Physics and Electronic Information, the Key Laboratory of Functional Molecular Solids, Ministry of Education, Anhui Normal University, Wuhu, Anhui, 241002, P. R. China.

[5]Instruments' Center for Physical Science, University of Science and Technology of China, Hefei, 230026, P. R. China.

[6]Tsinghua University-Peking University Joint Center for Life Sciences, School of Life Sciences, Tsinghua University, Beijing, 100084, P. R. China.

[7]MOE Key Laboratory of Bioinformatics, School of Life Sciences, Tsinghua University, Beijing, 100084, P. R. China.

*Shengli Zhuang. Email: slzhuang@issp.ac.cn.

*Zhou Lu. Email: zhoulu@ahnu.edu.cn.

*Zhikun Wu. Email: zkwu@issp.ac.cn.

†These authors contributed equally to this work.

Contents

Materials and Methods 5
Chemical 5
Synthesis of $(AgZn)_{44}$ 5
Synthesis of $Ag_{252}Zn_2$ 5
Synthesis of Ag_{252} 6
Gram-level synthesis of $Ag_{252}Zn_2$ 6
Gram-level synthesis of Ag_{252} 7
Synthesis of ~3.2 nm Ag nanoparticles 8
Ultrafast transient absorption measurements 8
Characterization 8
Computational Methods 9

Supplementary Figures

Figure S1. Schematic representation of the syntheses of $(AgZn)_{44}$, $Ag_{252}Zn_2$, and Ag_{252} 11
Figure S2. PTLC plate of $(AgZn)_{44}$. 12
Figure S3-S5. Positive-ion mode ESI-MS spectrum of $Ag_{32}Zn_{12}(TPP)_{4\text{-}x}(3,5\text{-DMBT})_{36}$. 13
Figure S6. Crystal structure and particle dimensions 16
Figure S7. Thermogravimetric analysis 17
Figure S8. The XPS spectra of $(AgZn)_{44}$, $Ag_{252}Zn_2$, and Ag_{252} 18
Figure S9. ESI-MS spectrum of Ag_{252} 19
Figure S10. ESI-MS spectrum of Ag_{252} 20
Figure S11. The IR spectra of $AgNO_3$, $(AgZn)_{44}$, $Ag_{252}Zn_2$, and Ag_{252} 21
Figure S12. Particle size and distribution of $(AgZn)_{44}$ 22
Figure S13. Particle size and distribution of $Ag_{252}Zn_2$. 23
Figure S14. Particle size and distribution of Ag_{252} 24
Figure S15. Kernel growth pattern of $Ag_{32}Zn_{12}$ from the inside to outside 25
Figure S16. The triangular numbers structures composed of different atoms 26
Figure S17. Crystal structure of $(AgZn)_{44}$ 27
Figure S18. Crystal structure of $Ag_{252}Zn_2$ 28
Figure S19. Crystal structure of Ag_{252} 29
Figure S20. Ag–Ag bond length distributions in the metal kernel of the $Ag_{252}Zn_2$ nanoparticle.. 30
Figure S21. Ag–Ag bond length distributions in the metal kernel of the Ag_{252} nanoparticle 31
Figure S22. Top view of the apical staple structure dissection 32
Figure S23. Staple structures 33
Figure S24. Comparison of bond length 34
Figure S25. Crystal structure of Ag_{252} 35
Figure S26. Comparison of thermal stability 36
Figure S27. Comparison of thermal stability 37
Figure S28. UV-Vis-NIR absorption spectra of $(AgZn)_{44}$, $Ag_{252}Zn_2$ and Ag_{252}. 38
Figure S29. Particle size and distribution of ~3.2 nm Ag nanocrystals 39
Figure S30. Absorption difference spectra and emission spectra 40
Figure S31. TA maps of $(AgZn)_{44}$ under 400 nm excitation with various pump powers 41
Figure S32. TA maps of $Ag_{252}Zn_2$ under 400 nm excitation with various pump powers 42
Figure S33. TA maps of Ag_{252} under 400 nm excitation with various pump powers. 43

Figure S34. TA maps of ~3.2 nm Ag nanocrystals under 400 nm excitation with various pump powers. 44
Figure S35. Kinetic curves of $(AgZn)_{44}$ probed at 430 nm under 400 nm excitation. 45
Figure S36. Kinetic curves of $Ag_{252}Zn_2$ probed at 460 ± 30 nm under 400 nm excitation. 46
Figure S37. Kinetic curves of Ag_{252} probed at 460 nm under 400 nm excitation. 47
Figure S38. Kinetic curves of ~3.2 nm Ag nanocrystals probed at 450 nm under 400 nm excitation 48
Figure S39. TA maps of $Ag_{252}Zn_2$ under 500 nm excitation with various pump powers. 49
Figure S40. TA maps of $Ag_{252}Zn_2$ under 600 nm excitation with various pump powers 50
Figure S41. Kinetic curves of $Ag_{252}Zn_2$ probed at 460 ± 30 nm under 500 nm excitation 51
Figure S42. Kinetic curves of $Ag_{252}Zn_2$ probed at 460 ± 30 nm under 600 nm excitation 52
Figure S43. Kinetic curves of $Ag_{252}Zn_2$ probed at different wavelengths under 400 nm excitation. 53
Figure S44. EPR measurement of $Ag_{252}Zn_2$ and Ag_{252} and Au_{25} 54
Figure S45. DFT calculations of $(AgZn)_{44}$ 55
Figure S46. DFT calculations of Ag_{252} 56
Figure S47. The distributions of $Ag_{252}Zn_2$ from HOMO-22 to LUMO+40 57
Figure S48. TDOS of $Ag_{252}Zn_2$ 58
Figure S49. Kohn–Sham molecular energy level diagram of $(AgZn)_{44}$ 59
Figure S50. HOMO and LUMO distributions of $(AgZn)_{44}$ 60
Figure S51. Kohn–Sham molecular energy level diagram of Ag_{252} 61
Figure S52. HOMO distributions of Ag_{252} 62
Figure S53. LUMO distributions of Ag_{252} 63
Figure S54. Computational models 64
Figure S55. HOMO and LUMO distributions of $Ag_{252}Zn_2$, Ag_{254}, and Ag_{252} 65
Figure S56. Radial distribution function of Ag_{252}, $Ag_{252}Zn_2$ and Ag_{254} 66
Figure S57. AIMD simulation 67
Figure S58. The progressive order gradient layers corresponds to the decreasing trend of E_g 68
Figure S59. Optical properties of $Ag_{252}Zn_2$ 69
Figure S60. Optical properties of Ag_{252} 70
Figure S61. The relationship between molar extinction coefficients and numbers of Ag atoms. 71
Figure S62. Optical properties of ~3.2 nm Ag nanocrystals 72
Figure S63. Optical properties of $(AgZn)_{44}$ 73
Figure S64. Photothermal properties 74
Figure S65. Temperature changes during irradiation treatment 75
Figure S66. The average ligand H···H interaction distance between two adjacent $Ag_{252}Zn_2$ particles 76
Figure S67. The average ligand H···H interaction distance between two adjacent Ag_{252} particles. 77
Figure S68. Time-dependent temperature changes of amorphous $(AgZn)_{44}$, $Ag_{252}Zn_2$, Ag_{252}, and ~3.2 nm Ag nanocrystals 78

Supplementary Tables

Table S1. Partial characteristic information associated with the reported, structure-resolved nanoparticles with metal atom number larger than 100 per particle 79
Table S2. Bond length analyses of the metal kernel of the $Ag_{252}Zn_2$ nanoparticle 82

Table S3. Bond length analyses of the metal kernel of the Ag_{252} nanoparticle 83
Table S4. Ag–Ag/Ag–S bond length analyses of the staples in the $Ag_{252}Zn_2$ nanoparticle. 84
Table S5. Ag–Ag/Ag–S bond length analyses of the staples in the Ag_{252} nanoparticle 85
Table S6. Kinetic fitting results. 86
Table S7,S8. Kinetic fitting results of $Ag_{252}Zn_2$ 87
Table S9. The orbital distribution of $Ag_{252}Zn_2$ 90
Table S10. Calculation of photothermal conversion efficiency 92
Table S11. The ligand H· · · H interaction distances between two adjacent particles. 93
Table S12. Crystal data and structure refinement for $(AgZn)_{44}$ 94
Table S13. Crystal data and structure refinement for $Ag_{252}Zn_2$ 95
Table S14. Crystal data and structure refinement for Ag_{252} 96
References 97

Materials and Methods

Chemical

All chemicals are commercially available and used as received. Silver nitrate ($AgNO_3$, 99.9%) and zinc nitrate hexahydrate ($Zn(NO_3)_2 \cdot 6H_2O$, 99.99%) were both purchased from Sigma-Aldrich. 3,5-Dimethylbenzenethio ($C_8H_{10}S$, 97.0%), 4-tert-butylbenzenethiol ($C_{10}H_{14}S$, 97.0%), triphenylphosphine ($C_{18}H_{15}P$, 99.0%) and sodium borohydride ($NaBH_4$, 98.0%) were purchased from Aladdin Reagent Shanghai Co., Ltd. Dichloromethane (CH_2Cl_2, 99.0%), tetrahydrofuran (C_4H_8O, 99.8%), toluene (C_7H_8, 99.5%), acetonitrile (CH_3CN, 99.99%) and methanol (CH_3OH, 99.5%) were purchased from Sinopharm Chemical Reagent Co., Ltd.

Synthesis of $(AgZn)_{44}$

$AgNO_3$ (50 mg) and $Zn(NO_3)_2 \cdot 6H_2O$ (150 mg) were dissolved in a mixed solvent of methanol (6 mL) and tetrahydrofuran (6 mL). Then, 3,5-dimethylbenzenethio (3,5-DMBT, 100 μL) and triphenylphosphine (TPP, 20 mg) were added to the solution cooled in an ice bath, causing the color to change from colorless transparent to yellow. After vigorous stirring for 30 min, ice-cold aqueous $NaBH_4$ (50 mg in 1 mL of water) was quickly added. The solution color slowly changed from yellow to light brown within 20 min. Subsequently, ice-cold aqueous $NaBH_4$ (150 mg in 2 mL of water) was slowly added, and the reaction mixture was stirred for 6 h to yield a brownish-black solution. The resultant crude was washed repeatedly with methanol and then dissolved in dichloromethane (3 mL). $(AgZn)_{44}$ nanoclusters were isolated via PTLC (eluent: dichloromethane/petroleum ether = 4/1, *v*/*v*), and then crystallized via diffusion in a toluene/acetonitrile (*v*/*v*, 6:1) mixed solvent over 10 days. The yield of the crystals was ~1.6% (based on Ag). The as-obtained crystals were kept protected from light before any tests.

Synthesis of $Ag_{252}Zn_2$

$AgNO_3$ (50 mg) and $Zn(NO_3)_2 \cdot 6H_2O$ (150 mg) were dissolved in a mixed solvent of methanol (6 mL) and tetrahydrofuran (6 mL) and then 3,5-DMBT (100 μL) and TPP (20 mg) were added to the solution cooled in an ice bath, causing the color to change from colorless transparent to yellow. After vigorous stirring for 30 min, ice-cold aqueous $NaBH_4$ (50 mg in 1 mL of water) was quickly added. The solution color slowly changed from yellow to light brown within 20 min. Then, ice-cold aqueous $NaBH_4$ (150 mg in 2 mL of water) was slowly added, and the reaction mixture was stirred for 6 h to yield a brownish-black solution. The resultant

precipitates by adding excess methanol were washed multiple times, then dissolved in a mixed solvent of toluene (8 mL) and dichloromethane (2 mL), into which 3,5-DMBT (60 μL) was added under 60°C. After 6 h of the reaction, the in-process products were precipitated, washed with methanol, acetonitrile, and water multiple times, then dissolved in mixed solvents of toluene (8 mL) and dichloromethane (2 mL) and heated under 60°C for 5 h. The resultant crude was washed repeatedly with methanol, acetonitrile, and dichloromethane and then dissolved in toluene (3 mL). After 5-7 days, the crystals were formed via diffusion with acetonitrile at room temperature. The yield of the crystals was ~53% (based on Ag).

Synthesis of Ag_{252}

$AgNO_3$ (50 mg) and $Zn(NO_3)_2·6H_2O$ (150 mg) were dissolved in a mixed solvent of methanol (6 mL) and tetrahydrofuran (6 mL), and then 3,5-DMBT (100 μL) and TPP (20 mg) were added to the solution in an ice bath, causing the resulting solution color to change from colorless transparent to yellow. After vigorous stirring for 30 min, ice-cold aqueous $NaBH_4$ (50 mg in 1 mL water) was quickly added. The solution color slowly changed from yellow to light brown within 20 min. Then, ice-cold aqueous $NaBH_4$ (150 mg in 2 mL of water) was slowly added, and the reaction mixture was stirred for 6 h to yield a brownish-black solution. The resultant precipitates, formed by adding excess methanol were washed multiple times, then dissolved in the mixed solvent of toluene (8 mL) and dichloromethane (2 mL), into which 3,5-DMBT (60 μL) was added at 60°C. After 6 h, the in-process products were precipitated, washed with methanol, acetonitrile, and water multiple times, and then again dissolved in mixed solvents of toluene (8 mL) and dichloromethane (2 mL) and heated at 60°C for 5 h. The as-obtained precipitates were washed with methanol, acetonitrile and dichloromethane multiple times and then dissolved in toluene (8 mL) and dichloromethane (2 mL) containing 4-tert-butylbenzenethiol (TBBT, 1 mL), and heated at 80°C for 12. The crude product was isolated, washed with methanol and then dissolved in toluene (3 mL). After 3–5 days, the crystals were obtained via diffusion with acetonitrile at room temperature, yielding ~22% (based on Ag).

Gram-level synthesis of $Ag_{252}Zn_2$

$AgNO_3$ (5 g) and $Zn(NO_3)_2·6H_2O$ (15 g) were dissolved in a mixed solvents of methanol (200 mL) and tetrahydrofuran (200 mL) in a volume ratio of 1:1, and then 3,5-DMBT (10 mL) and TPP (2 g) were added to the solution cooled in an ice bath, causing the color to change from

colorless transparent to yellow. After vigorous stirring for 1 h, ice-cold aqueous $NaBH_4$ (5 g in 20 mL of water) was slowly added. The solution color slowly changed from yellow to light brown within 1 h. Subsequently, ice-cold aqueous $NaBH_4$ (15 g in 30 mL of water) was slowly added, and the reaction mixture was stirred for 12 h to yield a brownish-black solution. The resultant precipitates by adding excess methanol were washed repeatedly, then dissolved in a mixed solvents of toluene (80 mL) and dichloromethane (20 mL), into which 3,5-DMBT (6 mL) was added at 60°C. After 24 h, the in-process products were precipitated, washed repeatedly with methanol, acetonitrile, and water and then dissolved in mixed solvents of toluene (80 mL) and dichloromethane (20 mL) and heated at 60°C for 24 h. The resultant crude was washed repeatedly with methanol, acetonitrile and dichloromethane and then dissolved in toluene (150 mL). After 21 days, crystals were formed via diffusion with acetonitrile at room temperature,weighing 2.41g with a yield of~52.8% (based on Ag).

Gram-level synthesis of Ag_{252}

$AgNO_3$ (5 g) and $Zn(NO_3)_2 \cdot 6H_2O$ (15 g) were dissolved in a mixed solvent of methanol (200 mL) and tetrahydrofuran (200 mL) in a volume ratio of 1:1, and 3,5-DMBT (10 mL) and TPP (2 g) were added to the solution cooled in an ice bath, causing color to change from colorless transparent to yellow. After vigorous stirring for 1 h, ice-cold aqueous $NaBH_4$ (5 g in 20 mL of water) was slowly added. The solution color slowly changed from yellow to light brown within 1 h. Subsequently, ice-cold aqueous $NaBH_4$ (15 g in 30 mL of water) was slowly added, and the reaction mixture was stirred for 12 h to yield a brownish-black solution. The resultant precipitates, formed by adding excess methanol were washed repeatedly, then dissolved in mixed solvents of toluene (80 mL) and dichloromethane (20 mL), into which 3,5-DMBT (6 mL) was added at 60°C. After 24 h , the in-process products were precipitated, washed repeatedly with methanol, acetonitrile, and water and then dissolved in mixed solvents of toluene (80 mL) and dichloromethane (20 mL) and heated at 60°C for 24 h. The as-obtained precipitates, formed by adding excess methanol, were washed repeatedly with methanol, and then dissolved in toluene (100 mL) and dichloromethane (20 mL) containing TBBT (100 mL) and heated at 80°C for 32 h. The crude was isolated, washed repeatedly with methanol, and then dissolved in toluene (150 mL). After 20–25 days, crystals were obtained via diffusion with acetonitrile at room temperature, weighing 1.03 g with a yield of ~21.3% (based on Ag).

Synthesis of ~3.2 nm Ag nanoparticles

$AgNO_3$ (50 mg) was dissolved into a mixed solvent of methanol (4 mL) and tetrahydrofuran (8 mL). The resulting solution was cooled to 0°C in an ice bath over 20 min, into which 3,5-DMBT (160 μL) was slowly added. After 1 h, an aqueous $NaBH_4$ solution (110 mg in 2 mL of water) was rapidly added at once under vigorous stirring. The reaction proceeded with constant stirring for 6 h. The precipitates formed by adding excess methanol were washed repeatedly with methanol and water, then dried under reduced pressure at room temperature. Toluene was added to them, and after the removal of insoluble solids, an adequate amount of methanol was added until extensive precipitation appeared. The precipitates were collected and dried for subsequent experiments.

Ultrafast transient absorption measurements

Herein, fs-TA spectra were acquired with a typical transmission pump-probe setup. A Ti: sapphire regenerative amplifier (Spitfire Ace, Spectra Physics, Inc.) produced laser pulses (6 mJ, 1 kHz) with a pulse width of 80 fs centered at 800 nm. The laser output was then split into several beams, one of which generated wavelength-tunable light serving as the pump laser for TA measurement via a downstream optical parametric amplifier (TOPAS-Prime). Herein, 400, 500, and 600 nm were separately chosen as the center wavelength of the TOPAS-Prime output. The pump laser was attenuated to the specified power and modulated using a 500 Hz mechanical chopper. Another laser beam propagated through an optical delay line (0–8 ns) before focusing on CaF_2 crystal to produce white light continuum as the probe light in fs-TA. Pump and probe light overlapped in the sample cell with an optical path of 2 mm. All experiments were conducted at room temperature.

Characterization

All UV-Vis absorption spectra were acquired in the range of 200 nm to 900 nm using a Shimadzu UV2550 spectrophotometer and in the range of 200 nm to 1600 nm using a Solid 3700 DUV Spectrophotometer. Electrospray ionization mass spectra (ESI-MS) were acquired on a Waters Q-TOF mass spectrometer equipped with a Z-spray source. The sample was dissolved in CH_3OH or C_7H_8.The single crystal X-ray diffraction (SCXRD) data was collected on a Bruker D8 Venture X-ray diffractometer (Bruker, Germany) with a Helios mx multilayer monochromator and Cu Kα radiation (λ = 1.54178 Å). Thermogravimetric analysis (TGA) (~ 3

mg sample used) was conducted in a TG/DTA 6300 thermal analyzer at a scanning rate of 10°C/min under N_2 atmosphere. X-ray photoelectron spectroscopy (XPS) measurements were performed on an ESCALAB 250Xi XPS spectrometer (Al Ka, hv = 1486.6 eV) using a monochromatized Al Kα source equipped with an Ar^+ ion sputtering gun. All binding energies were calibrated using the C (1s) peak (284.8 eV). Fourier transform infrared spectrometer (FTIR) spectra were analyzed on a Nicolet 8700 FTIR spectrometer from KBr pellets as the sample matrix. Morphologies of the nanoparticles were measured using a transmission electron microscope (TEM) with Tecnai TF-20 equipment of Thermofisher. The photoluminescent spectra were obtained by FLIM300 microscopic fluorescence spectrometer (Dalian Time-Tech Spectra, China). EPR spectra were collected using a Bruker EMX plus 10/12 (equipped with Oxford ESR910 Liquid Helium cryostat). Photothermal measurements were conducted using a 450 nm (LSR450SD-PS-II) laser. The temperature was measured with a thermal imager infrared camera (Fotric 227S). Infrared photos and real-time temperatures were extracted from the video by FLIR tools software.

Computational Methods

To investigate these large systems, the thiol ligand was simplified to $–SCH_3$ to reduce computational cost. Excited-state calculations were performed using the simplified Tamm-Dancoff approximation (sTDA) in the ORCA quantum chemistry package, enabling extraction of absorption spectra and detailed information on individual excited states [1,2]. For orbital composition and component analyses of the cluster, the CP2K program was employed [3], utilizing the PBE functional and DZVP-MOLOPT-SR-GTH basis set, with a convergence threshold of 5×10^{-5}. Wavefunction files were then generated for further analysis, and both output and wavefunction files were analyzed using Multiwfn software [4,5]. DOS and PDOS were computed by using Gaussian 16 program (Revision B01) [6]. Ab initio Molecular Dynamics (AIMD) simulation details: molecular dynamics were performed with CP2K 2025.1 in a periodic box of dimensions 37.35 × 37.25 × 34.31 Å. The SCF convergence threshold was set to 1.0×10^{-4}. The xTB1 semi-empirical method with DFT-D3 dispersion correction was employed to model the nanocluster system. Simulations were carried out at 298.15 K and 1 atm. A time step of 1 fs was used, and the trajectory comprised 30000 steps. Quantum chemistry calculations used the DFT-PBE method [7], with the D3 version of Grimme's dispersion correction including Becke–Johnson damping function [8]. The double-zeta Turbomole series

basis set, Def2-SV(P), was used for all atoms [9]. The DOS and PDOS were generated using the Multiwfn (Version 3.8 (dev)).

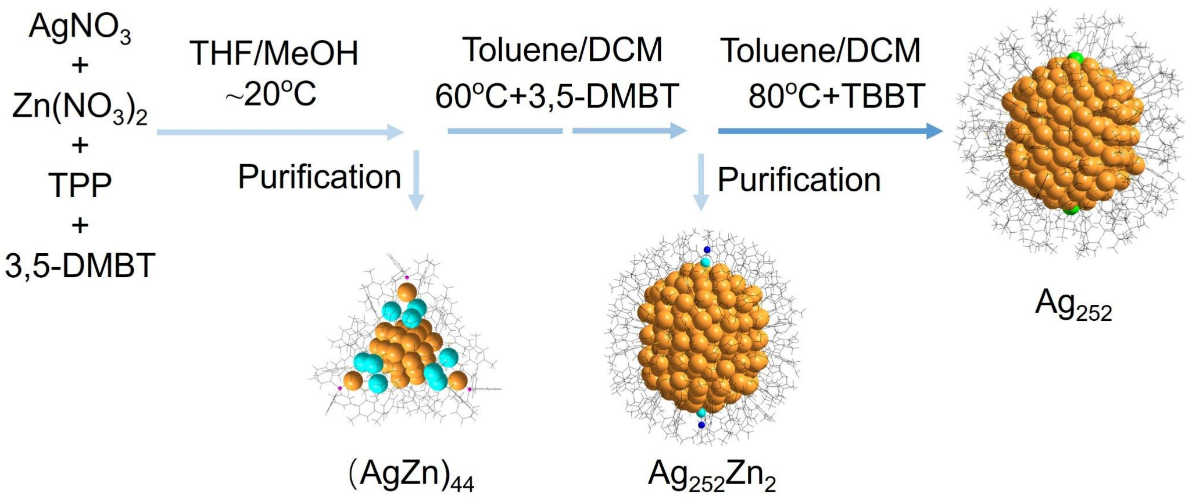

Figure S1. Schematic representation of the syntheses of $(AgZn)_{44}$, $Ag_{252}Zn_2$, and Ag_{252}.

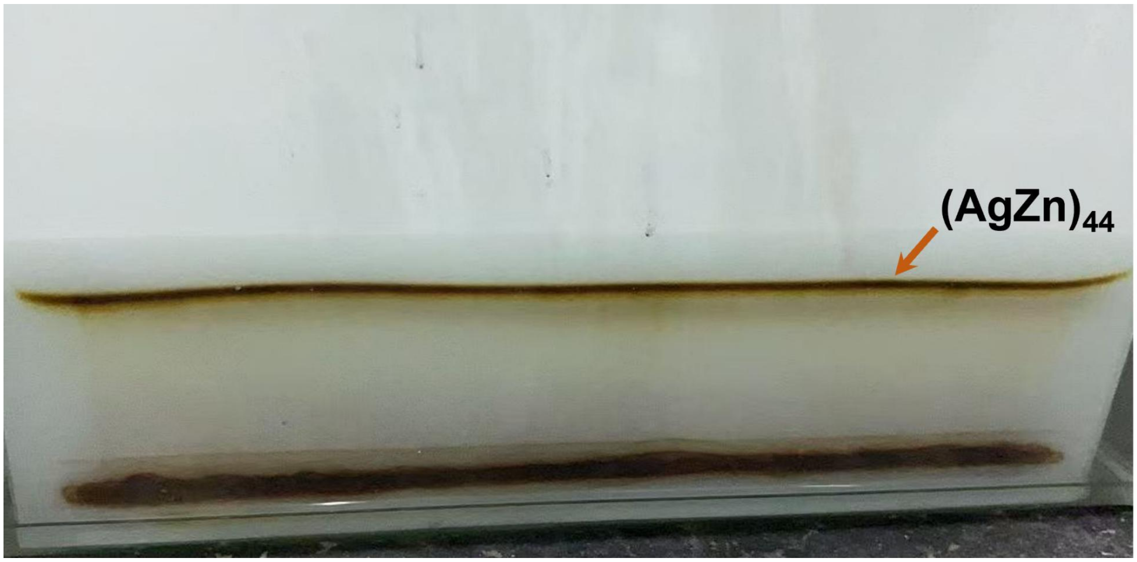

Figure S2. PTLC plate of $(AgZn)_{44}$.

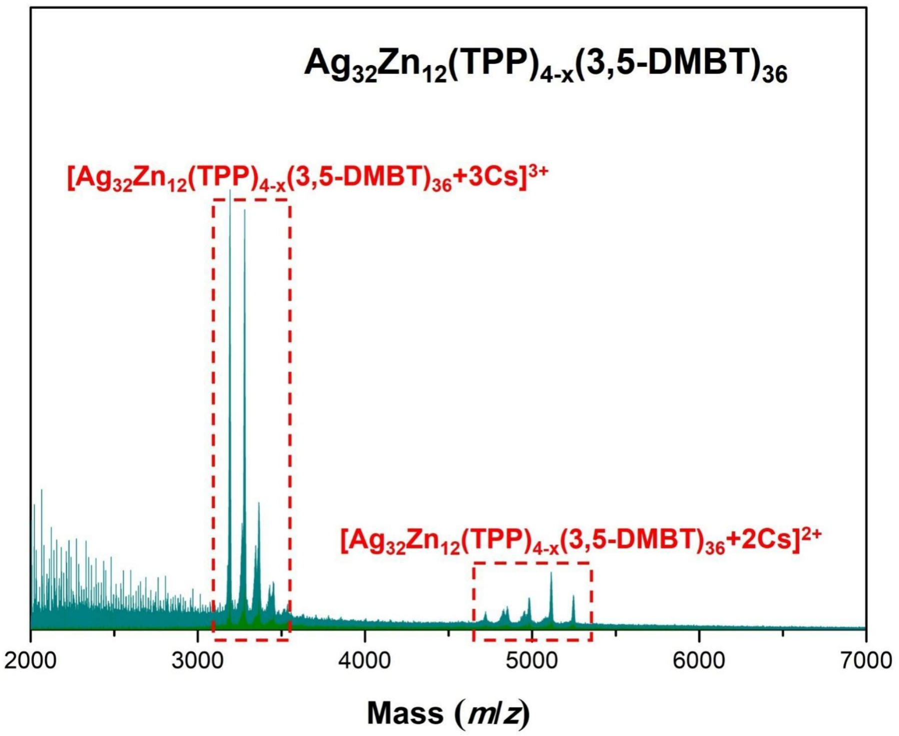

Figure S3. Positive-ion mode ESI-MS spectrum of $Ag_{32}Zn_{12}(TPP)_{4\text{-}x}(3,5\text{-DMBT})_{36}$.

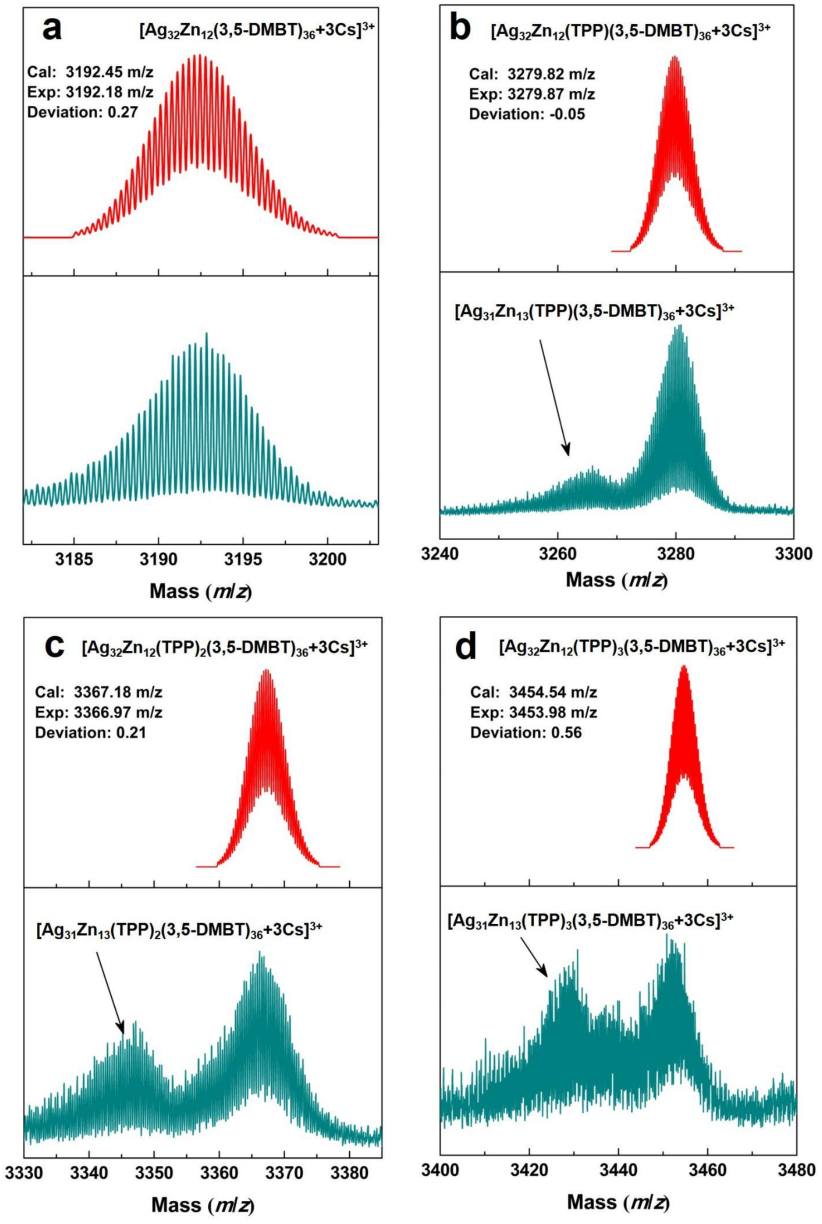

Figure S4. **(a−d) Zoom-in experimental isotope patterns of $[Ag_{32\text{-}y}Zn_{12+y}(TPP)_{4\text{-}x}(3,5\text{-}DMBT)_{36}+3Cs]^{3+}$ ($1 \leq x \leq 4$, y = 0 and 1).**

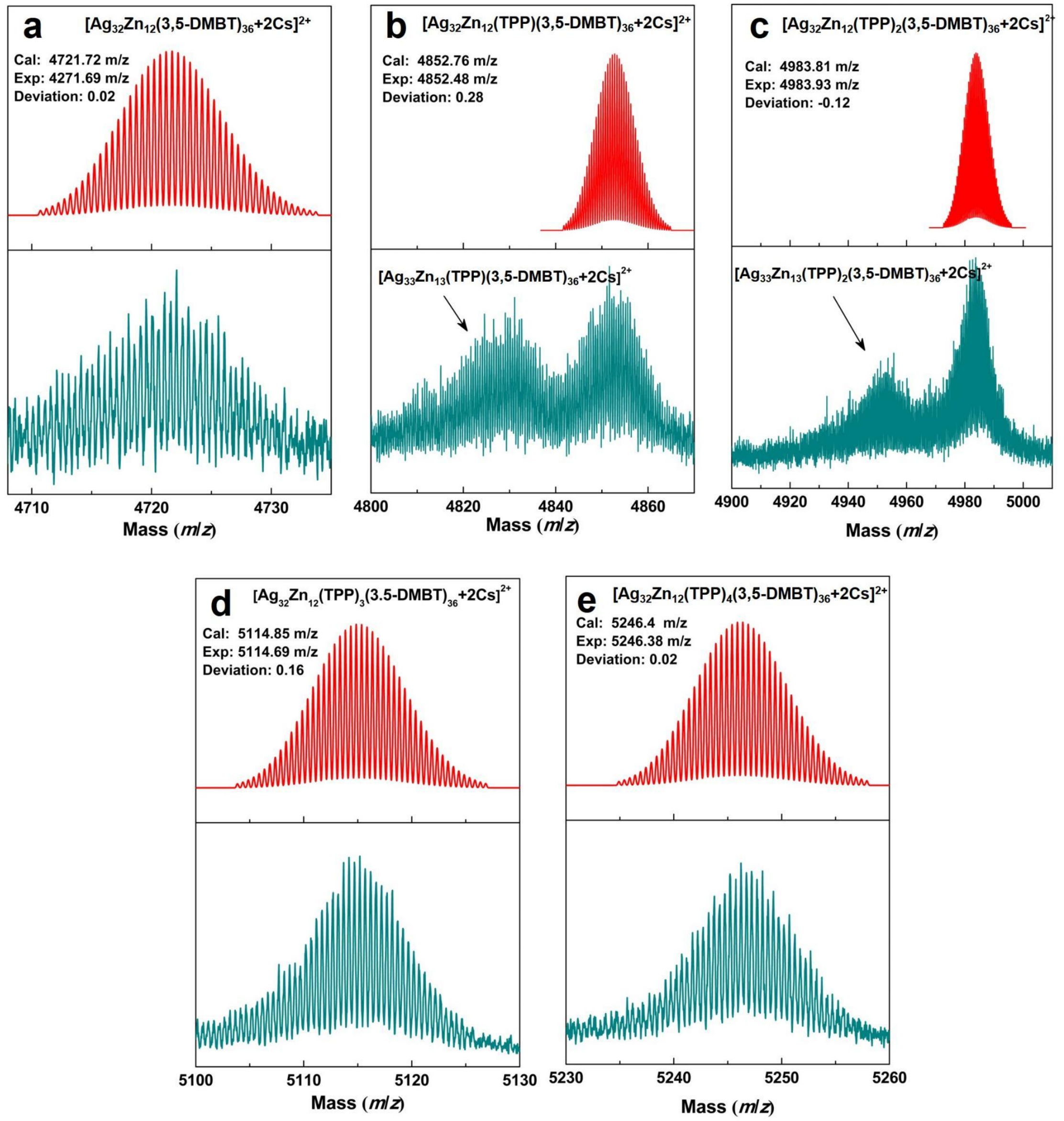

Figure S5. (a−e) Zoom-in experimental isotope patterns of $[Ag_{32-y}Zn_{12+y}(TPP)_{4-x}(3,5\text{-}DMBT)_{36}+2Cs]^{2+}$ ($0 \leq x \leq 4$, y = 0 and 1).

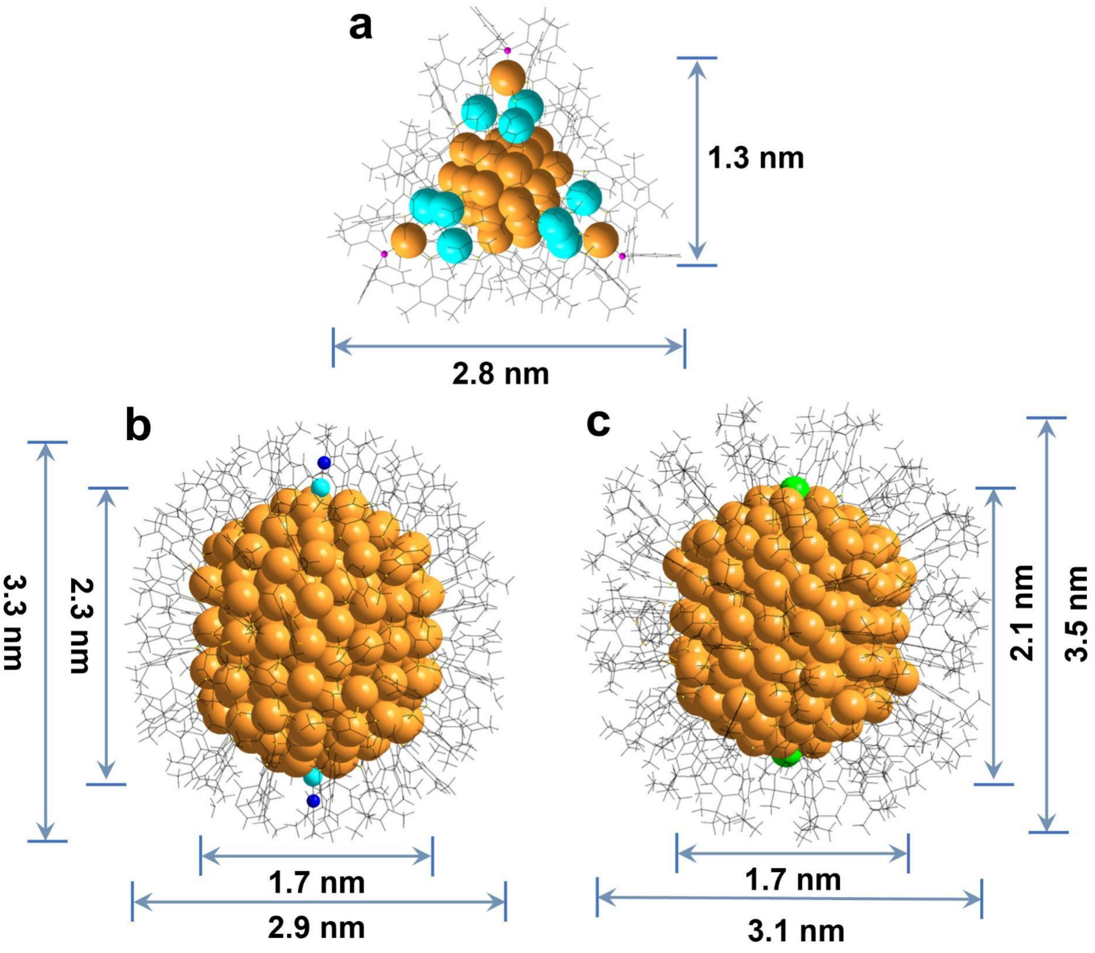

Figure S6. Crystal structure and particle dimensions. Particle dimensions of (a) $(AgZn)_{44}$, (b) $Ag_{252}Zn_2$, and (c) Ag_{252} with the consideration of metal kernel and ligand shell. Color labels: gold, light blue, Ag; light green, Cl; blue, N; gray, C, S, and H.

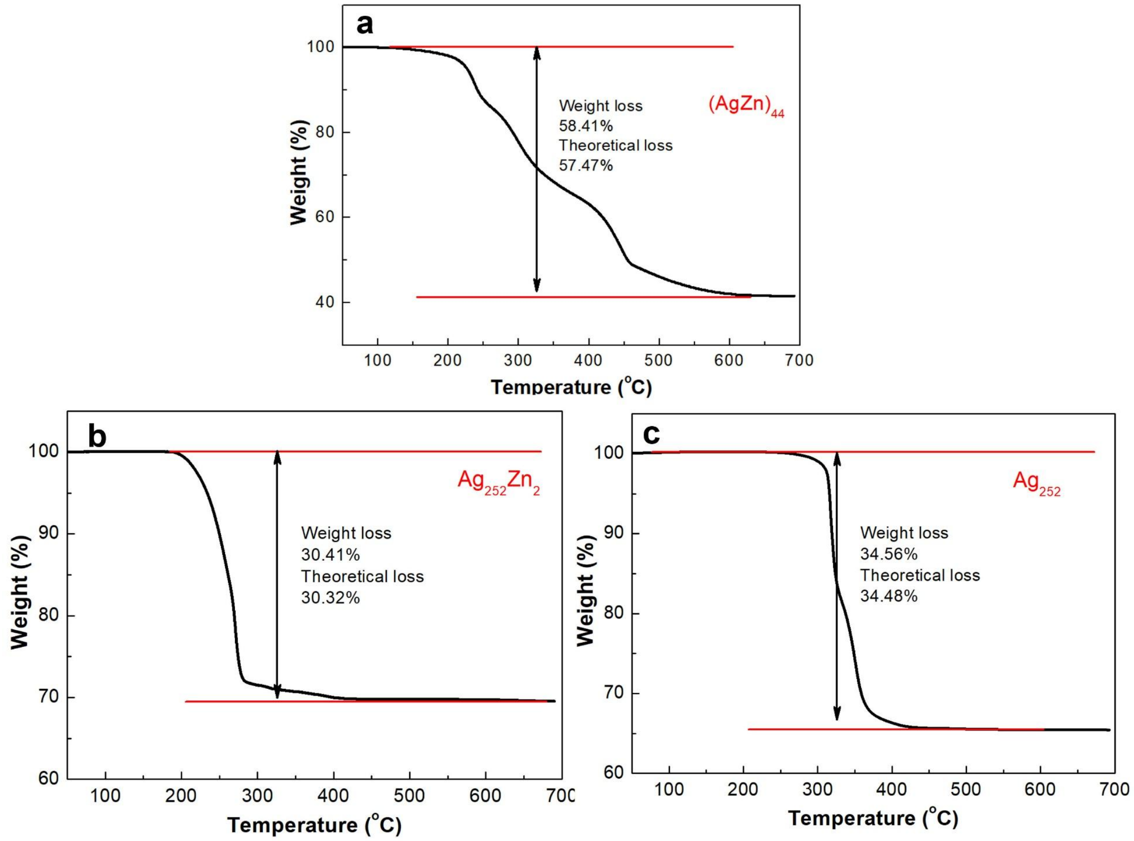

Figure S7. Thermogravimetric analysis (TGA) of (a) $(AgZn)_{44}$, (b) $Ag_{252}Zn_2$, and (c) Ag_{252} in N_2 atmosphere. The theoretical loss is calculated based on the particle molecular weights and the complete removement of ligands.

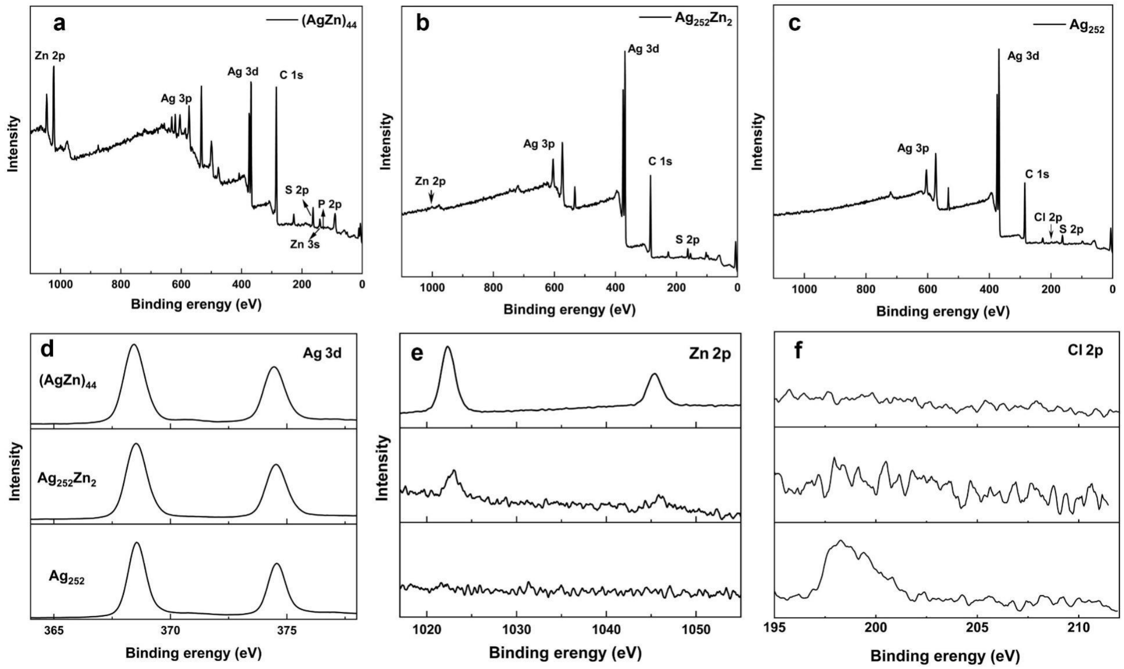

Figure S8. The XPS spectra of (a) $(AgZn)_{44}$, (b) $Ag_{252}Zn_2$, and (c) Ag_{252}. (a−c) Survey spectra; (d−f) Comparison of high-resolution XPS spectra for Ag 3d, Zn 2p, Cl 2p, respectively.

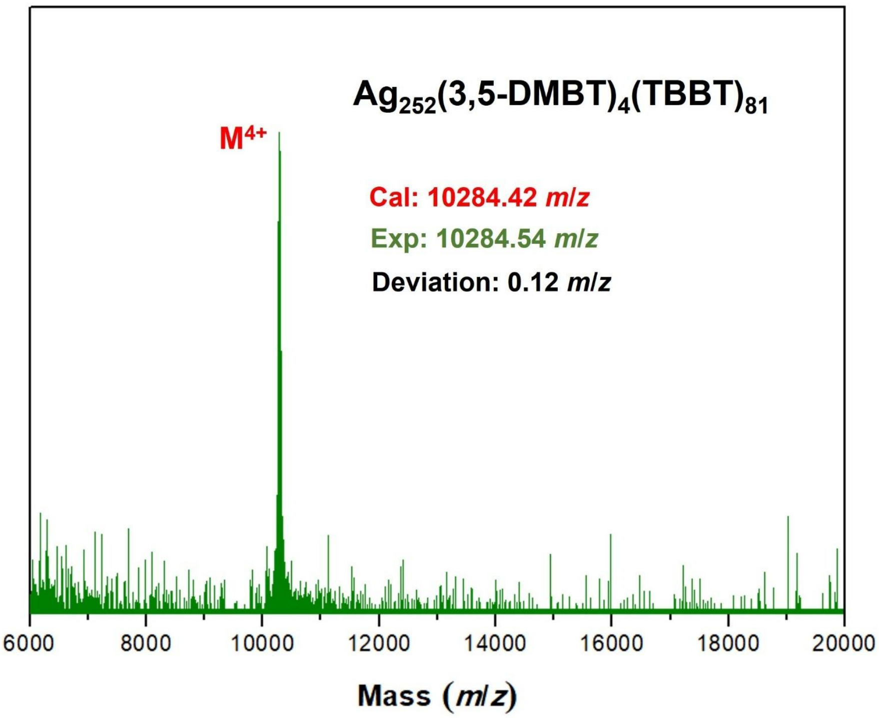

Figure S9. ESI-MS spectrum of Ag_{252}. Positive-ion mode ESI-MS of as-synthesized Ag_{252} dissolved in toluene and dichloromethane in a volume ratio of 3:1 (toluene *vs* dichloromethane).

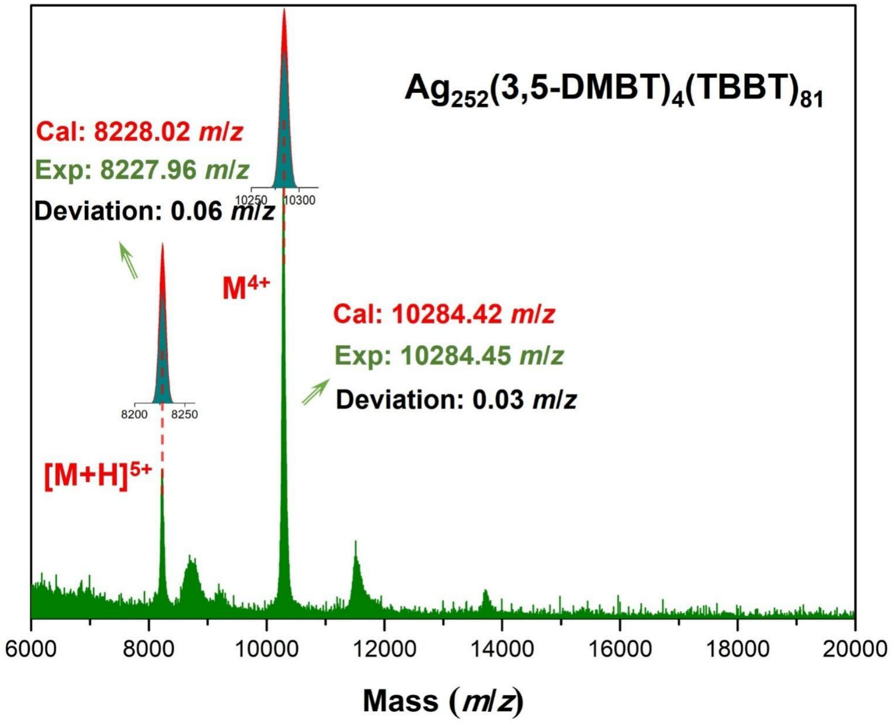

Figure S10. ESI-MS spectrum of Ag_{252}. Positive-ion mode ESI-MS of as-synthesized Ag_{252} dissolved in toluene and dichloromethane in a volume ratio of 3:1 (toluene *vs* dichloromethane) with the addition of cesium acetate.

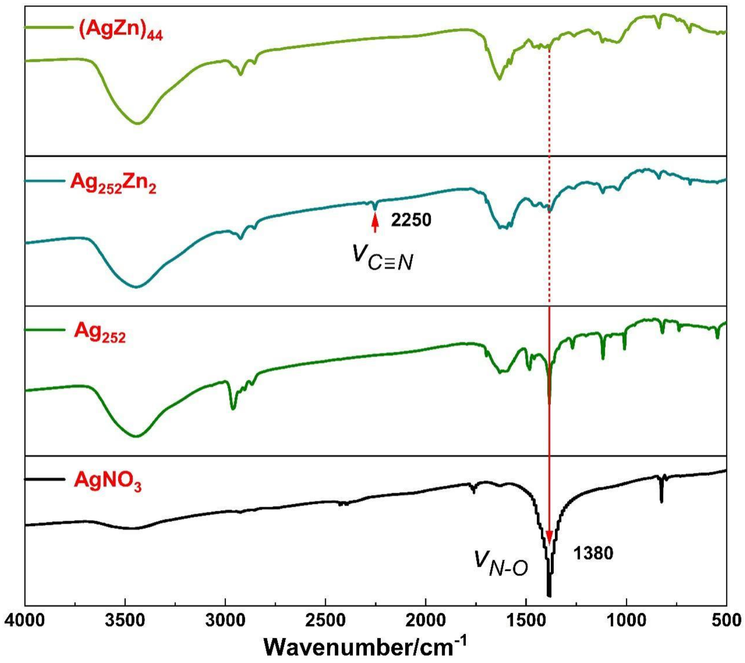

Figure S11. The IR spectra of $AgNO_3$, $(AgZn)_{44}$, $Ag_{252}Zn_2$, and Ag_{252}.

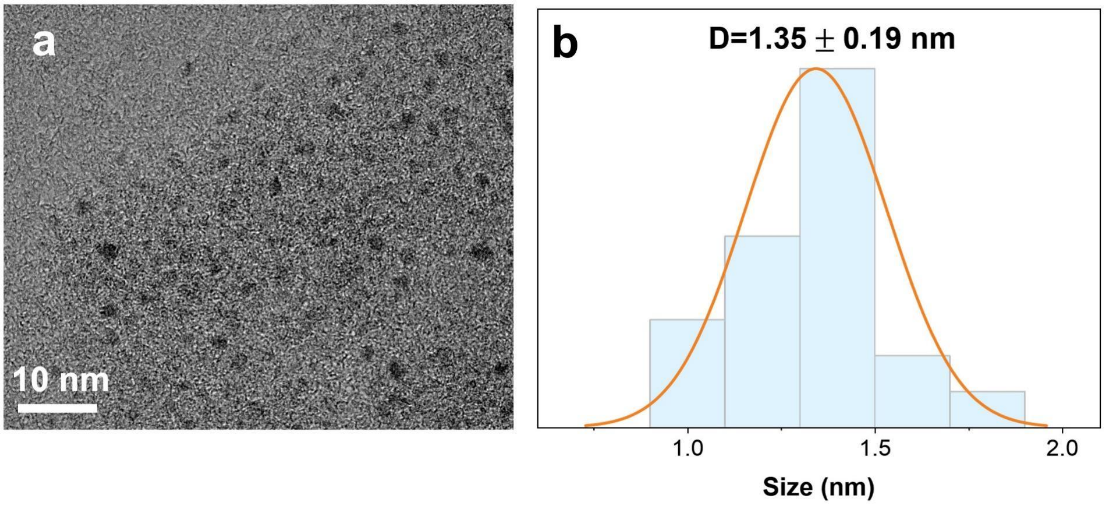

Figure S12. Particle size and distribution of $(AgZn)_{44}$. (a) TEM image of $(AgZn)_{44}$ nanoclusters (dispersed in DCM) and (b) the distribution of particle sizes.

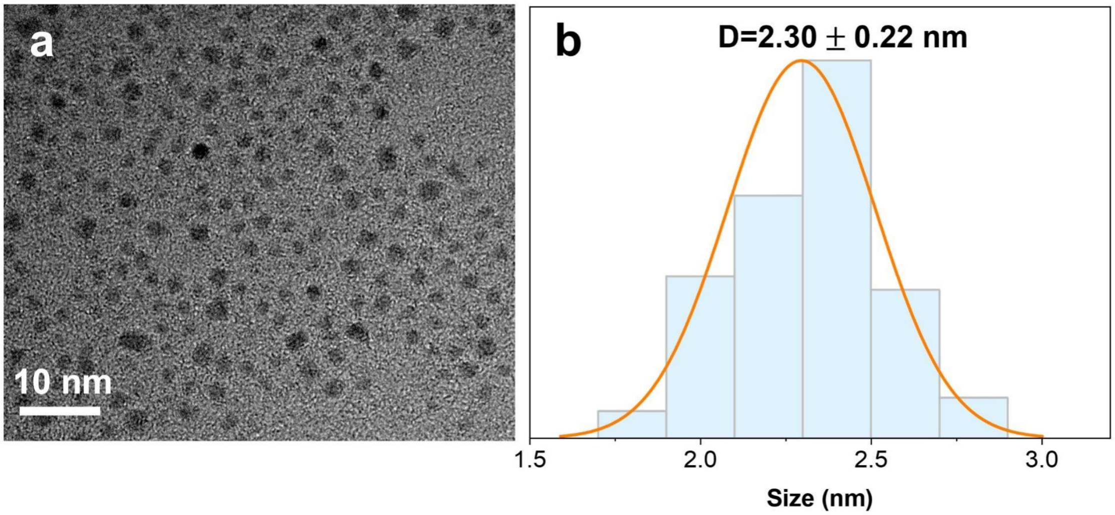

Figure S13. Particle size and distribution of $Ag_{252}Zn_2$. (a) TEM image of $Ag_{252}Zn_2$ nanoclusters (dispersed in DCM) and (b) the distribution of particle sizes.

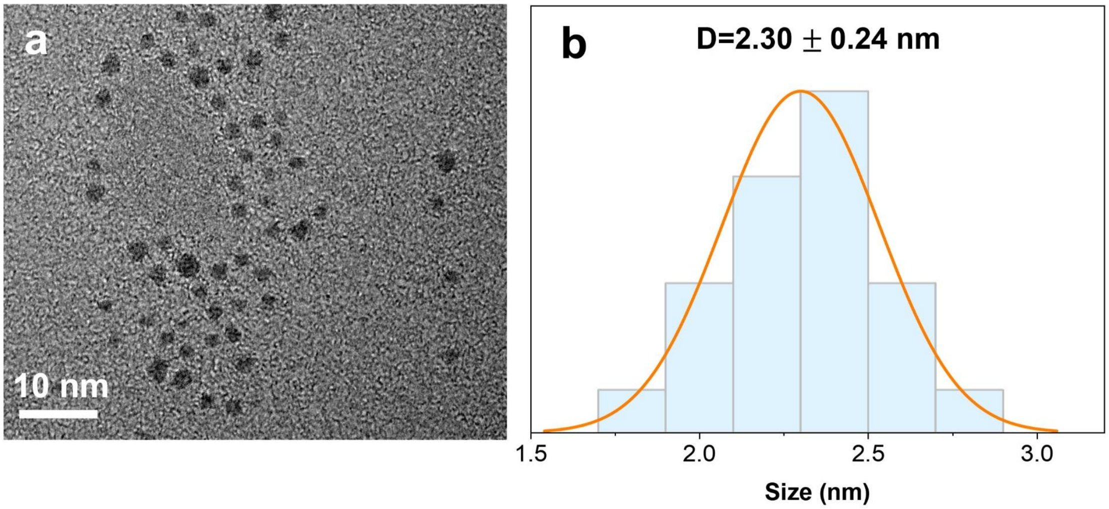

Figure S14. Particle size and distribution of Ag_{252}. (a) TEM image of Ag_{252} nanoclusters (dispersed in DCM) and (b) the distribution of particle sizes.

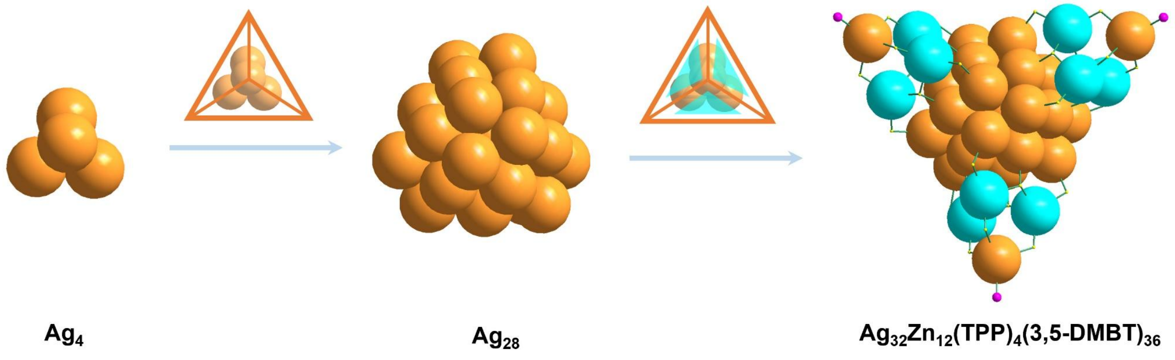

Figure S15. Kernel growth pattern of $Ag_{32}Zn_{12}$ from the inside to outside. Color labels: gold, Ag; green, Zn; yellow, S; pink, P.

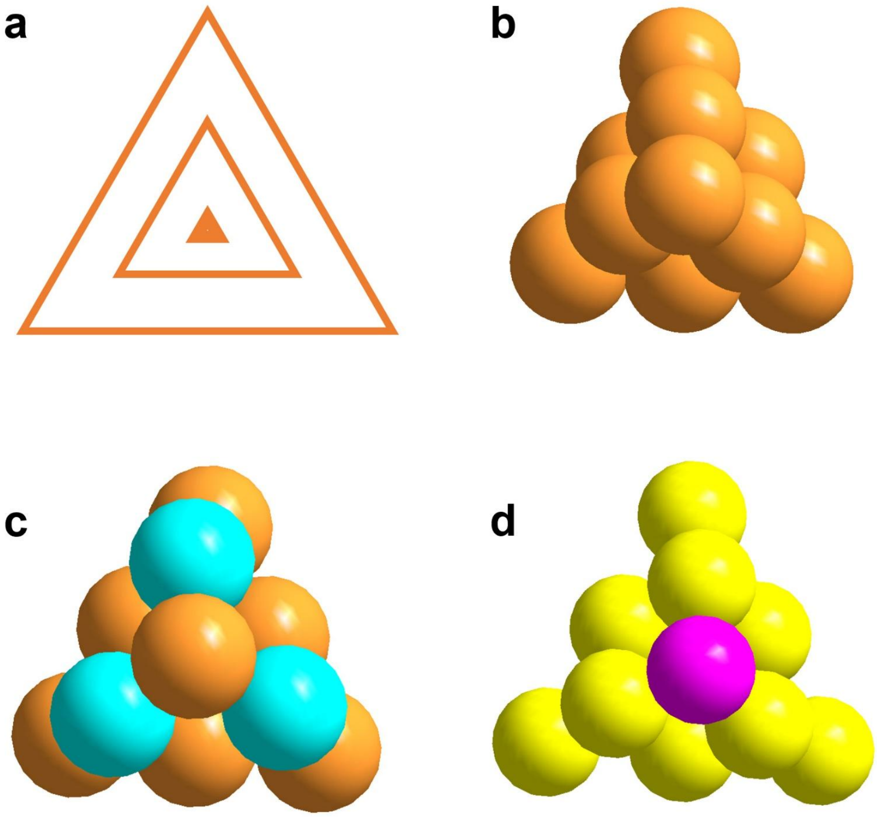

Figure S16. (a−d) The triangular numbers structures composed of different atoms. Color labels: gold, Ag; cyan, Zn; yellow, S; pink, P.

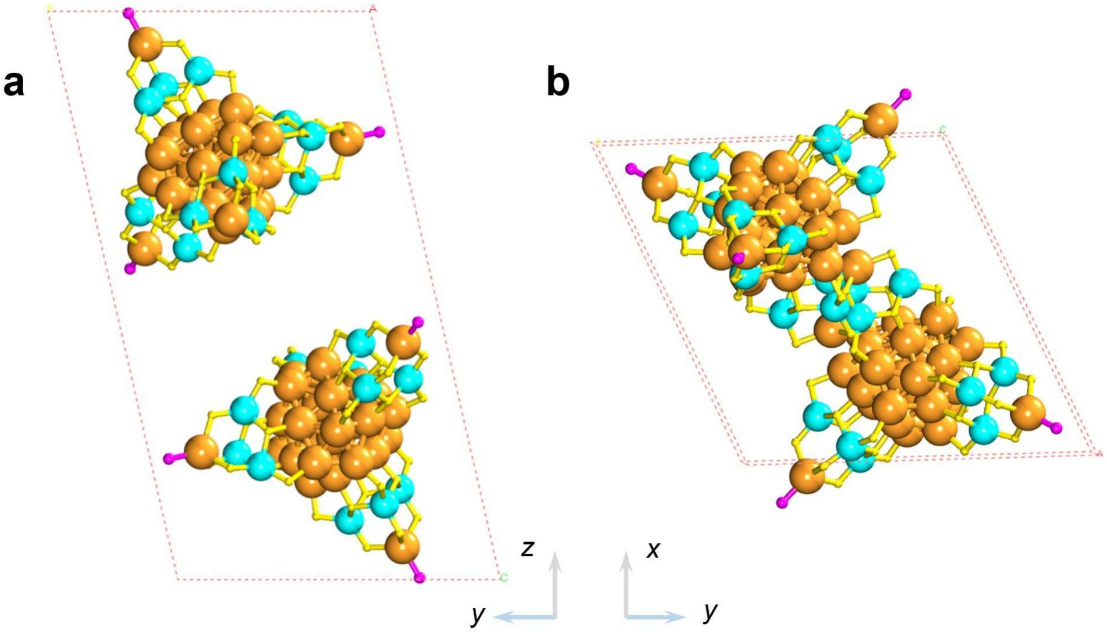

Figure S17. Crystal structure of $(AgZn)_{44}$. Crystal close-packing diagrams of $(AgZn)_{44}$ viewed along (a) *x*-axis and (b) *z*-axis. Color labels: gold, Ag; cyan, Zn; yellow, S; pink, P.

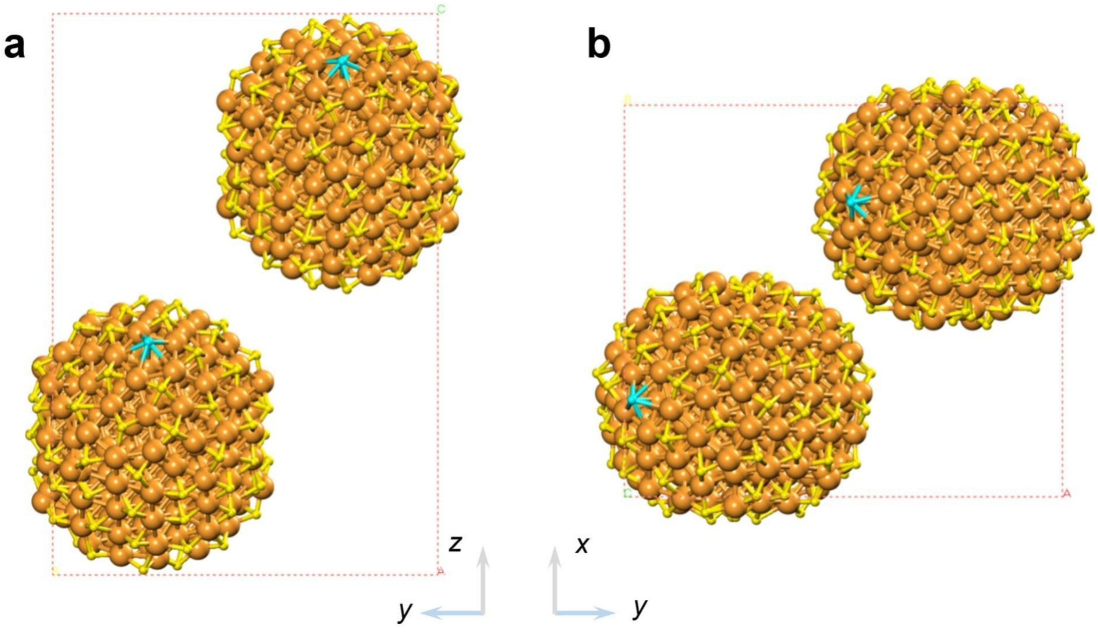

Figure S18. Crystal structure of $Ag_{252}Zn_2$. Crystal close-packing diagrams of $Ag_{252}Zn_2$ viewed along (a) *x*-axis and (b) *z*-axis axes. Color labels: gold, Ag; light green, Zn; yellow, S; pink, P.

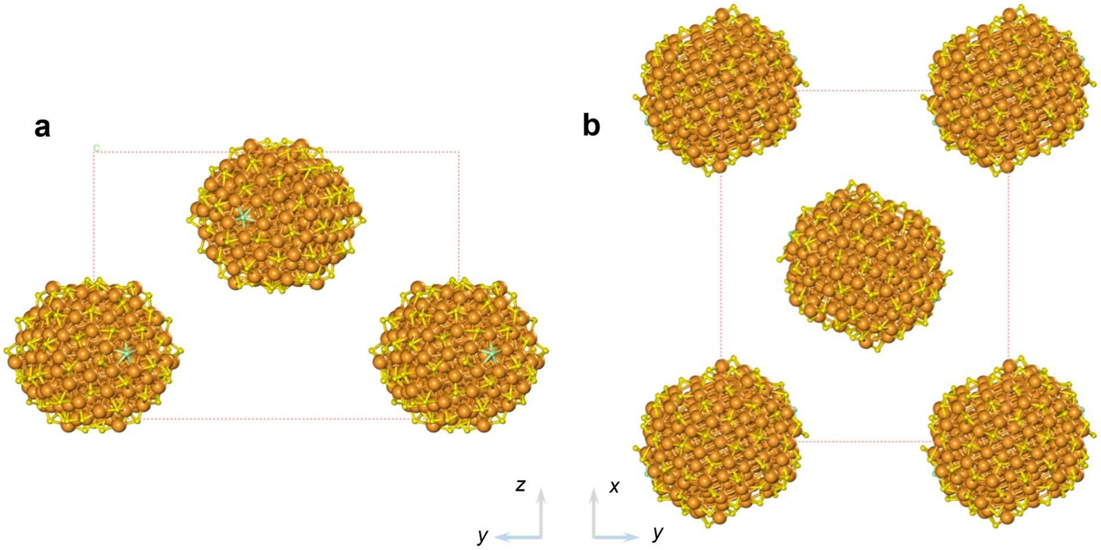

Figure S19. Crystal structure of Ag_{252}. Crystal close-packing diagrams of Ag_{252} viewed along (a) *x*-axis and (b) *z*-axis axes. Color labels: gold, Ag; light green, Zn; yellow, S.

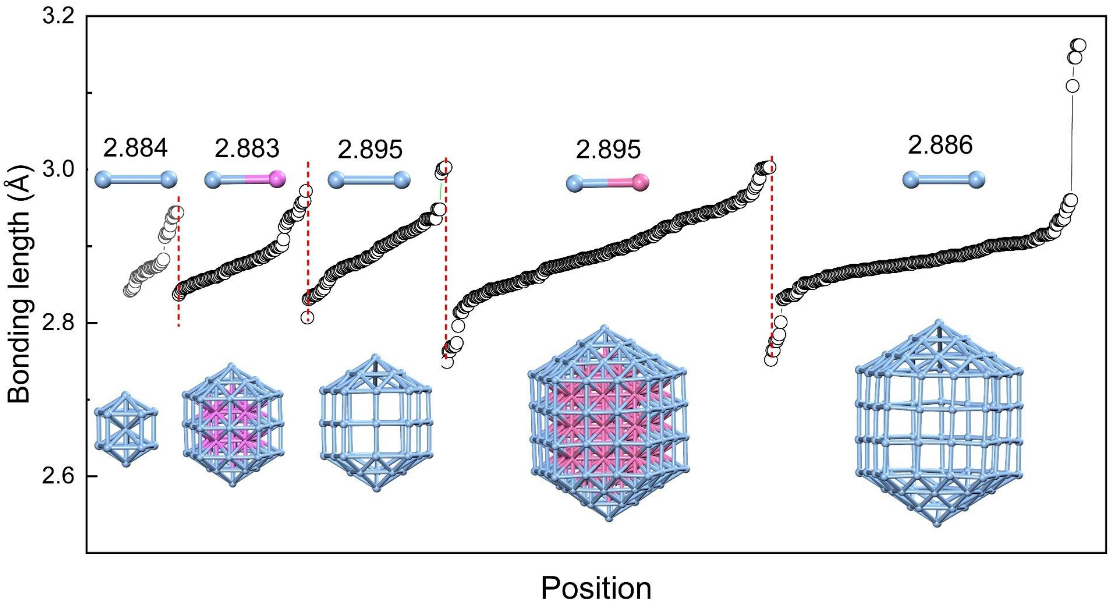

Figure S20. Ag–Ag bond length distributions in the metal kernel of the $Ag_{252}Zn_2$ nanoparticle. Color labels: light blue/pink, Ag.

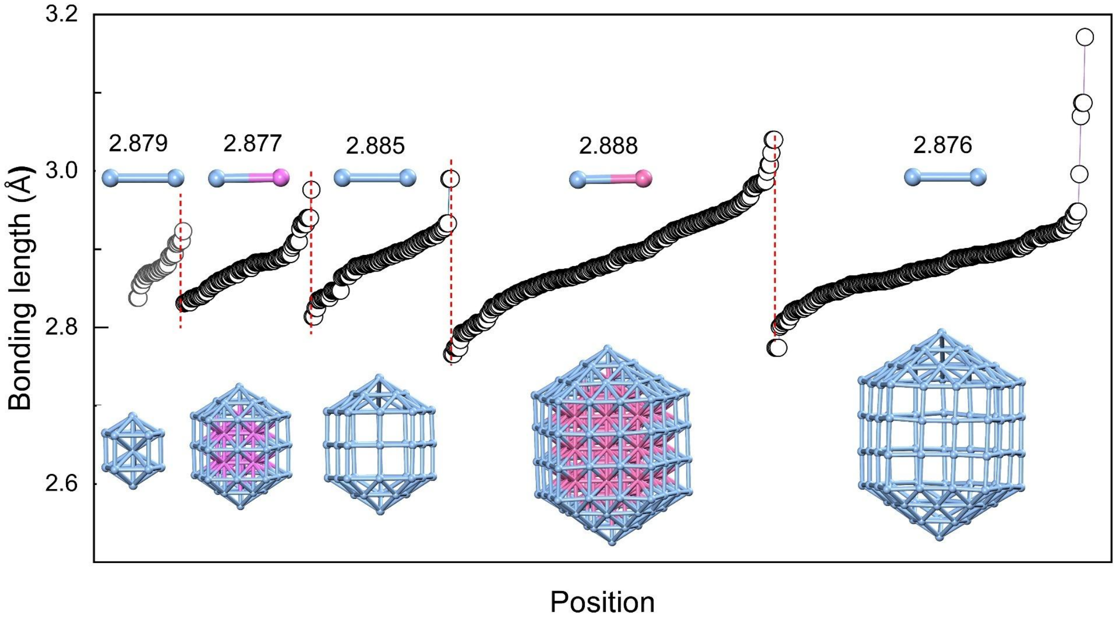

Figure S21. Ag–Ag bond length distributions in the metal kernel of the Ag_{252} nanoparticle. Color labels: light blue/pink, Ag.

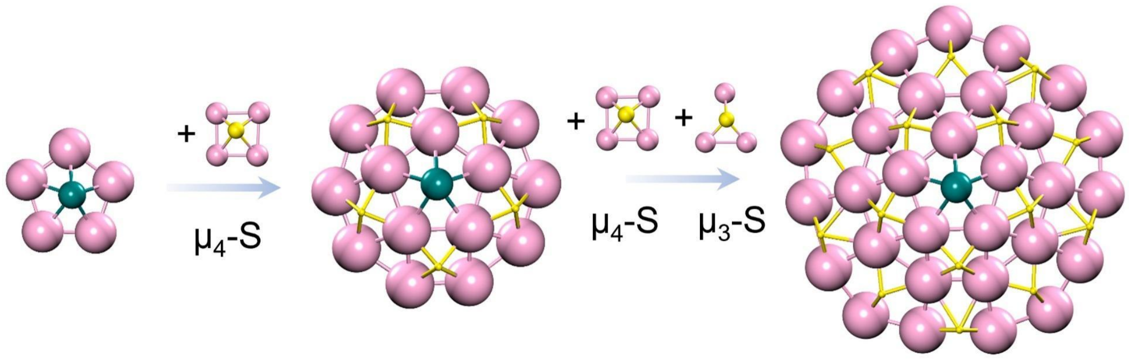

Figure S22. Top view of the apical staple structure dissection. Color labels: pink, Ag; yellow, S; dark green, Zn or Cl; C, H, and N are omitted.

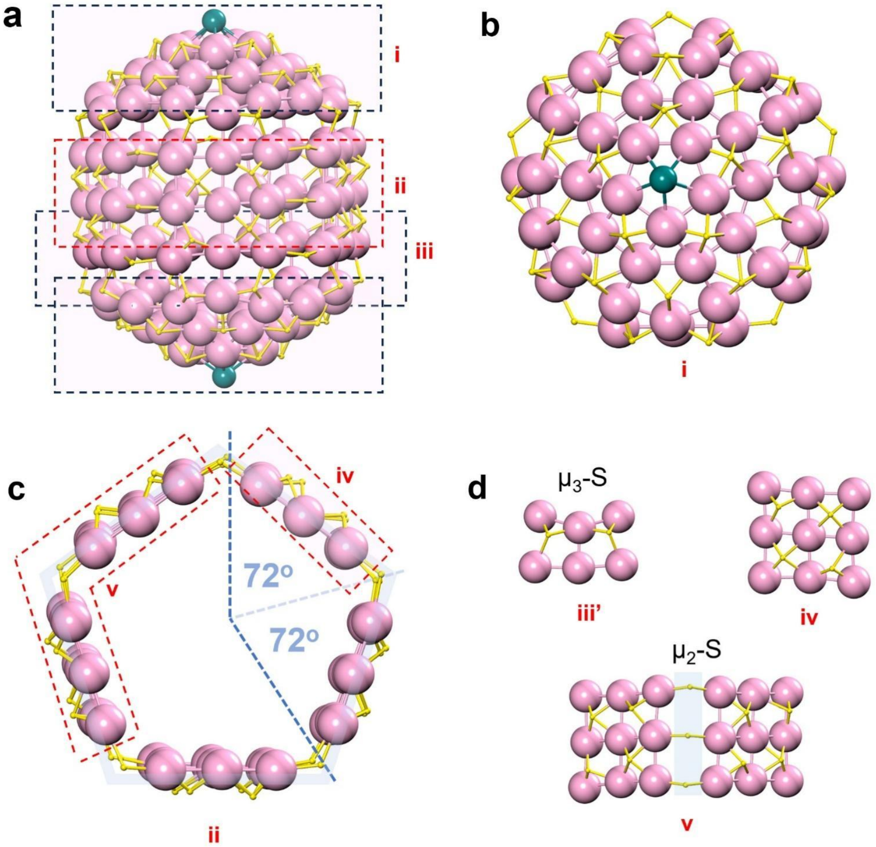

Figure S23. Staple structures. (a) Side view summary of staple structures; (b) top view of apical staples in unit i of (a); (c) top view of side staples in unit ii of (a); (d) Connection structure (unit iii', the minimum symmetrical structure of unit iii) linking apical and side staples, along with the minimum symmetrical structure (unit iv) and connection structure unit v. Color labels: pink, Ag; yellow, S; dark green, Zn or Cl; C, H and N are omitted.

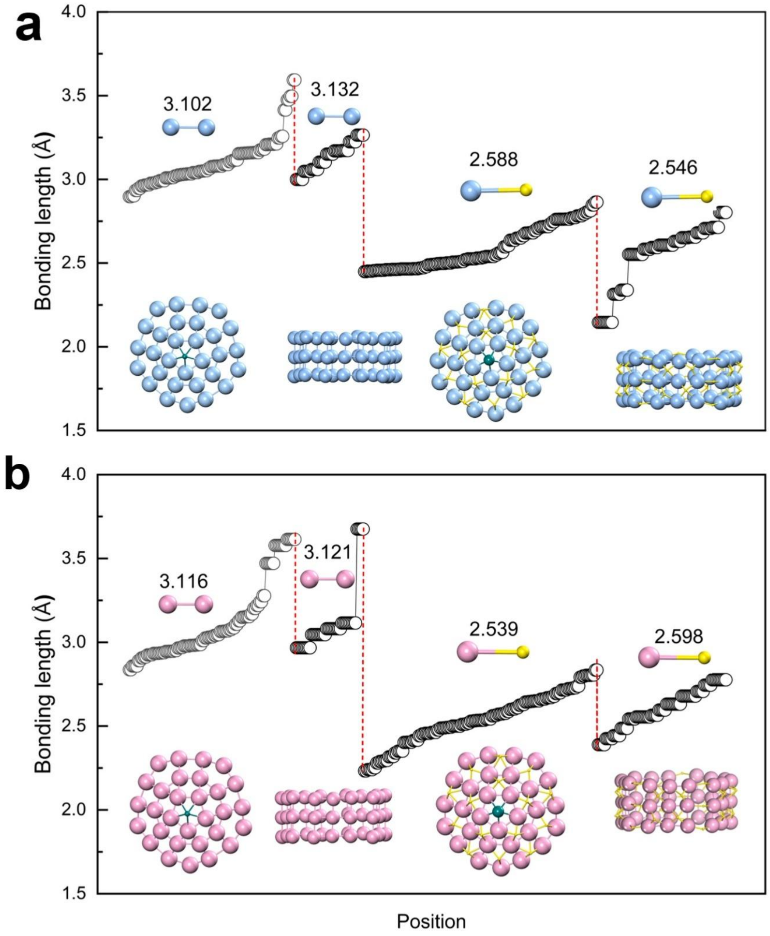

Figure S24. Comparison of bond length. Ag–Ag and Ag–S bond length distributions in (a) $Ag_{252}Zn_2$ and (b) $Ag_{252}Zn_2$ nanoparticles. Color labels: light blue/pink, Ag; orange, S; blue, Zn and Cl.

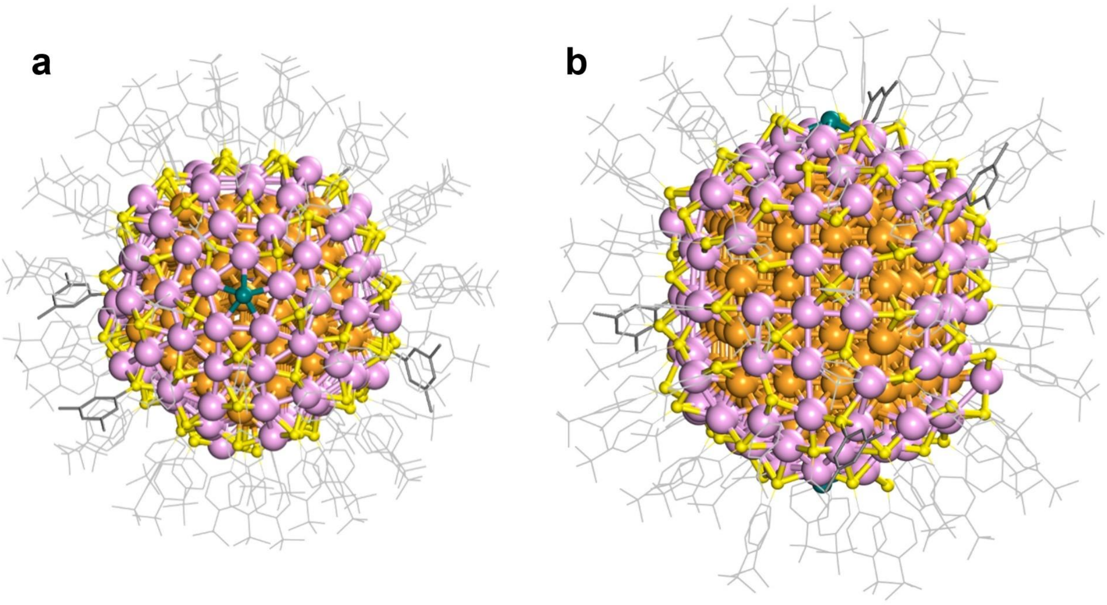

Figure S25. Crystal structure of Ag_{252}. (a) Top and (b) side views of the Ag_{252} structure. Color labels: gold and pink, Ag; yellow, S; dark green, Cl; grey, C and H.

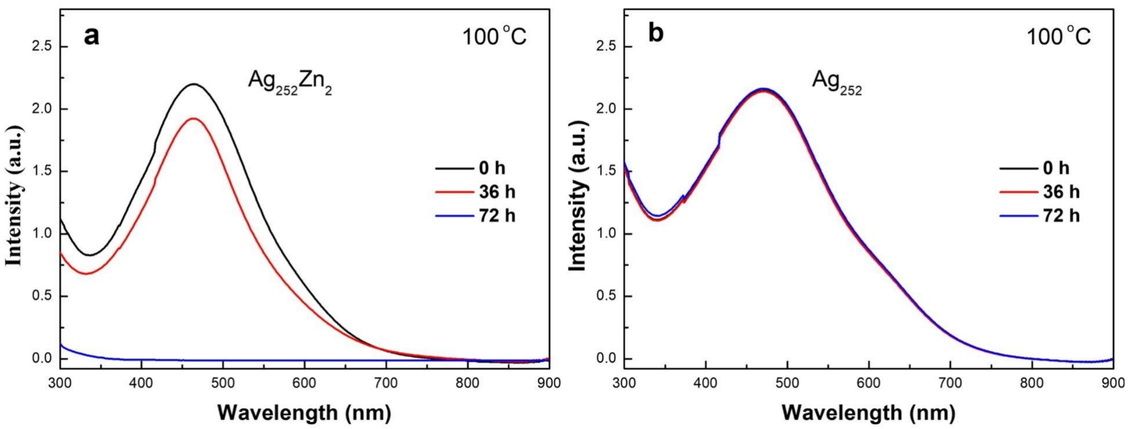

Figure S26. Comparison of thermal stability. Time-dependent UV–vis–NIR absorption spectra of (a) $Ag_{252}Zn_2$ and (b) Ag_{252} under 100°C. Concentration: ~0.003 mg/mL; solvent: toluene.

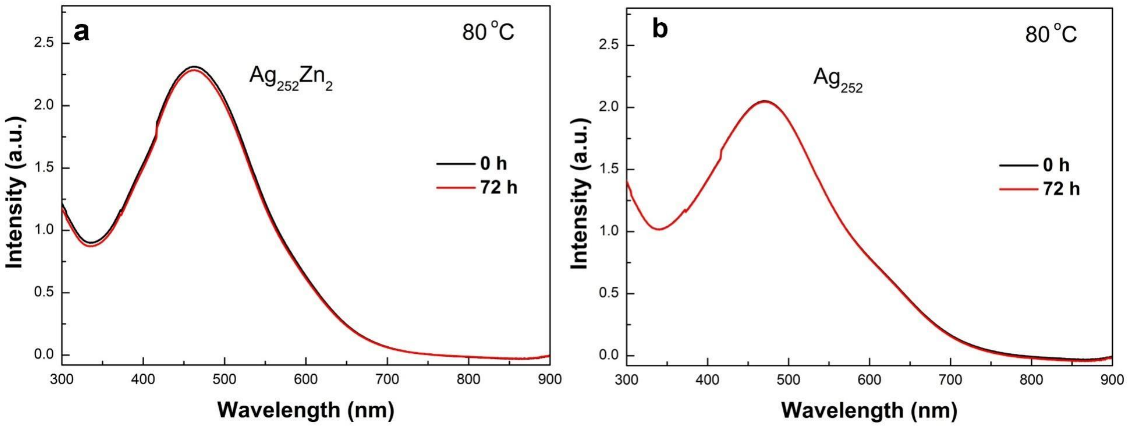

Figure S27. Comparison of thermal stability. Time-dependent UV–vis–NIR absorption spectra of (a) $Ag_{252}Zn_2$ and (b) Ag_{252} under 80°C. Concentration: ~0.003 mg/mL; solvent: toluene.

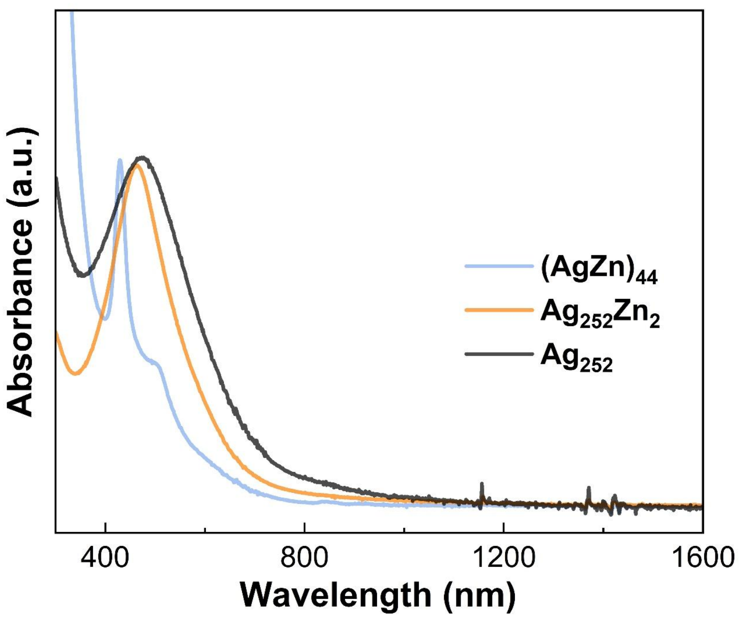

Figure S28. UV-Vis-NIR absorption spectra of $(AgZn)_{44}$, $Ag_{252}Zn_2$ and Ag_{252}.

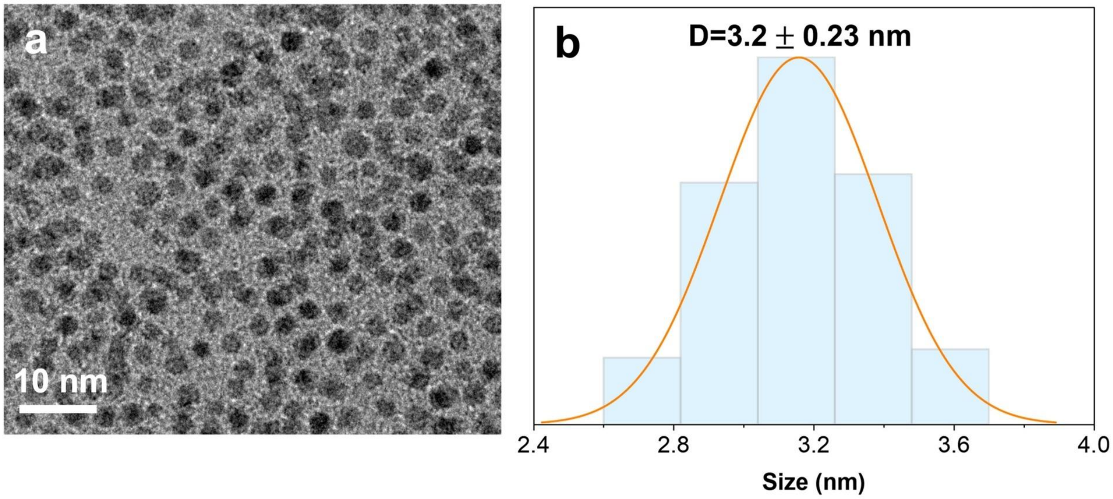

Figure S29. Particle size and distribution of ~3.2 nm Ag nanocrystals. (a) TEM image of ~3.2 nm Ag nanocrystals (dispersed in DCM) and (b) the distribution of particle sizes.

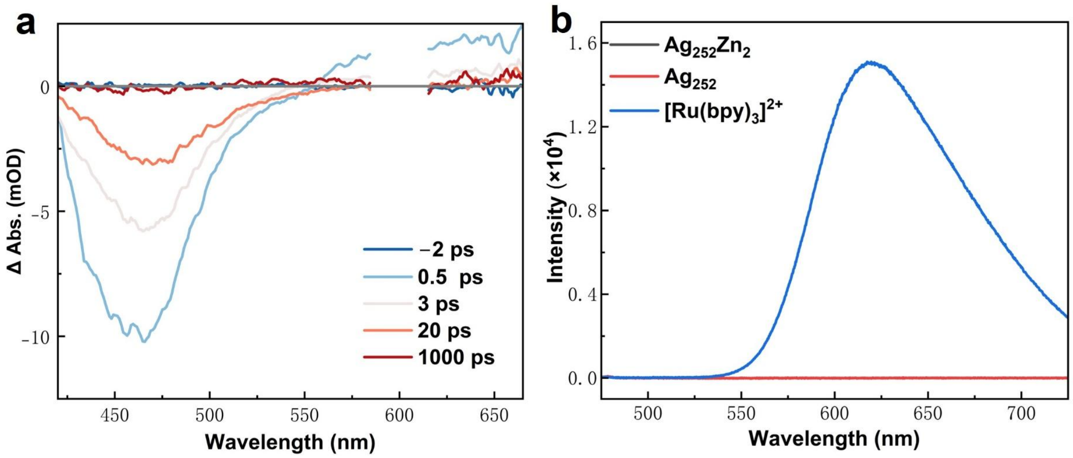

Figure S30. Absorption difference spectra and emission spectra. (a) Absorption difference spectra of $Ag_{252}Zn_2$ following 600 nm pulsed laser excitation at several representative time delays. (b) The emission spectra under 400 nm excitation; $[Ru(bpy)_3]^{2+}$ is investigated for comparison.

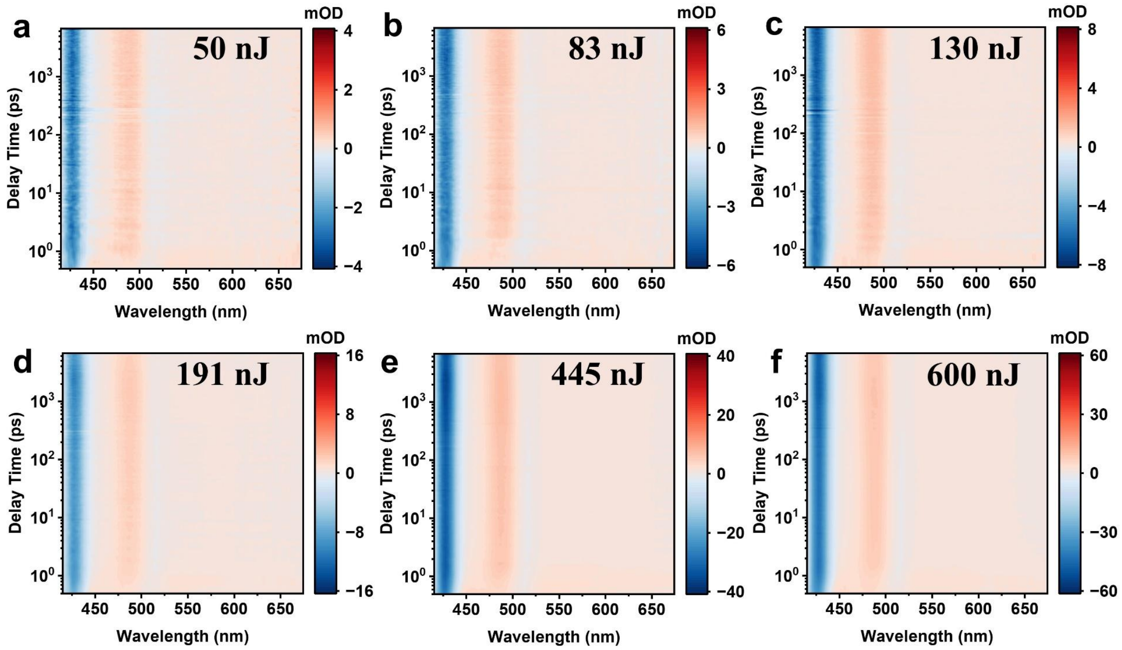

Figure S31. TA data maps. (a−f) TA maps of $(AgZn)_{44}$ under 400 nm excitation with various pump powers.

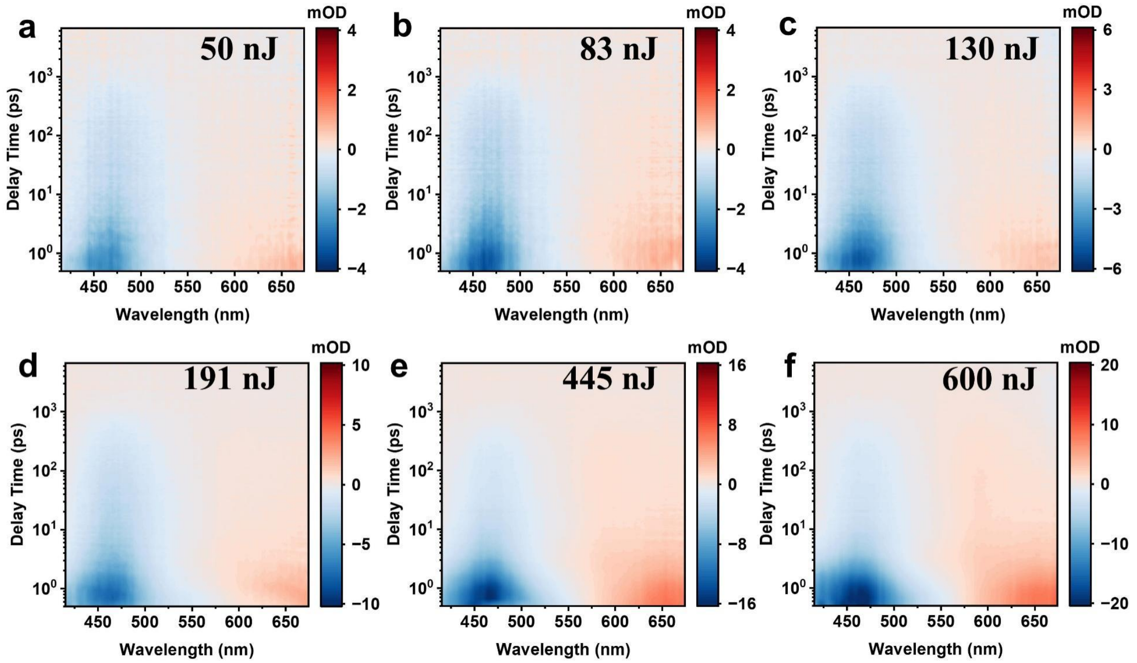

Figure S32. TA data maps. (a−f) TA maps of $Ag_{252}Zn_2$ under 400 nm excitation with various pump powers.

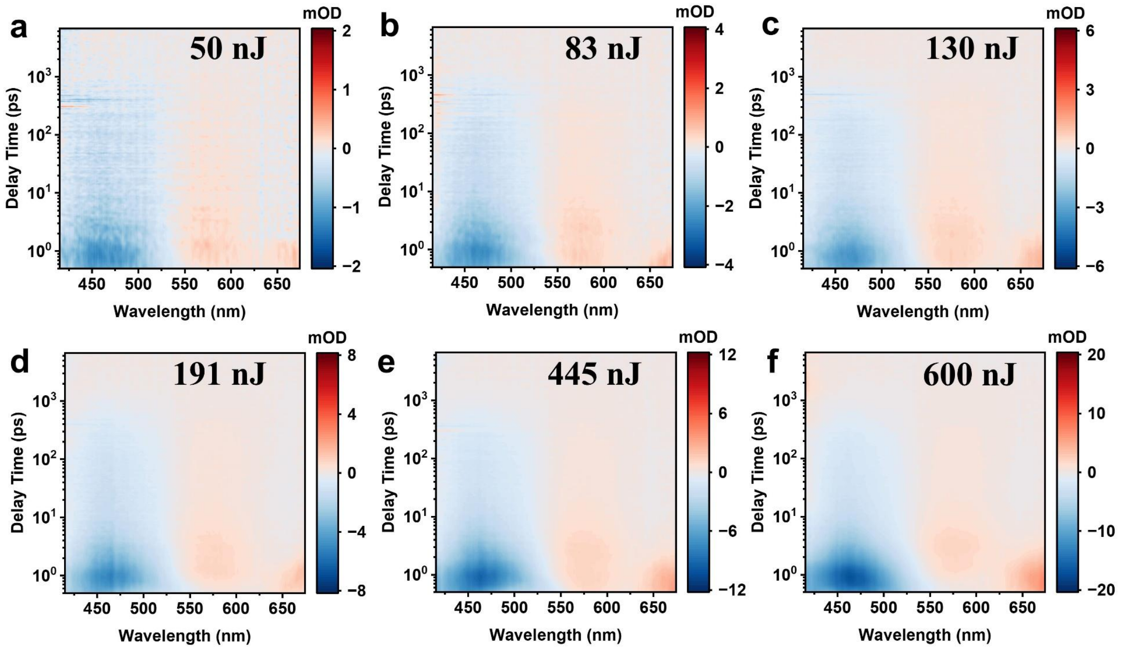

Figure S33. TA data maps. (a−f) TA maps of Ag_{252} under 400 nm excitation with various pump powers.

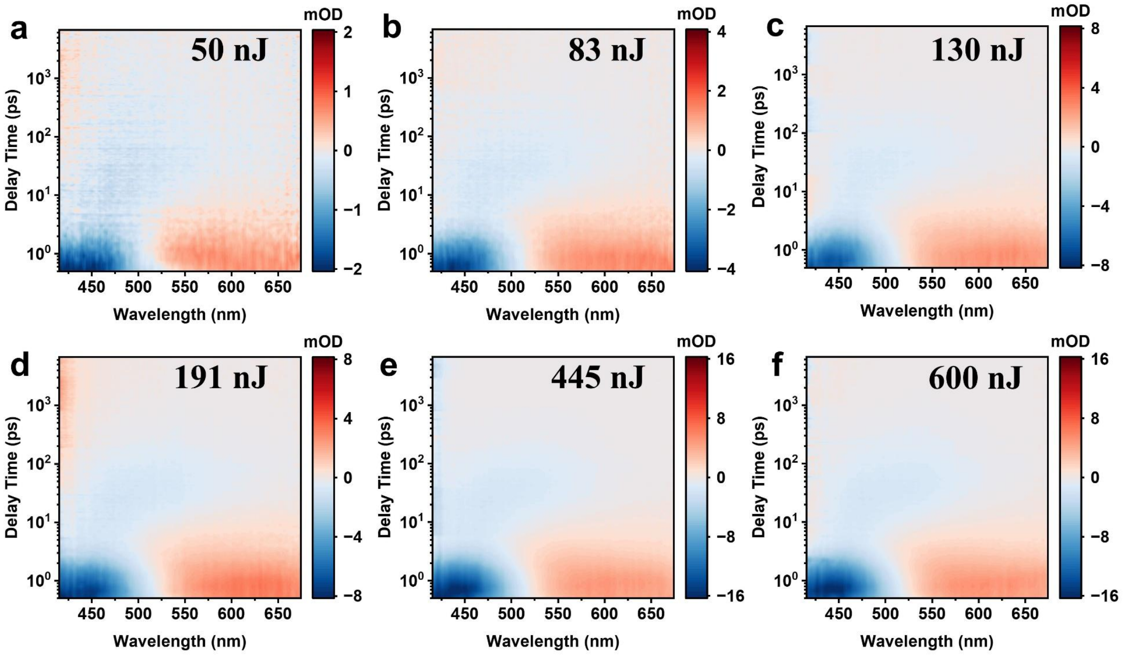

Figure S34. TA data maps. (a−f) TA maps of ~3.2 nm Ag nanocrystals under 400 nm excitation with various pump powers.

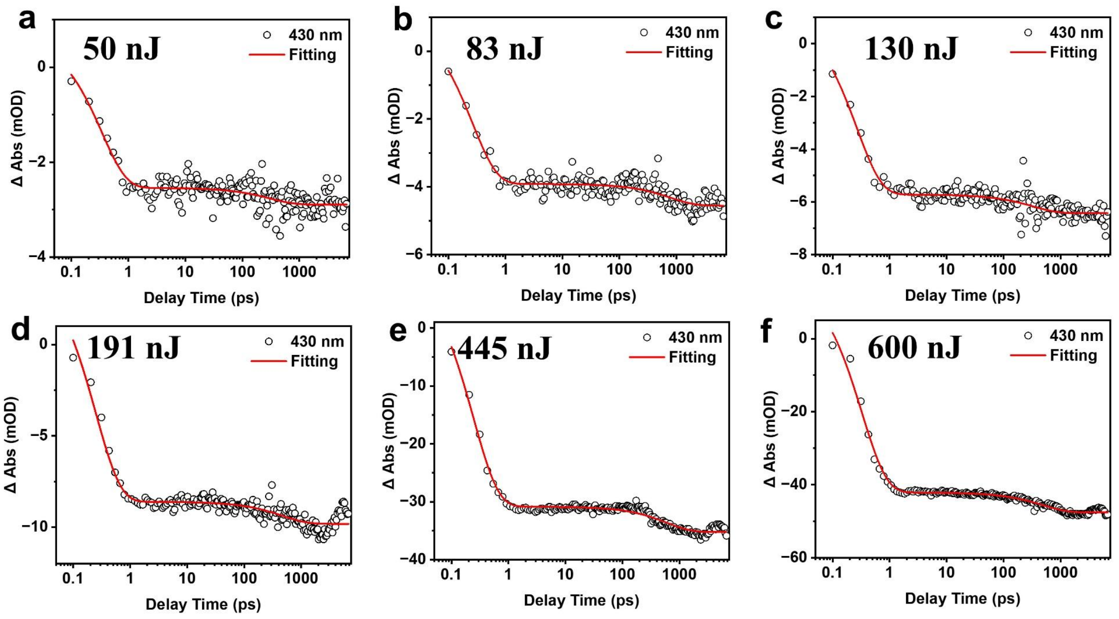

Figure S35. Kinetic curves. (a−f) Kinetic curves of $(AgZn)_{44}$ probed at 430 nm under 400 nm excitation.

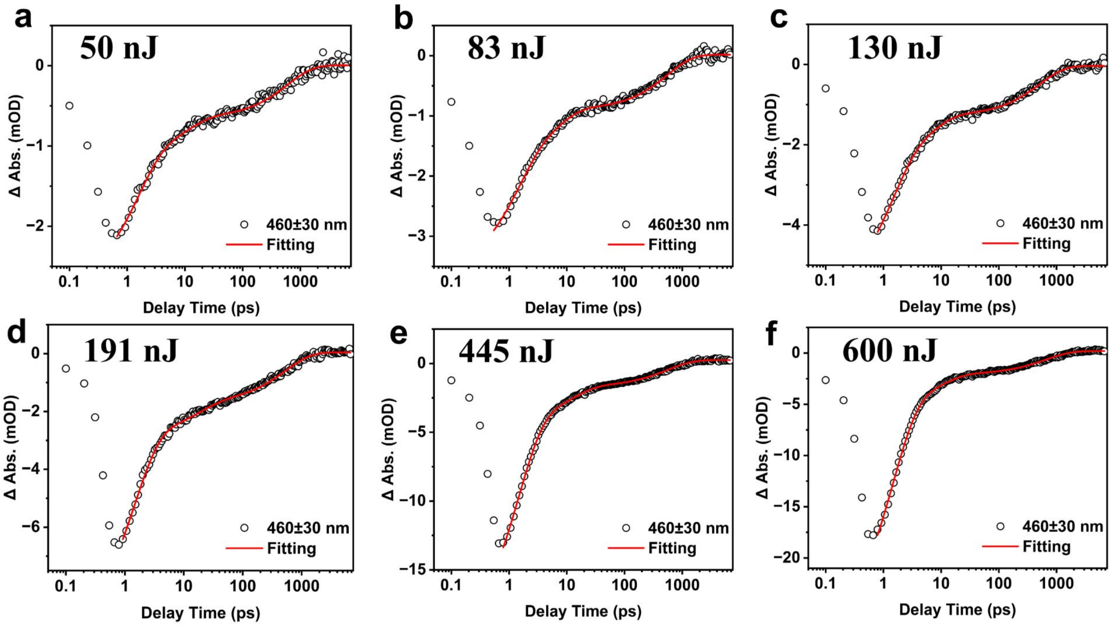

Figure S36. Kinetic curves. (a−f) Kinetic curves of $Ag_{252}Zn_2$ probed at 460 ± 30 nm under 400 nm excitation.

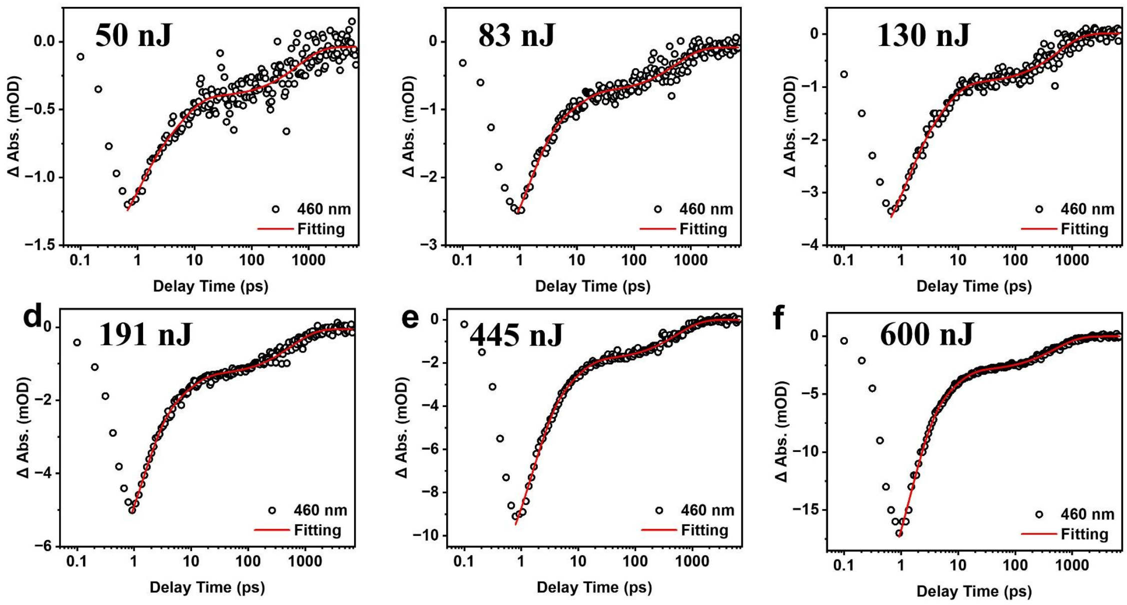

Figure S37. Kinetic curves. (a−f) Kinetic curves of Ag_{252} probed at 460 nm under 400 nm excitation.

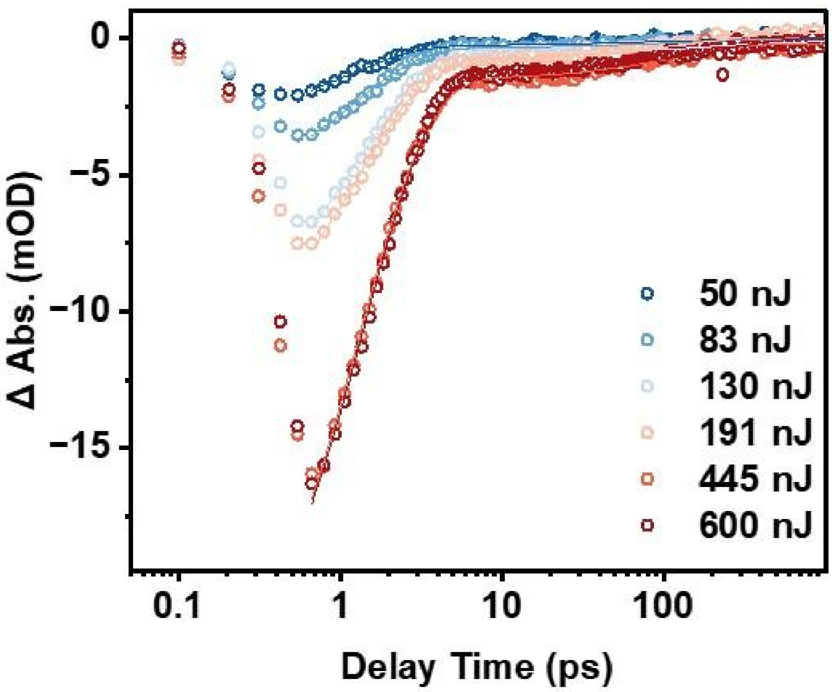

Figure S38. Kinetic curves of ~3.2 nm Ag nanocrystals probed at 450 nm under 400 nm excitation.

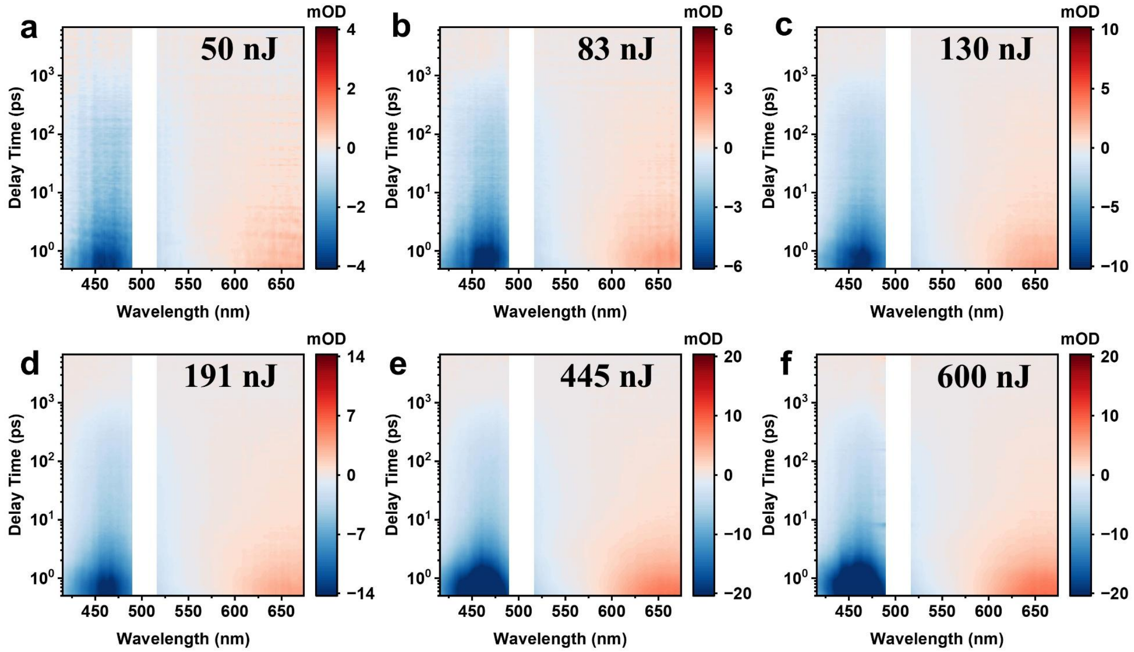

Figure S39. TA data maps. (a−f) TA maps of $Ag_{252}Zn_2$ under 500 nm excitation with various pump powers.

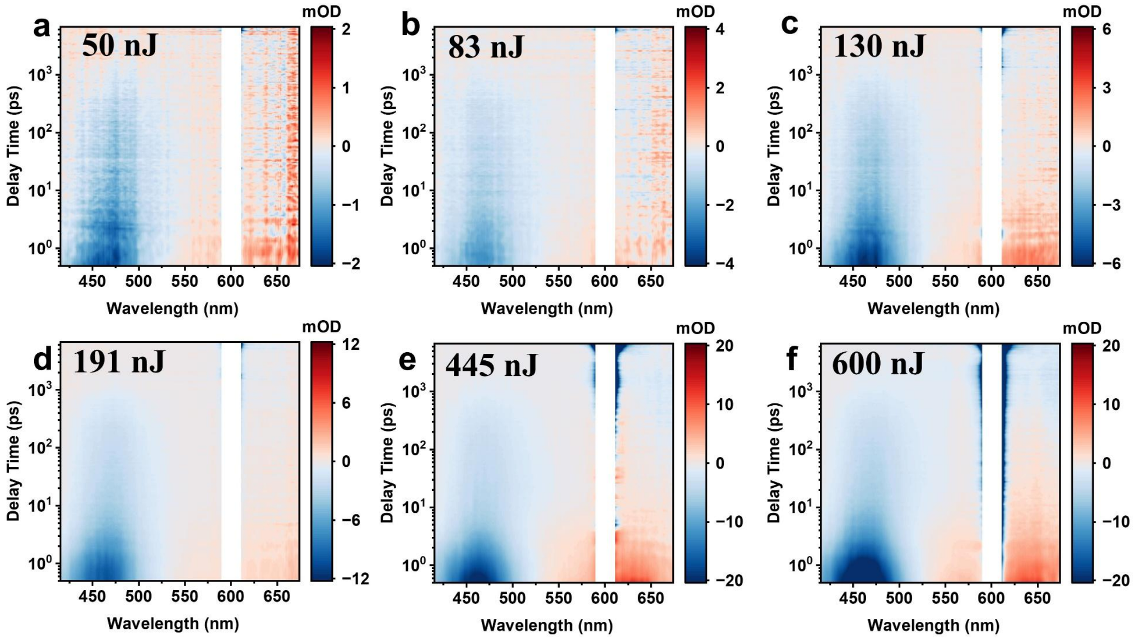

Figure S40. TA data maps. (a−f) TA maps of $Ag_{252}Zn_2$ under 600 nm excitation with various pump powers.

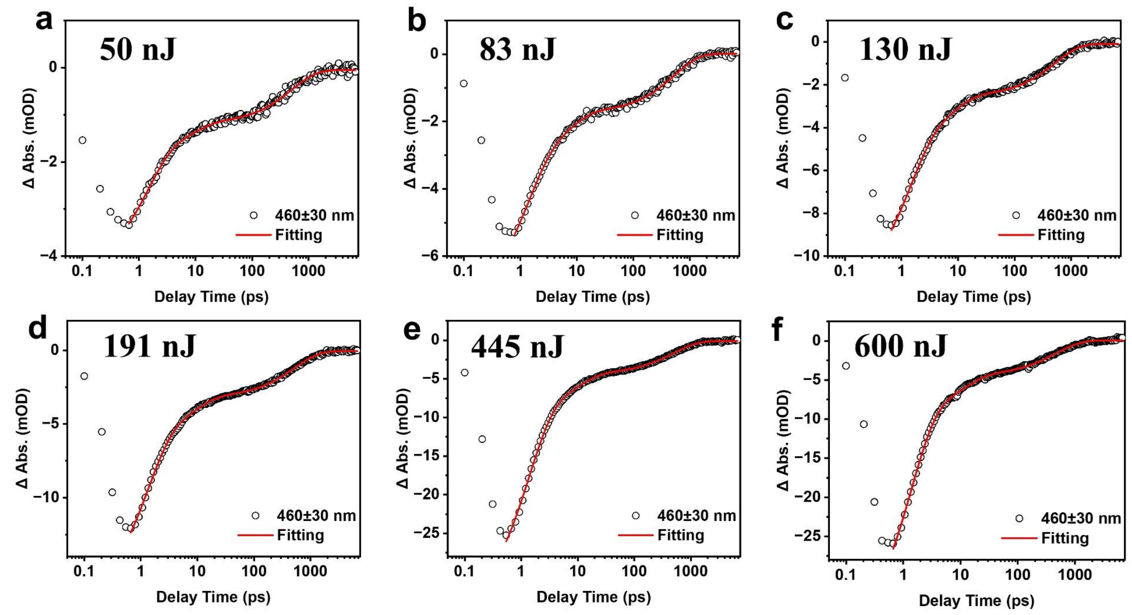

Figure S41. Kinetic curves. (a−f) Kinetic curves of $Ag_{252}Zn_2$ probed at 460 ± 30 nm under 500 nm excitation.

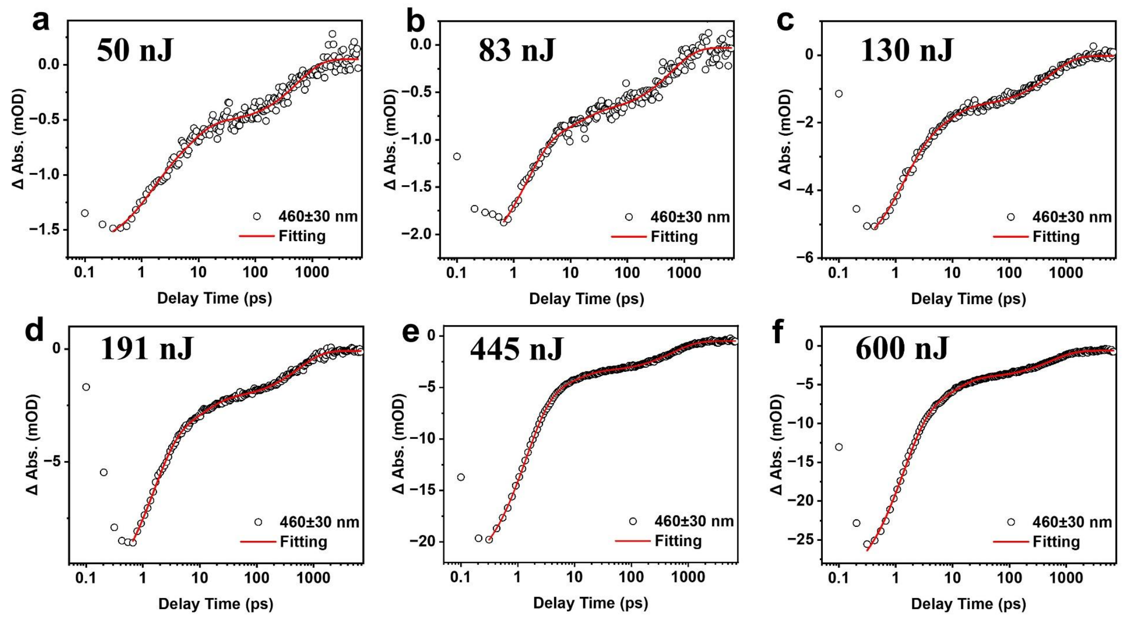

Figure S42. Kinetic curves. (a−f) Kinetic curves of $Ag_{252}Zn_2$ probed at 460 ± 30 nm under 600 nm excitation.

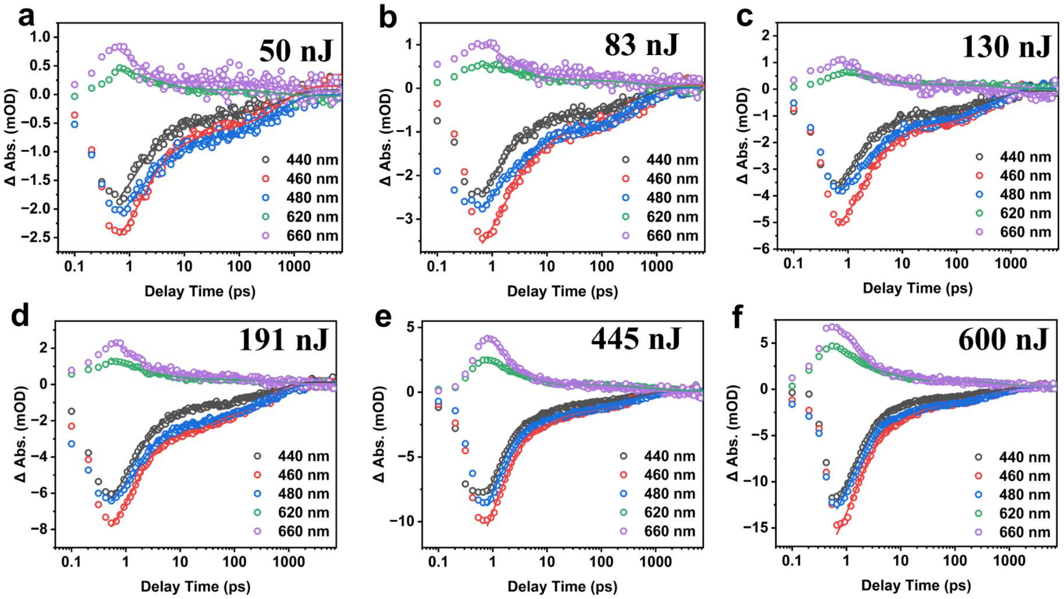

Figure S43. Kinetic curves. (a−f) Kinetic curves of $Ag_{252}Zn_2$ probed at different wavelengths under 400 nm excitation.

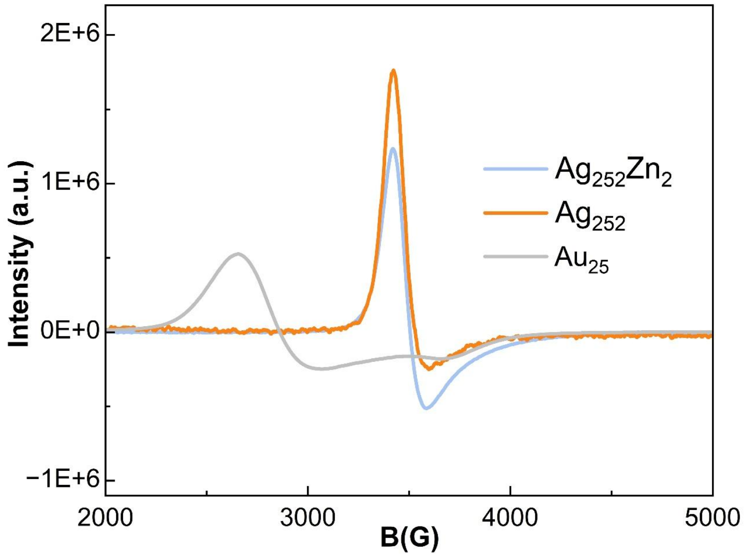

Figure S44. EPR measurements of $Ag_{252}Zn_2$ and Ag_{252} and Au_{25} (temperature: 2 K; amount: 0.11 μmol).

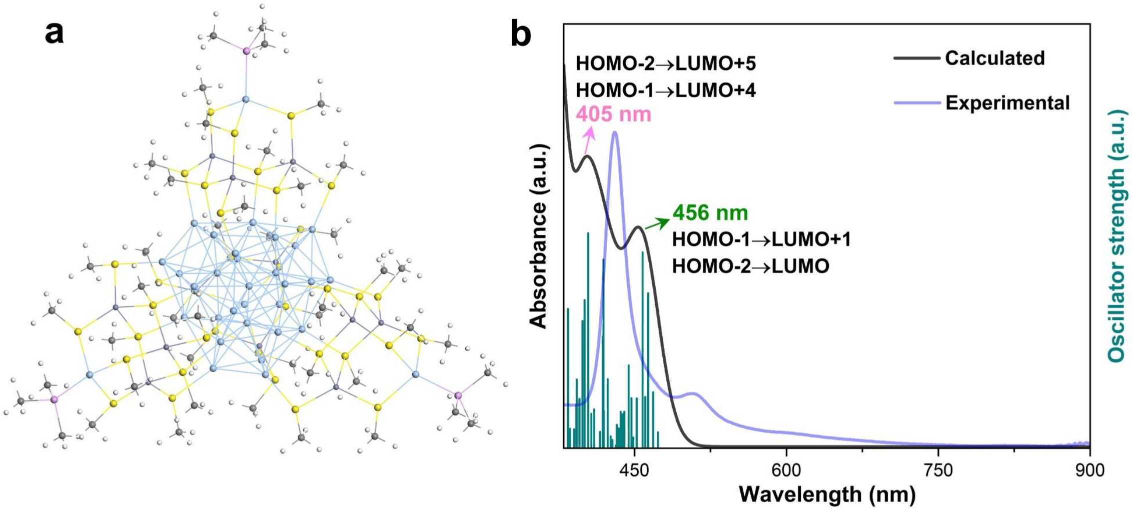

Figure S45. DFT calculations of $(AgZn)_{44}$. (a) The computational model of $(AgZn)_{44}$ after replacing all alkyl groups with R = CH_3; (b) The calculated UV–vis–NIR absorption spectrum of $(AgZn)_{44}$.

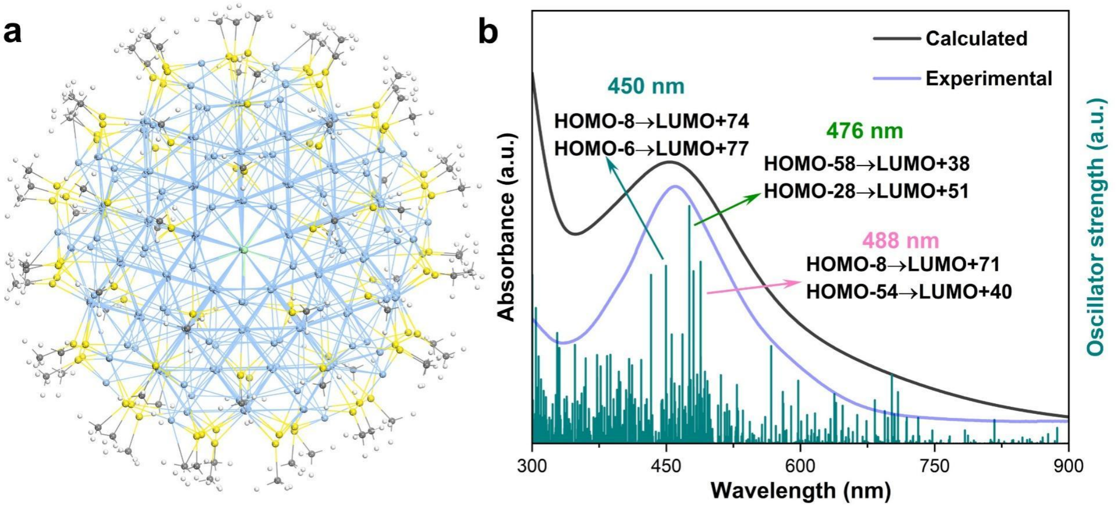

Figure S46. DFT calculations of Ag_{252}. (a) The computational model of Ag_{252} after replacing all alkyl groups with R = CH_3; (b) The calculated UV–vis–NIR absorption spectrum of Ag_{252}.

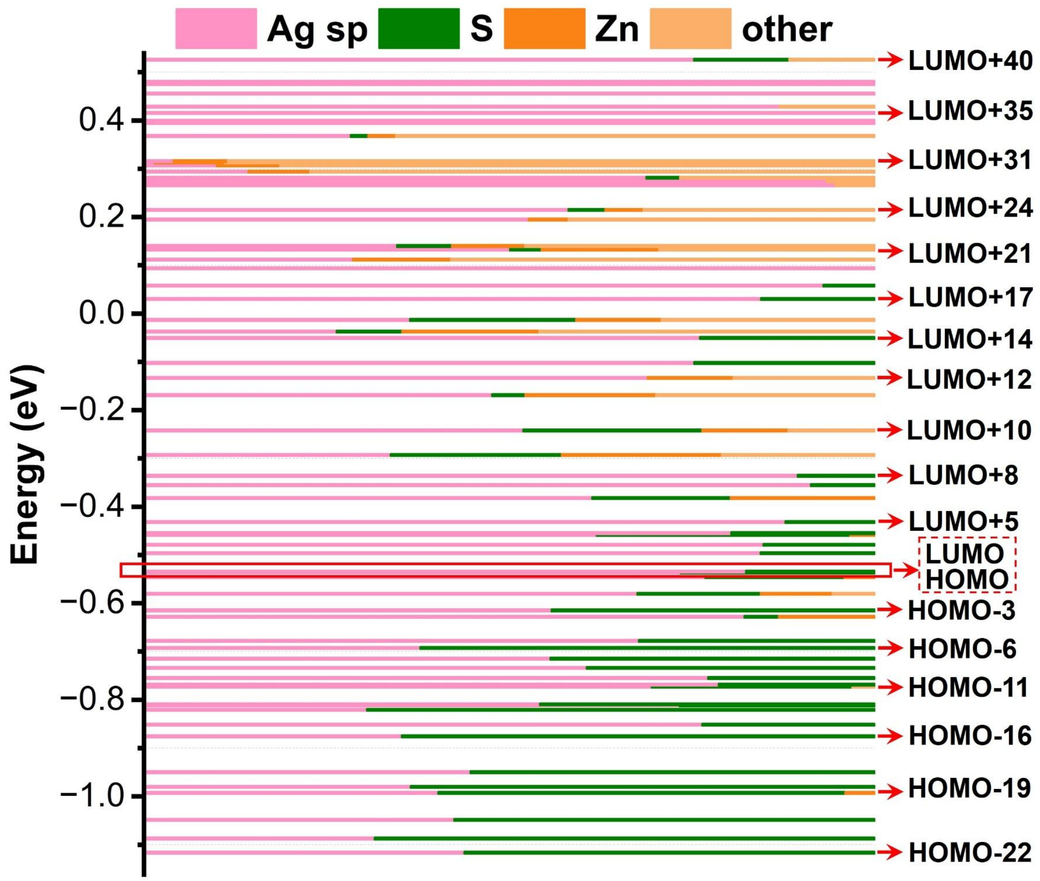

Figure S47. The distributions of $Ag_{252}Zn_2$ from HOMO-22 to LUMO+40.

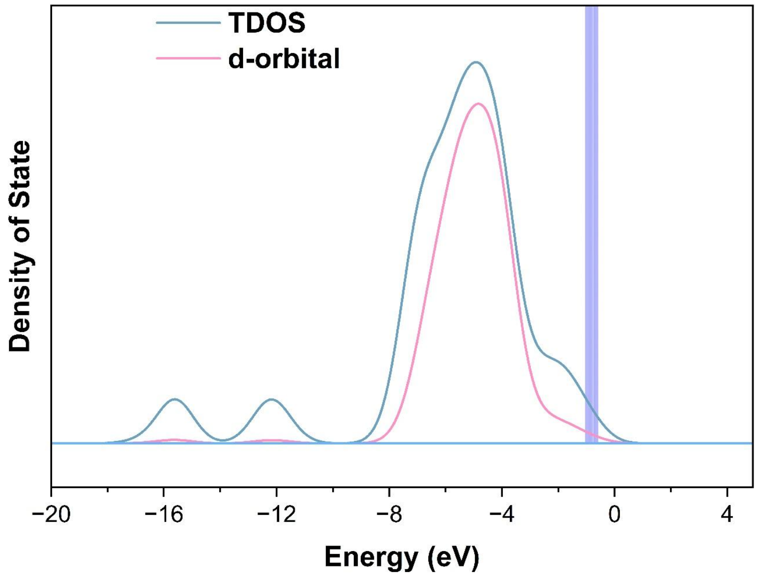

Figure S48. TDOS of $Ag_{252}Zn_2$. The light blue region denotes the orbitals that fall within the range from HOMO-20 to HOMO-4.

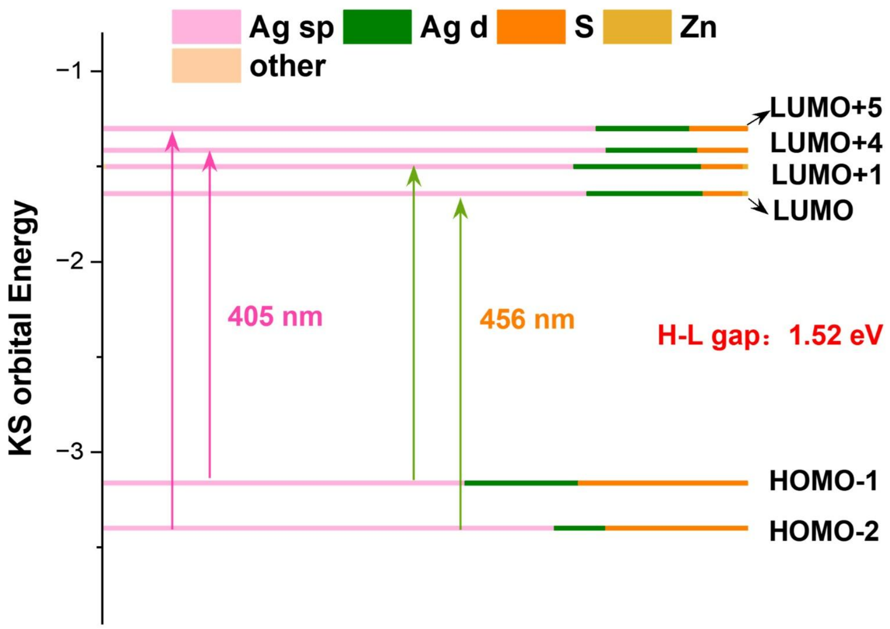

Figure S49. Kohn–Sham molecular energy level diagram of $(AgZn)_{44}$.

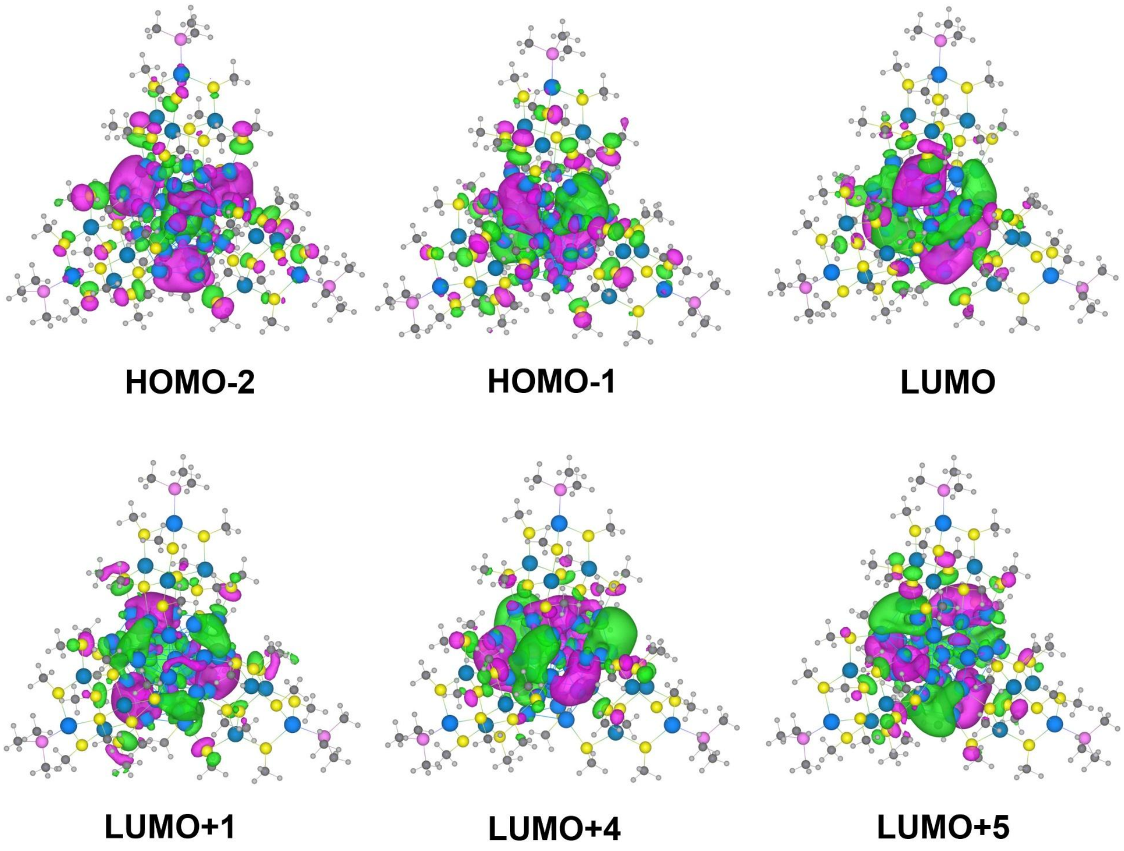

Figure S50. HOMO and LUMO distributions of $(AgZn)_{44}$.

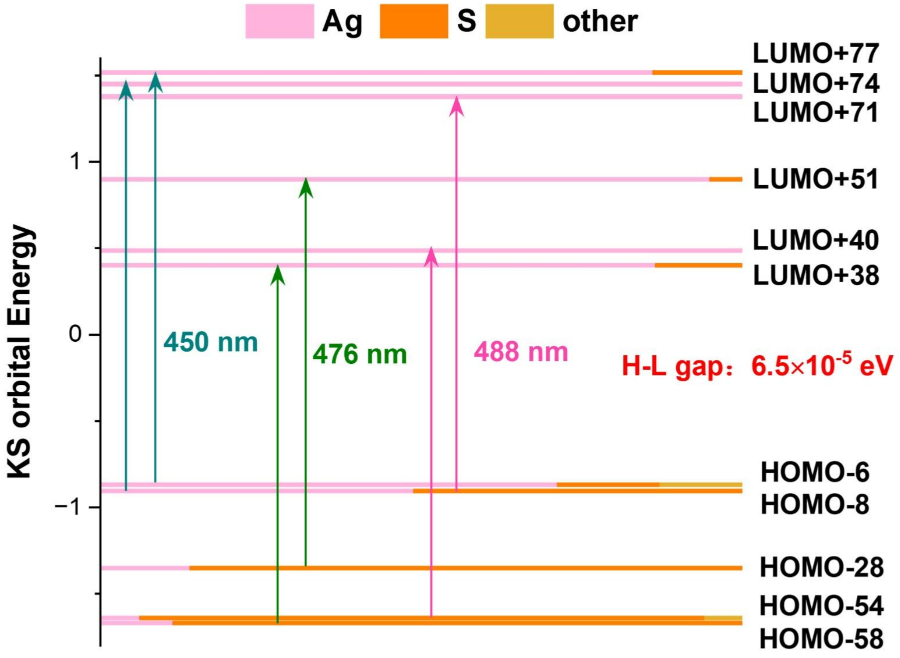

Figure S51. Kohn–Sham molecular energy level diagram of Ag_{252}.

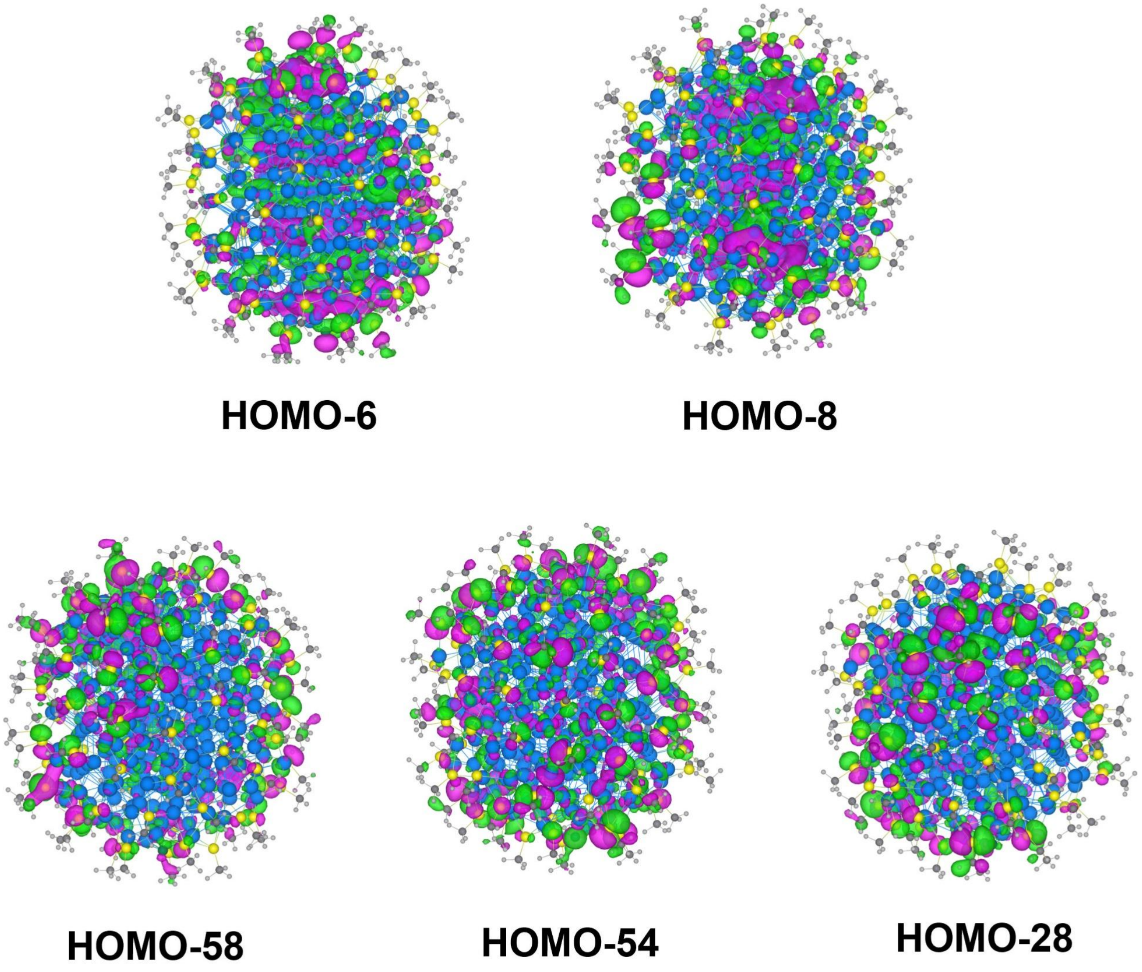

Figure S52. HOMO distributions of Ag_{252}.

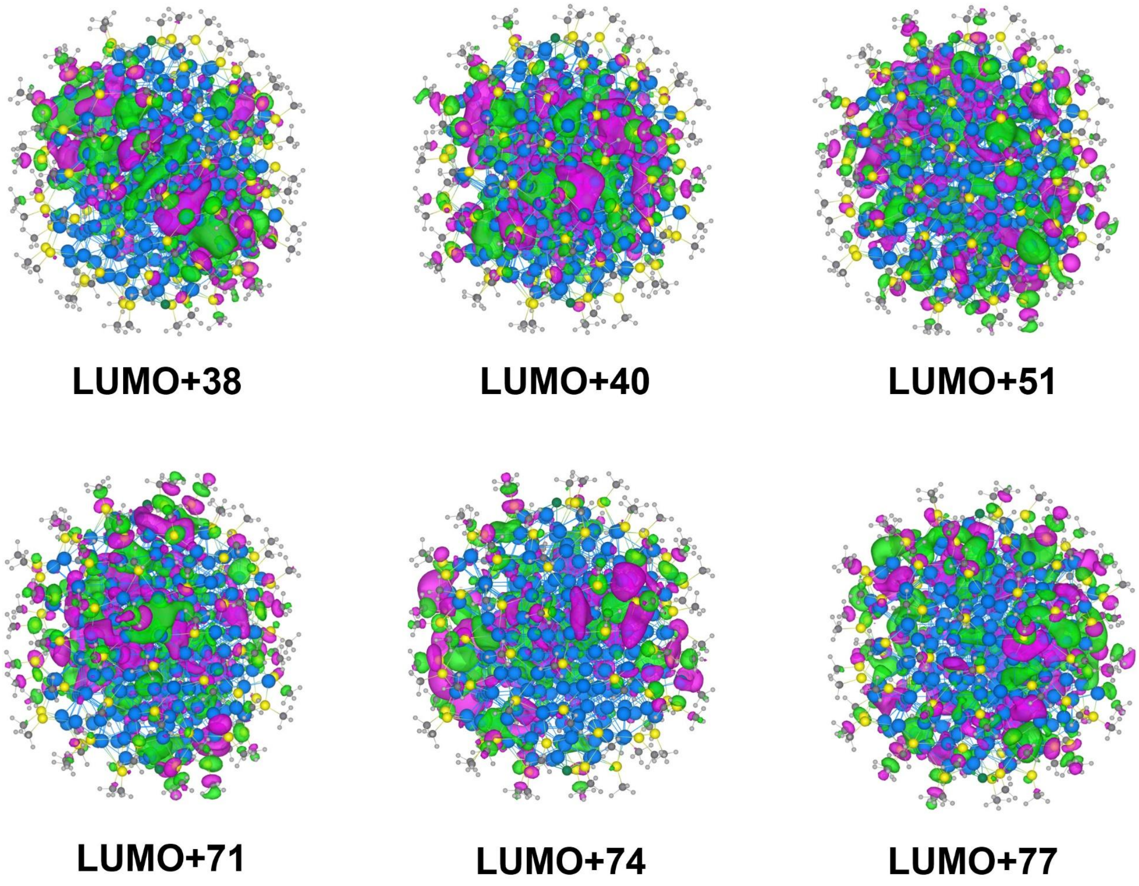

Figure S53. LUMO distributions of Ag_{252}.

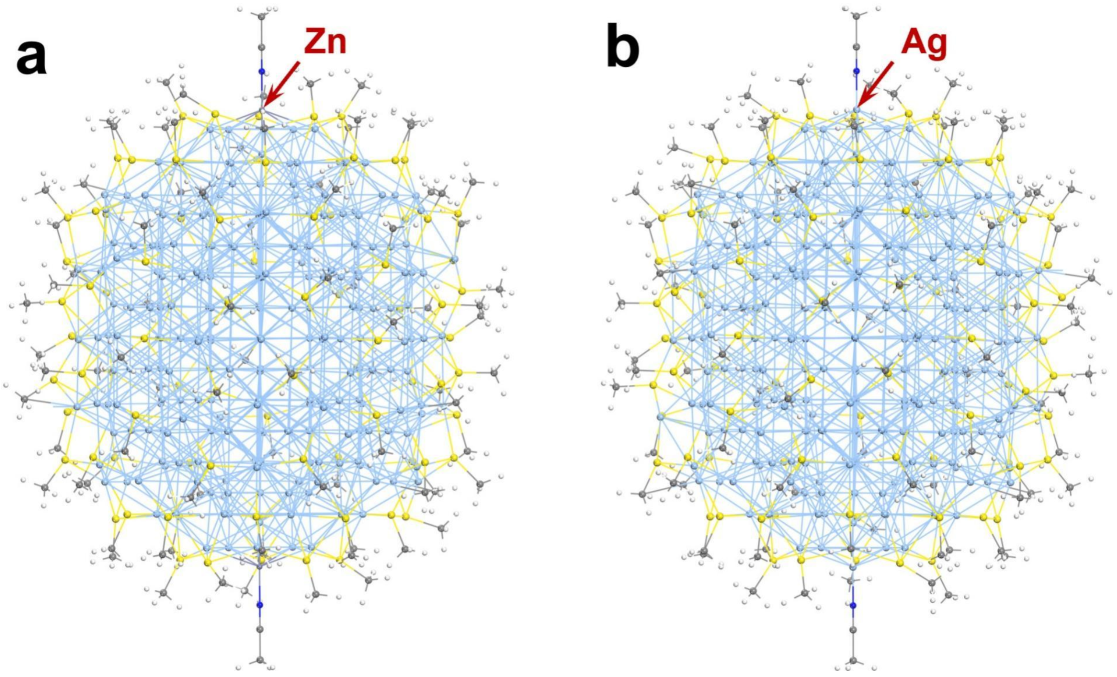

Figure S54. Computational models. The top views of (A) $Ag_{252}Zn_2$ and (B) Ag_{254} computational models after replacing all alkyl groups with R = CH_3.

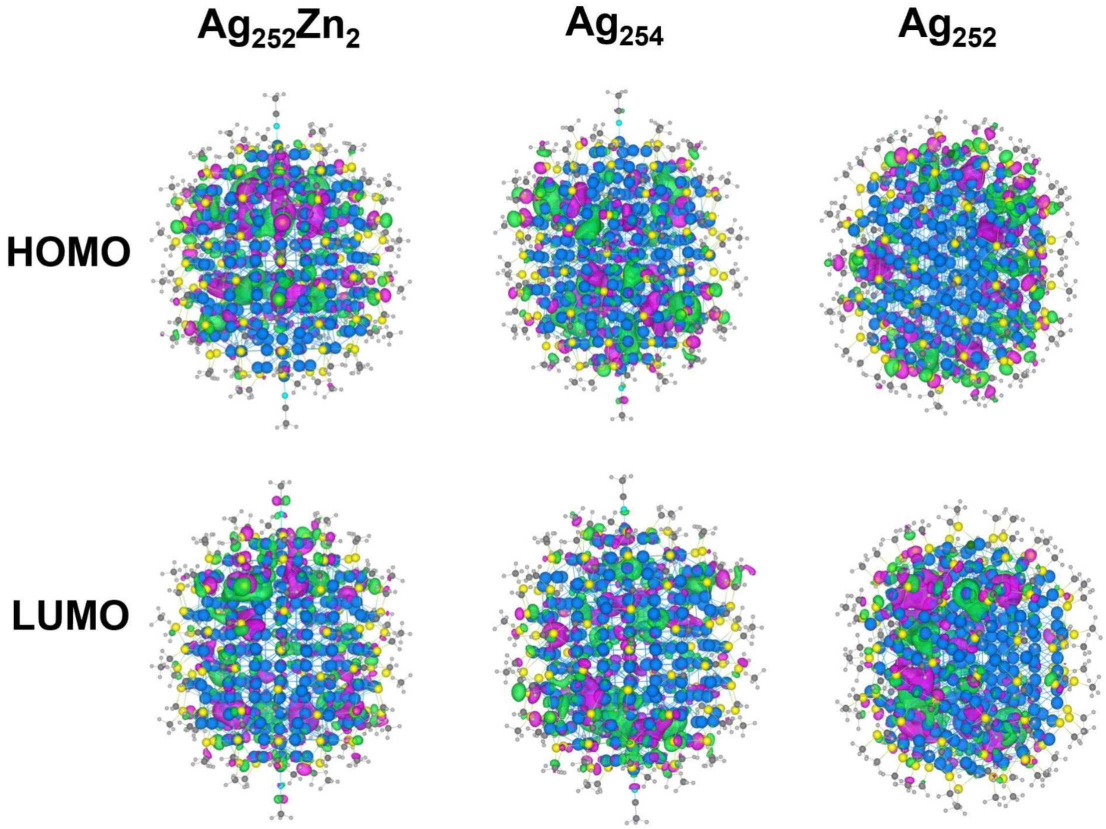

Figure S55. HOMO and LUMO distributions of $Ag_{252}Zn_2$, Ag_{254}, and Ag_{252}.

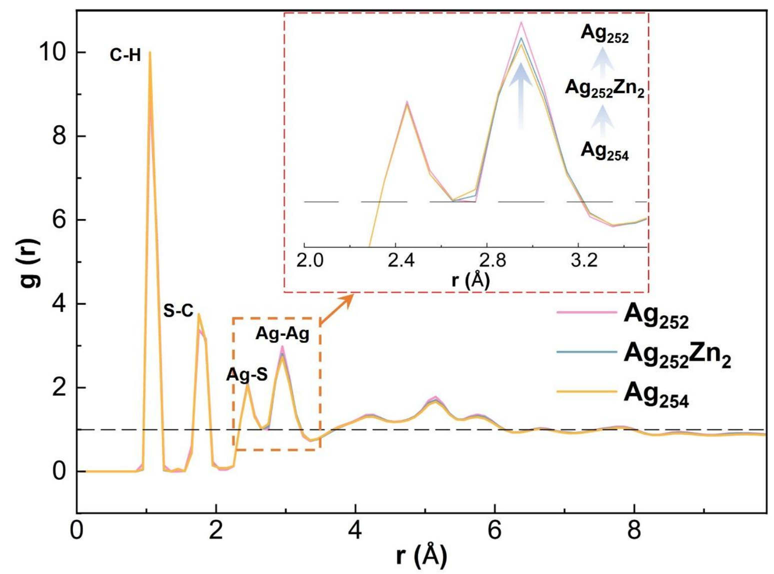

Figure S56. Radial distribution function of Ag_{252}, $Ag_{252}Zn_2$ and Ag_{254}. The radial distribution function, g(r), represents the relative probability density of finding a atom at a distance. A sharper and more intense peak in g(r) indicates a more well-defined and stable bond.

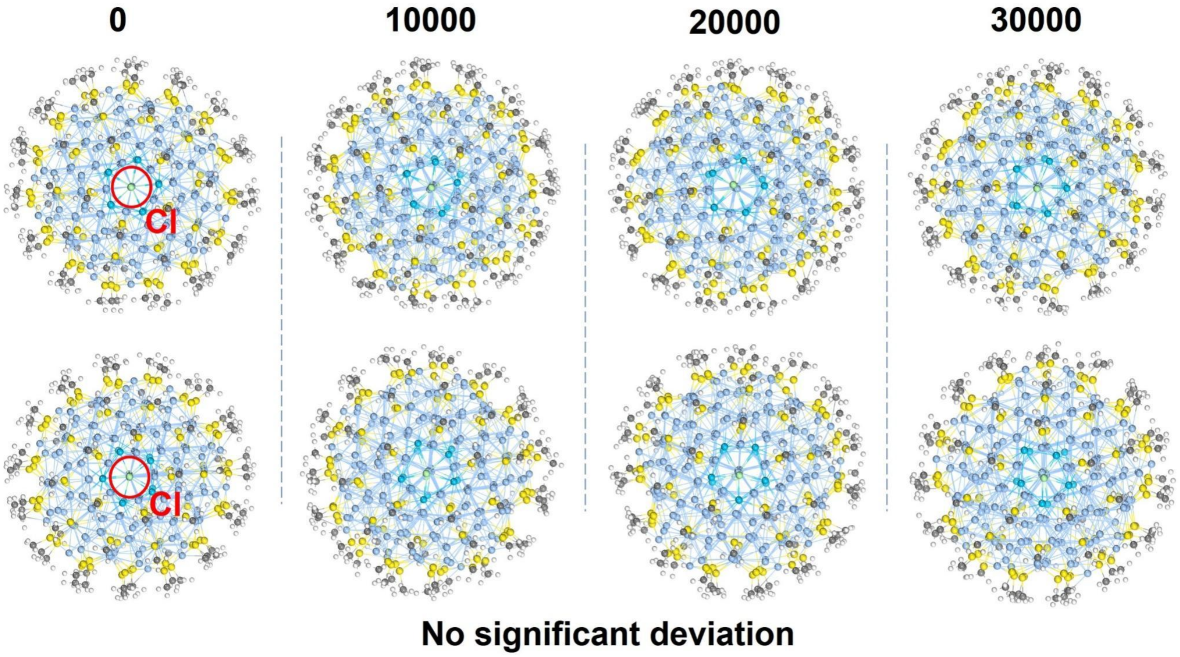

Figure S57. AIMD simulation was performed for the chlorine atoms at the two terminal positions of the Ag_{252} structure at 298.15 K, with a time step of 1 fs for a total of 30000 steps.

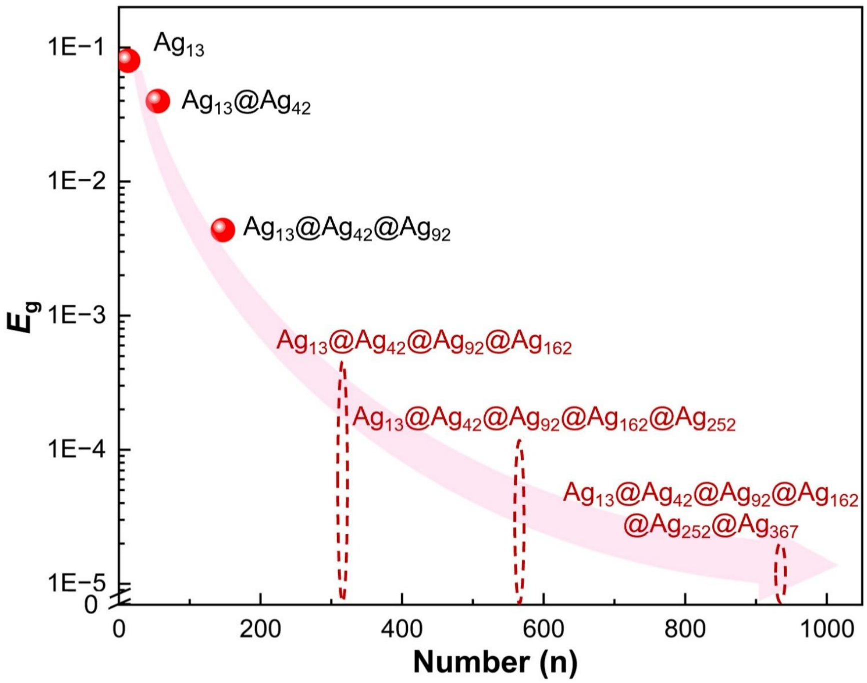

Figure S58. The progressive order gradient layers corresponds to the decreasing trend of E_g.

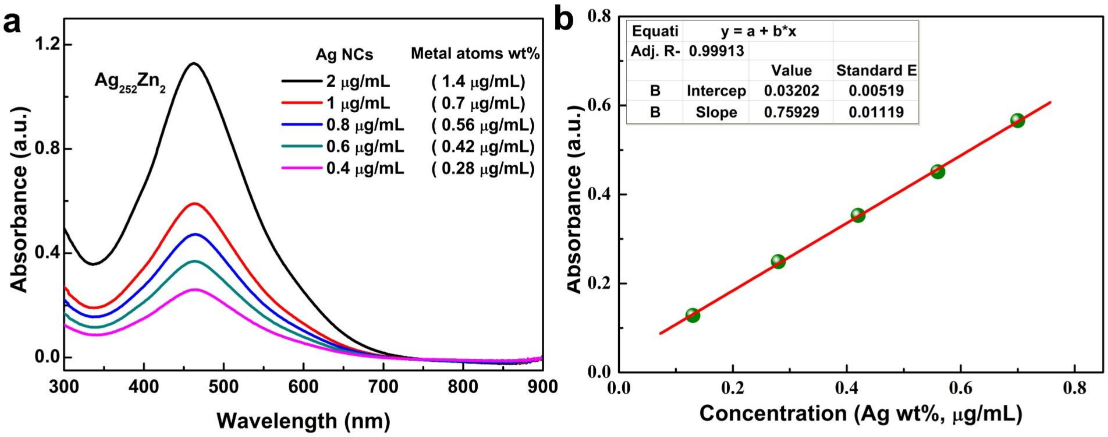

Figure S59. Optical properties of $Ag_{252}Zn_2$. (a) UV–vis–NIR absorption spectra of $Ag_{252}Zn_2$ with different concentrations; (b) the relationship between concentrations (on the basis of metal atoms) and absorbances of $Ag_{252}Zn_2$ monitored at 450 nm. Note that, the absorption coefficient is ε = 808.6 $L \cdot g^{-1} \cdot cm^{-1}$ at 450 nm, calculated according to Beer's law, $A = \varepsilon cl$, where $l = 1$ cm.

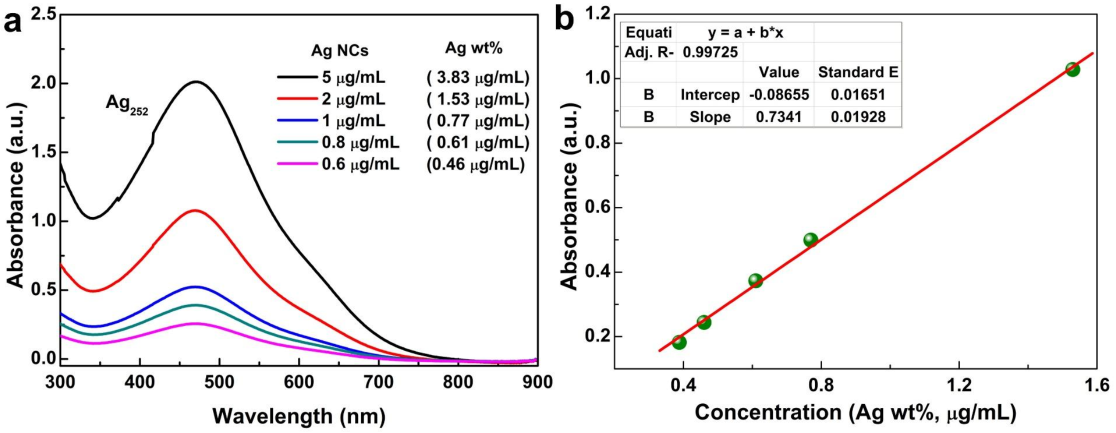

Figure S60. Optical properties of Ag_{252}. (a) UV–vis–NIR absorption spectra of Ag_{252} with different concentrations; (b) the relationship between concentrations (on the basis of metal atoms) and absorbances of Ag_{252} monitored at 450 nm. Note that, the absorption coefficient is ε = 648.1 $L \cdot g^{-1} \cdot cm^{-1}$ at 450 nm, calculated according to Beer's law, $A = \varepsilon c l$, where l = 1 cm.

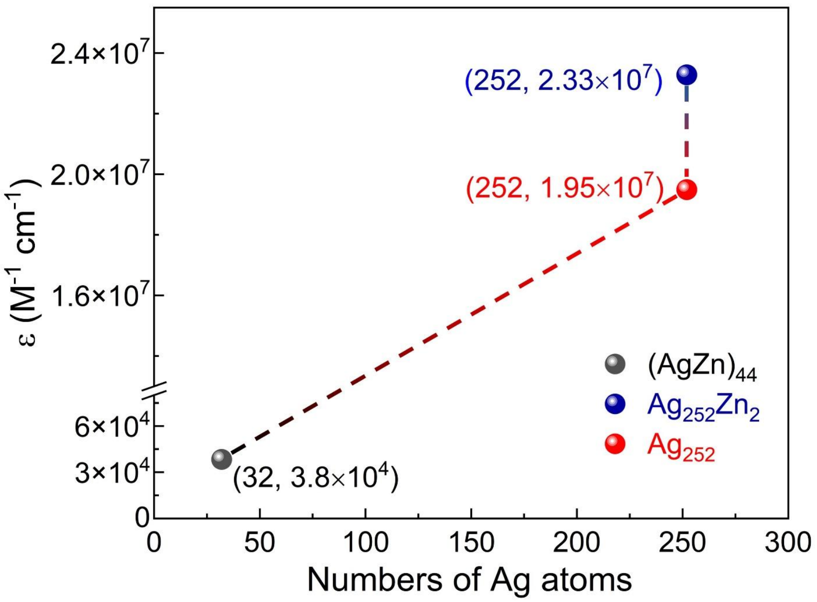

Figure S61. The relationship between molar extinction coefficients and numbers of Ag atoms.

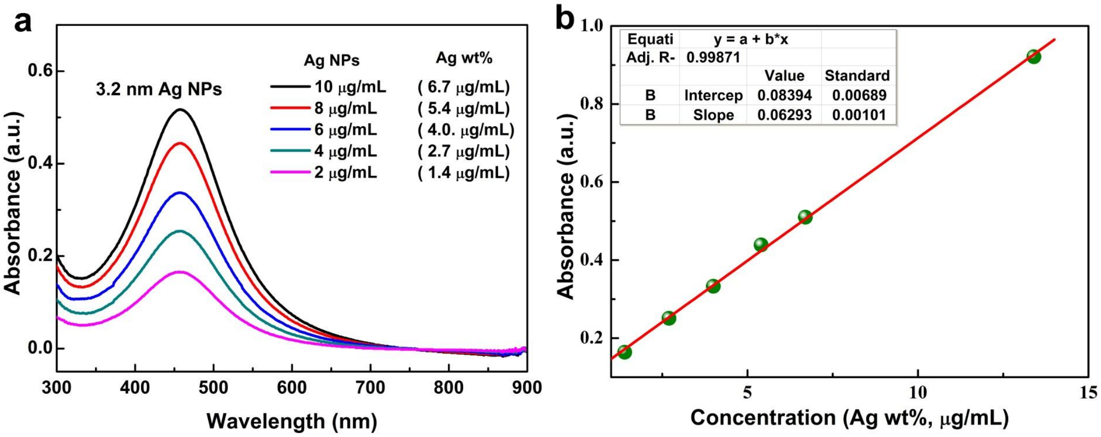

Figure S62. Optical properties of ~3.2 nm Ag nanocrystals. (a) UV–vis–NIR absorption spectra of ~3.2 nm Ag nanocrystals with different concentrations; (b) the relationship between concentrations (on the basis of metal atoms) and absorbances of Ag NPs monitored at 450 nm. Note that, the absorption coefficient is ε = 76.1 $L \cdot g^{-1} \cdot cm^{-1}$ at 450 nm, calculated according to Beer's law, $A = \varepsilon c l$, where l = 1 cm.

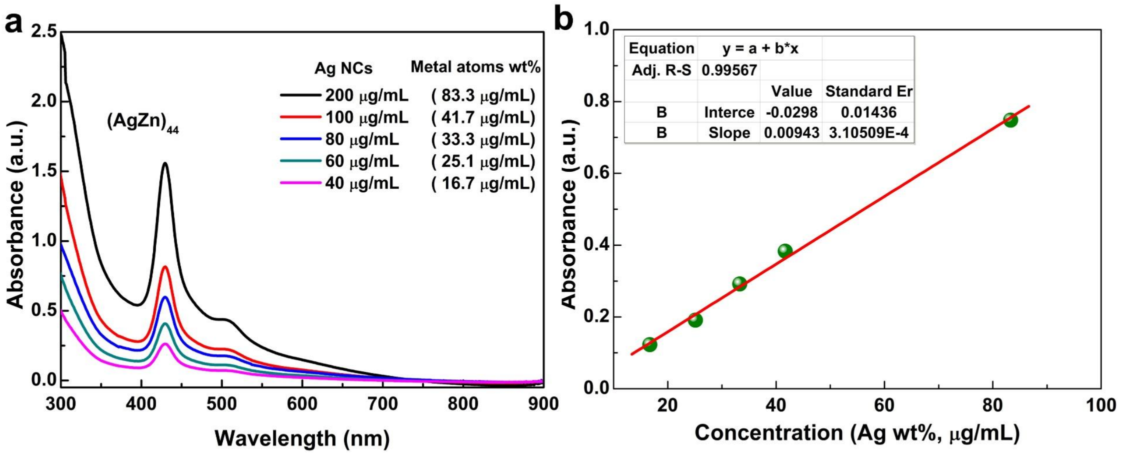

Figure S63. Optical properties of $(AgZn)_{44}$. (a) UV–vis–NIR absorption spectra of $(AgZn)_{44}$ with different concentrations; (b) the relationship between concentrations (on the basis of metal atoms) and absorbances of $(AgZn)_{44}$ monitored at 450 nm. Note that, the absorption coefficient is ε = 9.2 L·g^{-1}·cm^{-1} monitored at 450 nm, calculated according to Beer's law, $A = \varepsilon c l$, where l = 1 cm.

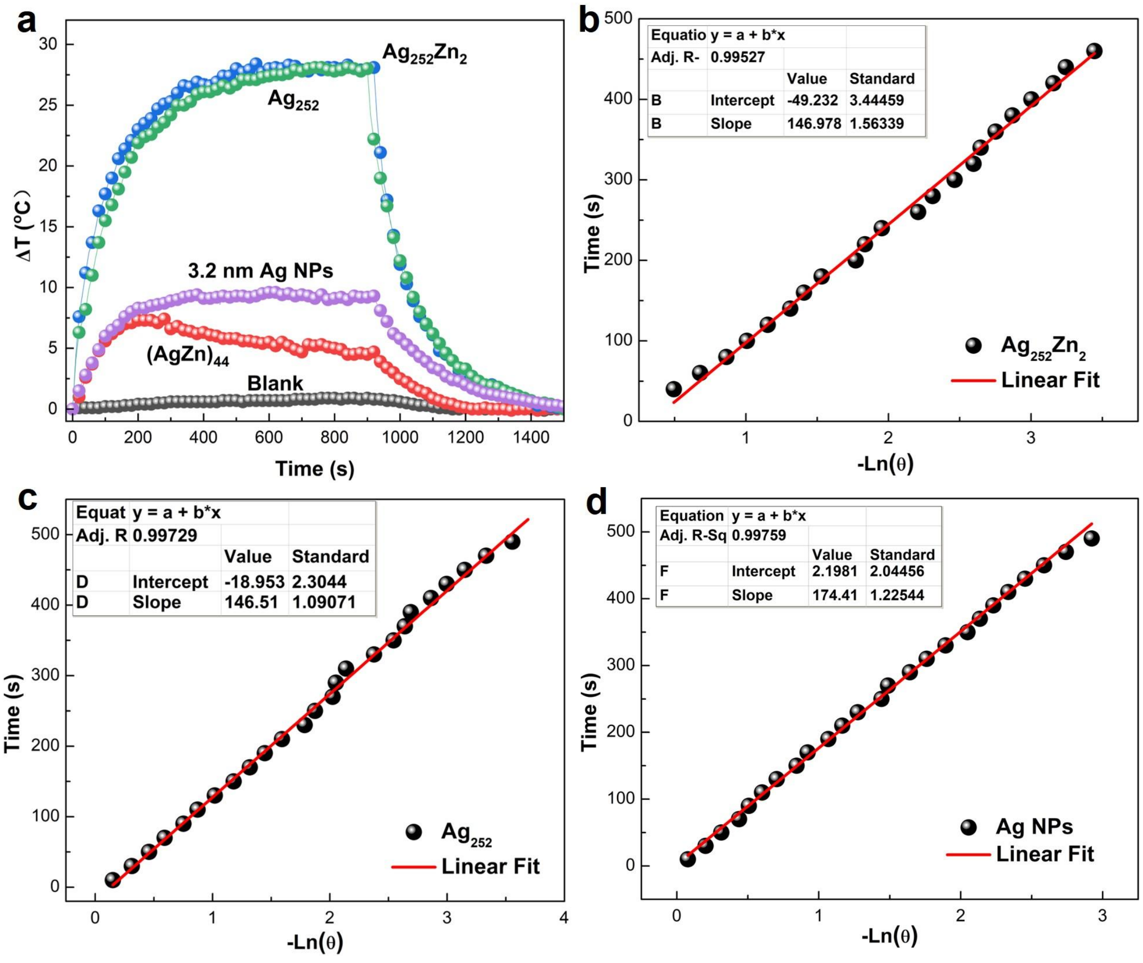

Figure S64. Photothermal properties. (a) Temperature change and (b) the time-lnθ linear curve of of $(AgZn)_{44}$, $Ag_{252}Zn_2$, Ag_{252} and ~3.2 nm Ag nanocrystals toluene solutions after irradiation. Concentration: 1.5 μg/mL (based on metal atoms); laser irradiation: 450 nm, 0.6 W cm^{-2}. Note: $(AgZn)_{44}$ is unstable at high temperatures and thus the time-lnθ curve could not be accurately plotted.

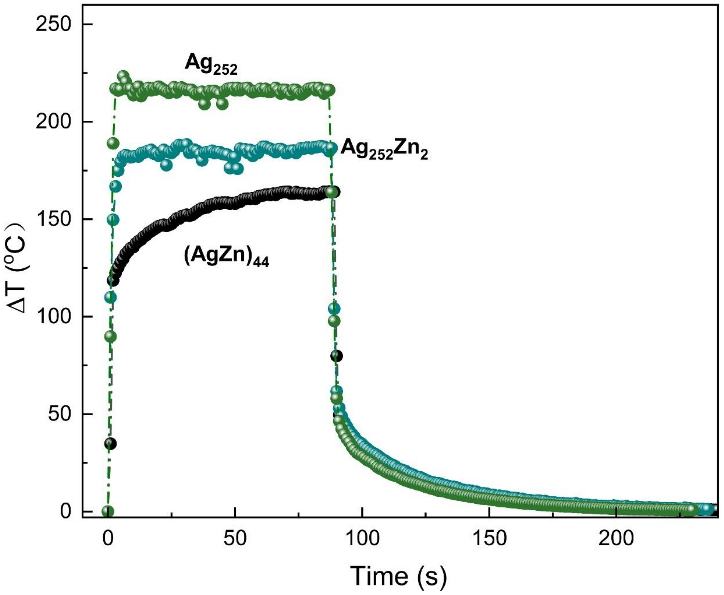

Figure S65. Temperature changes during irradiation treatment. Time-dependent temperature changes of $(AgZn)_{44}$, $Ag_{252}Zn_2$, and Ag_{252} crystals with the same metal atoms weight (1.5 mg). Laser irradiation: 450 nm, 0.6 W.

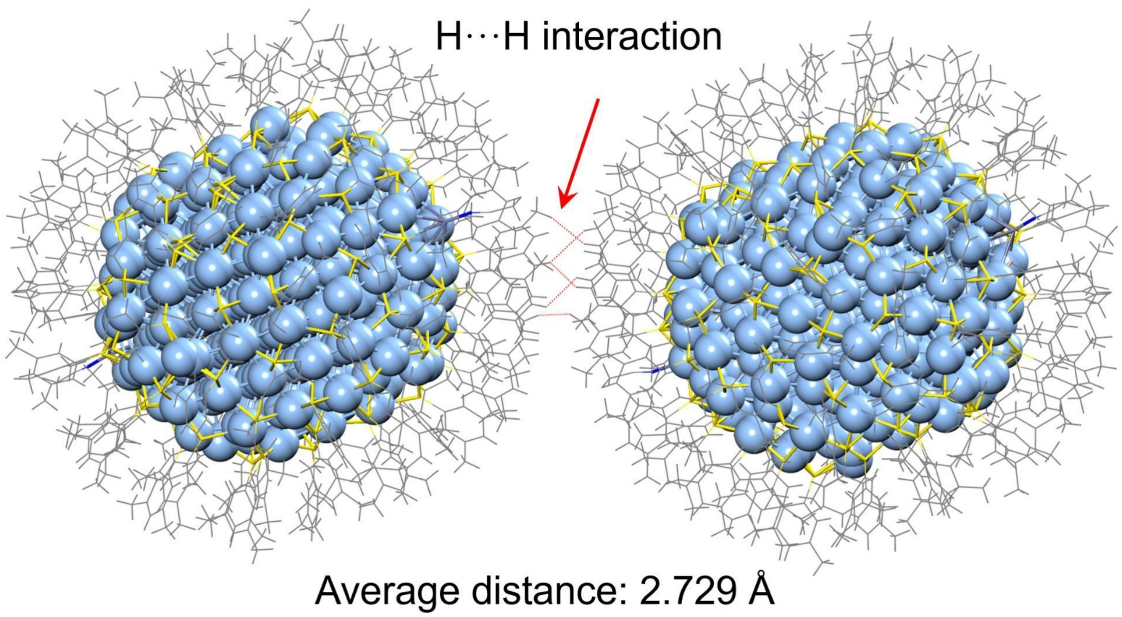

Figure S66. The average ligand H · · · H interaction distance between two adjacent $Ag_{252}Zn_2$ particles.

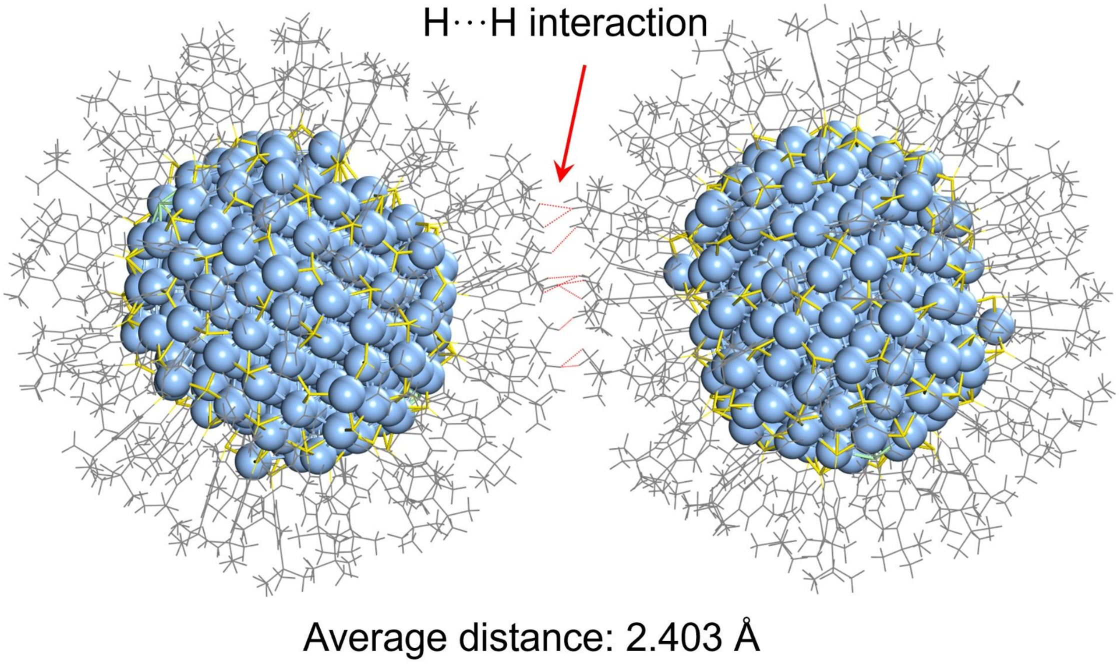

Figure S67. The average ligand H · · · H interaction distance between two adjacent Ag_{252} particles.

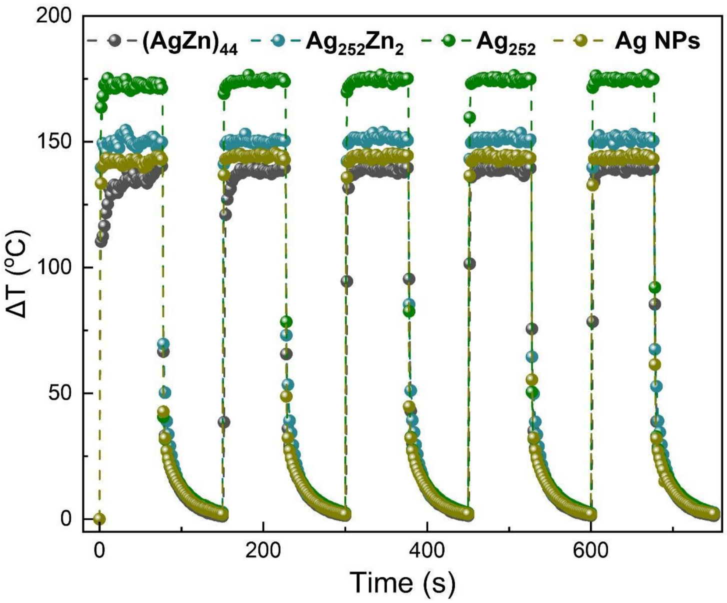

Figure S68. Temperature changes during irradiation treatment. Time-dependent temperature changes of amorphous $(AgZn)_{44}$, $Ag_{252}Zn_2$, Ag_{252}, and ~3.2 nm Ag nanocrystals with the same metal atoms weight (1.5 mg). Laser irradiation: 450 nm, 0.6 W.

Table S1. Partial characteristic information associated with the reported, structure-resolved nanoparticles with metal atom number larger than 100 per particle. Note, Mn represents the abbreviation of nanoparticle molecular formula, in which M denotes metal Au, Ag or Cu, and n denotes the number of metal atoms per particle.

Nanoparticles (M_n for short)	Crystal output (mg)	Yield (%)	Residual factor (R_1%)	Maximum absorption wavelength (nm)	Metallic or molecular (criterion)	Ref.
Ag_{102}	11.54	10	8.44	354, 405, 521	\	[10]
Ag_{110}	1.06	5	19.76	383,523	\	[11]
Ag_{112}	1.5	7	11.52	389, 424, 482, 580, 710	\	[12]
$Ag_{125}Cu_8$	\	\	12.88	388, 550. 800	\	[13]
Ag_{136}	\	\	8.36	450	Molecular (UV, DFT)	[14]
Ag_{141}	11.45	20	8.5	460	Molecular (UV, DFT)	[15]
Ag_{146}	\	\	10.35	446, 490, 580, 785	Molecular (UV, TA)	[16]
Ag_{152}	\	\	\	460	\	[17]
Ag_{155}	0.6	5	10.31	341, 460, 573	Molecular (UV, DFT)	[18]
Ag_{180}	4.48	15	9.15	346, 423	\	[19]
Ag_{192}	\	\	14.53	352	\	[20]
$Ag_{135}Cu_{60}$	3.73	10	7.05	407, 500, 643, 731	Molecular and metallic (UV, TA)	[21]
Ag_{206}	4.26	25	10.83	464	Metallic (UV, DPV, DFT)	[22]
$Ag_{210/211}$	0.833	5	11.96	460	Metallic (UV, DFT)	[23]
Ag_{213}	5.16	13.8	7.35	454	Metallic (UV)	[24]
Ag_{307}	3.08	10	8.51	473	Metallic (UV,	[25]

					DPV)	
Ag_{374}	\	\	9.51	465	Metallic (UV, DFT)	[14]
Au_{102}	\	\	\	\	Molecular (UV, DFT)	[26]
Au_{103}	\	\	9.37	434, 542, 634, 744	Molecular (UV, DPV, TA)	[27]
Au_{106}	10.4	12	8.72	470, 620	\	[28]
$Au_{41}Cu_{66}$	\	\	6.44	441, 495, 616	\	[29]
$Au_{57}Ag_{53}$	5.4	27	10.64	396, 462, 558	\	[30]
$Au_{80}Ag_{30}$	\	\	8.01	406, 460, 570	\	[31]
Au_{110}	3.6	13	8.36	480, 560	Molecular (UV, TA)	[32]
$Au_{52}Cu_{72}$	\	\	3.88	365, 396, 470, 578, 720	\	[33]
Au_{126}	\	\	9.61	375, 470, 565, 700	\	[34]
Au_{127}	\	\	11.75	395, 470, 700	\	[34]
$Au_{130\text{-}x}Ag_x$	\	\	13.77	523, 764	Molecular (UV, TA)	[35]
Au_{130}	\	\	\	360, 507, 708	\	[36]
Au_{133}	\	\	8.65	336, 421, 503, 712	Molecular (UV, TA)	[37]
Au_{133}	\	\	8.41	\	\	[38]
Au_{138}	\	\	5.67	367,465	Molecular and metallic (UV, TA)	[39]
$Au_{144\text{-}x}Ag_x$	\	\	\	310, 425, 560	\	[40]
Au_{144}	\	\	5.98	355, 475, 595, 715	\	[41]
Au_{144}	8.4	34	5.04	560, 620	\	[42]
Au_{146}	\	\	\	\	\	[43]

Au_{156}	\	\	11.89	483, 557, 668	Molecular and metallic (UV, DPV, TA)	[44]
Au_{187}	\	\	\	\	\	[45]
Au_{188}	\	\	\	407, 470	Molecular (TA, DFT, UV)	[46]
Au_{191}	\	\	5.73	450, 520	Molecular and metallic (UV, DFT, TA)	[47]
Au_{246}	\	\		460, 600	Molecular (UV, TA)	[48]
$(AuAg)_{267}$	2.27	15	7.3	460	Metallic (UV, DPV, DFT)	[49]
Au_{279}	\	\	13.72	510	Metallic (TA, UV)	[50]
Au_{329}	\	\	\	495	\	[51]
Au_{333}	\	\	\	\	\	[52]
$Au_{\sim500}$	\	\	\	500	\	[53]
$Au_{940\pm20}$	\	\	\	500	\	[54]
**$Ag_{252}Zn_2$**	**2401.1**	**52.8**	**5.65**	**460**	**Molecular (UV, TA, DFT)**	**This work**
**Ag_{252}**	**1030.3**	**21.3**	**3.61**	**465**	**Molecular (UV, TA, DFT)**	**This work**

“\” : not provided.

Table S2. Bond length analyses of the metal kernel of the $Ag_{252}Zn_2$ nanoparticle.

Position	N	Average (Å)	SD	Range (Å)
Ag_{13}	35	2.884	0.031	2.842-2.944
Ag_{13}-Ag_{42}	92	2.883	0.033	2.837-2.972
Ag_{42}	100	2.895	0.039	2.807-3.003
Ag_{42}-Ag_{92}	232	2.895	0.052	2.749-3.003
Ag_{92}	222	2.886	0.054	2.752-3.162

N is the number of bonds, and SD is the standard deviation of bond length.

Table S3. Bond length analyses of the metal kernel of the Ag_{252} nanoparticle.

Position	N	Average (Å)	SD	Range (Å)
**Ag_{13}**	35	2.879	0.021	2.838-2.923
**Ag_{13}-Ag_{42}**	92	2.877	0.029	2.831-2.972
**Ag_{42}**	100	2.885	0.032	2.814-3.003
**Ag_{42}-Ag_{92}**	232	2.888	0.057	2.776-3.003
**Ag_{92}**	222	2.876	0.047	2.744-3.171

N is the number of bonds, and SD is the standard deviation of bond length.

Table S4. Ag–Ag/Ag–S bond length analyses of the staples in the $Ag_{252}Zn_2$ nanoparticle.

Position	Bond	N	Average (Å)	SD	Range (Å)
**Top staples**	Ag-Ag	110	3.102	0.141	2.897-3.593
	Ag-S	155	2.588	0.125	2.449-2.864
**Side staples**	Ag-Ag	45	3.132	0.082	3.001-3.264
	Ag-S	85	2.546	0.187	2.314-2.802

N is the number of bonds, and SD is the standard deviation of bond length.

Table S5. Ag–Ag/Ag–S bond length analyses of the staples in the Ag_{252} nanoparticle.

Position	Bond	N	Average (Å)	SD	Range (Å)
**Top staples**	Ag-Ag	110	3.116	0.23	2.836-3.614
	Ag-S	155	2.539	0.154	2.233-2.834
**Side staples**	Ag-Ag	45	3.11	0.196	2.967-3.675
	Ag-S	85	2.598	0.116	2.389-2.776

N is the number of bonds, and SD is the standard deviation of bond length.

Table S6. Kinetic fitting results. The time constants extracted from kinetic fitting results for different samples with different excitation power (“*” means null).

Sample	Power(nJ/pulse)	τ_1 (ps)	τ_2 (ps)	τ_3 (ps)
$(AgZn)_{44}$	50	0.34 ± 0.05	305 ± 110	> 7500
	83	0.26 ± 0.03	792 ± 185	> 7500
	130	0.27 ± 0.03	332 ± 94	> 7500
	191	0.25 ± 0.02	476 ± 89	> 7500
	445	0.24 ± 0.01	603 ± 52	> 7500
	600	0.26 ± 0.01	478 ± 27	> 7500
$Ag_{252}Zn_2$	50	1.40 ± 0.16	10.4 ± 1.7	608 ± 31
	83	1.31 ± 0.15	6.3 ± 0.8	557 ± 19
	130	1.32 ± 0.12	6.4 ± 0.7	509 ± 15
	191	1.42 ± 0.05	17.7 ± 1.5	531 ± 17
	445	1.41 ± 0.02	11.8 ± 0.5	560 ± 15
	600	1.42 ± 0.03	9.4 ± 0.6	652 ± 22
Ag_{252}	50	0.86 ± 0.67	5.2 ± 1.9	650 ± 113
	83	1.32 ± 0.32	8.1 ± 2.5	554 ± 62
	130	1.06 ± 0.34	4.9 ± 1.0	568 ± 46
	191	1.26 ± 0.13	6.9 ± 1.0	577 ± 29
	445	1.39 ± 0.10	7.0 ± 0.7	535 ± 25
	600	1.30 ± 0.06	6.3 ± 0.5	495 ± 17
3.2 nm Ag NPs	50	1.10 ± 0.04	70 ± 19	*
	83	1.19 ± 0.04	164 ± 28	*
	130	1.28 ± 0.03	231 ± 30	*
	191	1.30 ± 0.02	169 ± 11	*
	445	1.34 ± 0.02	127 ± 10	*
	600	1.40 ± 0.02	245 ± 21	*

Table S7. Kinetic fitting results of $Ag_{252}Zn_2$. The time constants extracted from kinetic fitting results of $Ag_{252}Zn_2$ excited with different wavelengths.

Excitation	Power(nJ/pulse)	τ_1 (ps)	τ_2 (ps)	τ_3 (ps)
**500 nm**	50	1.33 ± 0.14	8.0 ± 1.4	503 ± 19
	83	1.25 ± 0.09	6.7 ± 0.6	536 ± 12
	130	1.32 ± 0.06	7.3 ± 0.5	521 ± 11
	191	1.25 ± 0.04	8.1 ± 0.4	521 ± 9
	445	1.31 ± 0.02	9.1 ± 0.4	497 ± 11
	600	1.32 ± 0.03	10.5 ± 0.5	468 ± 12
**600 nm**	50	1.14 ± 0.43	8.2 ± 3.7	564 ± 46
	83	1.59 ± 0.30	13.3 ± 5.1	601 ± 54
	130	1.31 ± 0.12	7.4 ± 0.9	537 ± 20
	191	1.44 ± 0.05	10.8 ± 0.8	545 ± 14
	445	1.34 ± 0.02	8.4 ± 0.4	540 ± 9
	600	1.29 ± 0.02	9.1 ± 0.4	533 ± 14

Table S8. Kinetic fitting results of $Ag_{252}Zn_2$. The time constants extracted from kinetic fitting results of $Ag_{252}Zn_2$ probed with different wavelengths under 400 nm excitation ("/" means invalid).

Power(nJ/pulse)	Probe wavelength(nm)	τ_1 (ps)	τ_2 (ps)	τ_3 (ps)
	440	1.33 ± 0.12	11.7 ± 2.8	635 ± 83
	460	1.59 ± 0.23	10.3 ± 2.6	599 ± 41
**50**	480	1.15 ± 0.25	7.0 ± 1.1	530 ± 34
	620	1.39 ± 0.59	5.3 ± 2.4	606 ± 169
	660	1.31 ± 0.42	/	/
	440	1.16 ± 0.18	7.8 ± 1.3	515 ± 40
	460	1.39 ± 0.22	7.4 ± 1.2	587 ± 32
**83**	480	1.12 ± 0.25	6.5 ± 1.0	507 ± 22
	620	1.32 ± 2.77	5.2 ± 1.7	523 ± 131
	660	1.00 ± 0.37	/	/
	440	1.23 ± 0.16	6.7 ± 1.5	492 ± 35
	460	1.42 ± 0.20	7.0 ± 1.3	499 ± 22
**130**	480	1.47 ± 0.18	11.9 ± 3.6	559 ± 25
	620	1.08 ± 0.75	/	/
	660	0.90 ± 0.32	/	/
	440	1.19 ± 0.08	8.5 ± 1.0	555 ± 30
	460	1.43 ± 0.04	28.3 ± 2.8	499 ± 21
**191**	480	1.35 ± 0.07	25.3 ± 3.8	542 ± 23
	620	1.23 ± 0.37	6.4 ± 1.8	703 ± 114
	660	1.04 ± 0.20	7.0 ± 2.6	407 ± 74
	440	1.28 ± 0.04	11.5 ± 1.0	437 ± 27
	460	1.45 ± 0.03	12.3 ± 1.0	454 ± 19
**445**	480	1.34 ± 0.04	13.4 ± 1.1	465 ± 17
	620	1.35 ± 0.36	4.9 ± 0.7	817 ± 91
	660	1.51 ± 0.14	11.8 ± 4.0	657 ± 128

	440	1.30 ± 0.04	11.5 ± 1.0	438 ± 28
	460	1.44 ± 0.03	11.6 ± 0.9	457 ± 18
**600**	480	1.35 ± 0.04	14.4 ± 1.2	467 ± 18
	620	1.04 ± 0.28	5.0 ± 0.5	786 ± 92
	660	1.43 ± 0.15	9.5 ± 2.8	682 ± 123

Table S9. The orbital distribution of $Ag_{252}Zn_2$ within the range from HOMO-22 to LUMO+40.

Orbital energy (eV)	Compositions				Orbital number
	Ag	S	Zn	other	
-1.1168	8.40917	10.77237	0	0	H-22
-1.0874	5.82285	12.6297	0	0	H-21
-1.0487	12.19095	16.53614	0	0	H-20
-0.9929	6.53366	9.03629	0.65434	0	H-19
-0.9805	6.76617	11.77214	0	0	H-18
-0.9501	7.92535	9.81156	0	0	H-17
-0.8757	10.4891	19.25457	0	0	H-16
-0.8516	13.64529	4.2202	0	0	H-15
-0.8208	8.95189	20.39132	0	0	H-14
-0.8116	9.01349	3.28608	0	0	H-13
-0.8095	8.96825	7.5877	0	0	H-12
-0.7727	11.90545	4.70205	0	0.52962	H-11
-0.7686	7.48101	2.03128	0	0	H-10
-0.755	7.37172	2.17869	0	0	H-9
-0.7339	6.59001	4.29041	0	0	H-8
-0.7149	7.00129	5.58793	0	0	H-7
-0.6926	7.72098	12.70636	0	0	H-6
-0.6781	6.87912	3.2791	0	0	H-5
-0.6283	21.06038	1.20727	3.3673	0	H-4
-0.6149	5.77452	4.5795	0	0	H-3
-0.5805	14.43036	3.61253	2.0958	1.23648	H-2
-0.5459	13.34117	3.30845	0.71969	0	H-1
-0.5365	10.55139	3.81745	0	0	H
-0.5345	13.19435	2.82411	0	0	L
-0.4963	13.73862	2.5417	0	0	L+1
-0.4789	13.60863	2.44884	0	0	L+2
-0.4584	9.30398	5.21652	0.50471	0	L+3
-0.4549	9.58956	2.34631	0	0	L+4
-0.432	17.48395	2.43682	0	0	L+5
-0.3822	13.93484	4.31343	4.48054	0	L+6

-0.3554	13.2242	1.26699	0	0	L+7
-0.3361	14.04505	1.65518	0	0	L+8
-0.293	3.29509	2.28799	2.1364	2.0457	L+9
-0.2423	5.07108	2.39393	1.15422	1.1555	L+10
-0.169	6.67105	0.63799	2.50637	4.19353	L+11
-0.1334	10.02115	0	1.71093	2.81267	L+12
-0.1023	18.52578	6.08504	0	0	L+13
-0.0506	19.01074	5.98061	0	0	L+14
-0.0374	3.35883	1.14545	2.39021	5.85765	L+15
-0.0132	5.45663	3.40974	1.75176	4.38024	L+16
0.0301	8.74407	1.61358	0	0	L+17
0.0579	7.68872	0.58228	0	0	L+18
0.0938	9.04849	0	0	0	L+19
0.1116	5.44162	0	2.56043	11.05214	L+20
0.1324	6.25724	0.54112	2.00837	3.68685	L+21
0.1398	12.71479	2.76437	3.67345	17.58733	L+22
0.1947	7.84604	0	0.81131	6.24295	L+23
0.2145	7.24931	0.62788	0.65081	3.95308	L+24
0.2655	9.89481	0	0	0.56306	L+25
0.2735	12.00697	0	0	0.84947	L+26
0.2812	7.45186	0.50073	0	2.89056	L+27
0.294	2.99371	0	1.76812	16.20607	L+28
0.3069	2.94776	0	2.56733	24.13525	L+29
0.3129	0.51211	0	3.50474	32.4509	L+30
0.3156	1.12304	0	2.07597	24.69971	L+31
0.3678	6.68426	0.56181	0.89451	15.47981	L+32
0.3949	14.35449	0	0	0	L+33
0.3993	6.56315	0	0	0	L+34
0.4154	6.7083	0	0	0	L+35
0.4284	9.83858	0	0	1.47028	L+36
0.4555	7.49369	0	0	0	L+37
0.4754	9.90836	0	0	0	L+38
0.4798	4.64394	0	0	0	L+39
0.5258	8.78942	1.52345	0	1.36731	L+40

Table S10. Calculation of photothermal conversion efficiency (*η*).

Sample	ΔT_{max} (°C)	A_{450} (a.u)	$τ_s$	*hS* (J)	*η* (%)
**$Ag_{252}Zn_2$**	28.3	1.18	146.98	0.010537488	53.21
**Ag_{252}**	28	1.02	146.51	0.010571929	54.54
**3.2 nm Ag NPs**	9.2	0.17	174.41	0.008880225	41.83

The PCE was determined according to the previous method [55]. Details are shown as follows:

$$\sum_i m^i C_{p,i} \frac{dT}{dt} = Q_s - Q_{loss}$$

where m_i (0.88 g) and $C_{p,i}$ [1.76 J (g °C)$^{-1}$] are the mass and heat capacity of system components, respectively. Q_s is the photothermal energy input into the samples using an NIR laser, and Q_{loss} is the thermal energy lost to the surroundings. When the temperature is at its maximum, the system is in balance.

$$Q_s = Q_{loss} = hS\Delta T_{max}$$

where *h* is the heat transfer coefficient, *S* is the surface area of the container, and ΔT_{max} is the maximum temperature change. The PCE (*η*) is calculated using the following equation:

$$\eta = \frac{hS\Delta T_{max}}{I(1 - 10^{-450})}$$

where *I* is the laser power (0.6 W cm^{-2}) and is the absorbance of the samples at the investigated wavelength.

To obtain the value of *hS*, a dimensionless driving force temperature *θ* is introduced as follows:

$$\theta = \frac{T - T_{surr}}{T_{max} - T_{surr}}$$

where *T* is the temperature of the samples, T_{max} is the maximum system temperature, and T_{surr} is the initial temperature of the surrounding.

If the sample system time constant $τ_s$ is

$$τ_s = \frac{\sum m_i C_{p,i}}{hS}$$

$$\frac{d\theta}{dt} = \frac{1}{τ_s} \frac{Q_s}{hS\Delta T_{max}} - \frac{\theta}{τ_s}$$

When the laser is off, $Q_s = 0$; therefore,

$$\frac{d\theta}{dt} = -\frac{\theta}{τ_s}$$

$$t = -τ_s \ln \theta$$

The value of *hS* can be obtained from the slope of the cooling time *vs* ln *θ* plot.

Table S11. The ligand H··· H interaction distances between two adjacent particles.

	$Ag_{252}Zn_2$	Ag_{252}	
**Inter H··· H distance (Å)**	2.533	2.518	1.945
	2.810	2.121	2.443
	2.857	2.836	2.674
	2.538	2.575	2.327
	2.907	2.433	2.161
**Average (Å)**	2.729	2.403	

Table S12. Crystal data and structure refinement for $(AgZn)_{44}$.

Identification code	$Ag_{32-x}Zn_{12+x}(TPP)_{4-y}(3,5\text{-DMBT})_{36}$
Empirical formula	$C_{342}H_{368}Ag_{32}P_3S_{36}Zn_{12}$
Formula weight	9961.69
Temperature	193 K
Wavelength	1.34139 Å
Crystal system	Triclinic
Space group	P-1
Unit cell dimensions	a = 23.6365(17) Å
	b = 23.9277(16) Å
	c = 39.800(3) Å
Volume	20199(3) Å^3
Z	2
Density (calculated)	1.638 Mg/m^3
Absorption coefficient	9.996 mm^{-1}
F(000)	9810
Theta range for data collection	1.776 to 54.262°.
Index ranges	-28<=h<=28, -28<=k<=28, -46<=l<=48
Reflections collected	218616
Independent reflections	74352 [R(int) = 0.1062]
Completeness to theta = 53.594°	99.9 %
Absorption correction	Semi-empirical from equivalents
Max. and min. transmission	0.7506 and 0.366
Data / restraints / parameters	74352/ 4843 / 3453
Goodness-of-fit on F^2	1.061
Final R indices [I>2sigma(I)]	R1 = 0.0934, wR2 = 0.2438
R indices (all data)	R1 = 0.1641, wR2 = 0.2857
Extinction coefficient	0.000104(7)
Largest diff. peak and hole	1.46 and -1.73 e.Å^{-3}

Table S13. Crystal data and structure refinement for $Ag_{252}Zn_2$.

Identification code	$Ag_{252}Zn_2$
Empirical formula	$C_{684}H_{771}Ag_{252}N_2S_{85}Zn_2$
Formula weight	39059.06
Temperature	193 K
Wavelength	1.34139 Å
Crystal system	Monoclinic
Space group	P 1 2/n 1
Unit cell dimensions	a = 35.5355(13) Å
	b = 31.3194(12) Å
	c = 46.0835(16) Å
Volume	50931(3) Å^3
Z	2
Density (calculated)	2.547 Mg/m^3
Absorption coefficient	27.107 mm^{-1}
F(000)	36306
Theta range for data collection	1.227 to 63.018°.
Index ranges	-40<=h<=45, -41<=k<=38, -54<=l<=59
Reflections collected	470686
Independent reflections	110699 [R(int) = 0.0963]
Completeness to theta = 53.594°	99.8 %
Absorption correction	Semi-empirical from equivalents
Max. and min. transmission	0.3012 and 0.1565
Data / restraints / parameters	110699 / 0 / 4696
Goodness-of-fit on F^2	1.025
Final R indices [I>2sigma(I)]	R1 = 0.0565, wR2 = 0.1363
R indices (all data)	R1 = 0.1009, wR2 = 0.1607
Extinction coefficient	n/a
Largest diff. peak and hole	1.029 and -1.092 e.Å^{-3}

Table S14. Crystal data and structure refinement for Ag_{252}.

Identification code	Ag_{252}
Empirical formula	$C_{838}H_{1081}Ag_{252}Cl_2S_{85}$
Formula weight	41133.20
Temperature	153 K
Wavelength	1.34139 Å
Crystal system	Orthorhombic
Space group	$P2_12_12$
Unit cell dimensions	a = 38.789(4) Å
	b = 47.323(5) Å
	c = 34.661(3) Å
Volume	63625(11) $Å^3$
Z	2
Density (calculated)	2.147 Mg/m^3
Absorption coefficient	21.726 mm^{-1}
F(000)	38694
Theta range for data collection	1.487 to 63.652°.
Index ranges	-43<=h<=49, -58<=k<=61, -39<=l<=45
Reflections collected	781034
Independent reflections	146332 [R(int) = 0.1218]
Completeness to theta = 53.594°	99.9 %
Absorption correction	Semi-empirical from equivalents
Max. and min. transmission	0.11 and 0.09
Data / restraints / parameters	146332 / 0 / 5438
Goodness-of-fit on F^2	1.004
Final R indices [I>2sigma(I)]	R1 = 0.0361, wR2 = 0.0527
R indices (all data)	R1 = 0.0668, wR2 = 0.0671
Extinction coefficient	n/a
Largest diff. peak and hole	1.02 and -0.58 e.$Å^{-3}$

References

1. Grimme S. A simplified Tamm-Dancoff density functional approach for the electronic excitation spectra of very large molecules. *J Chem Phys* 2013; **138**: 244104.
2. Neese F. Software update: The ORCA program system—Version 5.0. *WIREs Comput Mol Sci* 2022; **12**: e1606.
3. Lippert BG, Parrinello JH, Michele P. A hybrid Gaussian and plane wave density functional scheme. *Molec Phys* 2010; **92**: 477–88.
4. Lu T. A comprehensive electron wavefunction analysis toolbox for chemists, Multiwfn. *J Chem Phys* 2024; **161**: 082503.
5. Lu T, Chen F, Multiwfn: a multifunctional wavefunction analyzer. *J Comput Chem* 2012; **33**: 580–92.
6. Frisch MJ, Trucks GW, Schlegel HB *et al.* Gaussian 16, Revision B.01, Gaussian, Inc., Wallingford CT, 2016.
7. Perdew JP, Burke K, Ernzerhof M. Generalized gradient approximation made simple. *Phys Rev Lett* 1996; **77**: 3865–8.
8. Grimme S, Ehrlich S, Goerigk L. Effect of the damping function in dispersion corrected density functional theory. *J Comput Chem* 2011; **32**: 1456–65.
9. Weigend F, Ahlrichs R. Balanced basis sets of split valence, triple zeta valence and quadruple zeta valence quality for H to Rn: Design and assessment of accuracy. *Phys Chem Chem Phys* 2005; **7**: 3297–305.
10. Wang Z, Wang Y, Zhang C *et al.* Silvery fullerene in Ag_{102} nanosaucer. *Natl Sci Rev* 2024; **11**: nwae192.
11. Qu M, Zhang FQ, Zhang GL *et al.* Cocrystallization-driven formation of fcc-based Ag_{110} with Chinese triple Luban Lock shape. *Angew Chem Int Ed* 2024; **63**: e202318390.
12. Hu F, Li J, Guan Z, Yuan S, Wang Q-M. Formation of an alkynyl-protected Ag_{112} silver nanocluster as promoted by chloride released in situ from CH_2Cl_2. *Angew Chem Int Ed* 2020; **59**: 5312–5.
13. Ma X, Zhang Q, Li J *et al.* Bimetallic $Ag_{125}Cu_8$ nanocluster, structure determination, and nonlinear optical properties. *Inorg Chem* 2024; **63**: 8775–81.
14. Yang H, Wang Y, Chen X *et al.* Plasmonic twinned silver nanoparticles with molecular precision. *Nat Commun* 2016; **7**: 12809.

15. Ren L, Yuan P, Su H *et al.* Bulky surface ligands promote surface reactivities of $[Ag_{141}X_{12}(SAdm)_{40}]^{3+}$ (X = Cl, Br, I) nanoclusters: models for multiple-twinned nanoparticles. *J Am Chem Soc* 2017; **139**: 13288–91.
16. Song Y, Lambright K, Zhou M *et al.* Large-scale synthesis, crystal structure, and optical properties of the $Ag_{146}Br_2(SR)_{80}$ nanocluster. *ACS Nano* 2018; **12**: 9318–25.
17. Chakraborty I, Govindarajan A, Erusappan J *et al.* The superstable 25 kDa monolayer protected silver nanoparticle: measurements and interpretation as an icosahedral $Ag_{152}(SCH_2CH_2Ph)_{60}$ cluster. *Nano Lett* 2012; **12**: 5861–6.
18. Wang Z, Alkan F, Aikens C M *et al.* An ultrastable 155-nuclei silver nanocluster protected by thiacalix[4]arene and cyclohexanethiol for photothermal conversion. *Angew Chem Int Ed* 2022; **61**: e202206742.
19. Wang Z, Su H-F, Tan Y-Z *et al.*, Assembly of silver Trigons into a buckyball-like Ag_{180} nanocage. *Proc Natl Acad Sci USA* 2017; **114**: 12132–7.
20. Su Y-M, Wang Z, Tung C-H, Sun D, Schein S. Keplerate Ag_{192} cluster with 6 silver and 14 chalcogenide octahedral and tetrahedral shells. *J Am Chem Soc* 2021; **143**: 13235–44.
21. Wang S, Tang L, Dong W *et al.* Structure and optical properties of $Ag_{135}Cu_{60}$ nanocluster incorporating an Ag_{135} fullerene wrapped by copper complexes. *Nat Synthes* 2024; **4**: 506–13.
22. Yan J, Zhang J, Chen X *et al.* Thiol-stabilized atomically precise, superatomic silver nanoparticles for catalysing cycloisomerization of alkynyl amines. *Natl Sci Rev* 2018; **5**: 694–702.
23. Liu J-Y, Alkan F, Wang Z *et al.* Different silver nanoparticles in one crystal: $Ag_{210}(^{i}PrPhS)_{71}(Ph_3P)_5Cl$ and $Ag_{211}(^{i}PrPhS)_{71}(Ph_3P)_6Cl$. *Angew Chem Int Ed* 2019; **58**: 195–9.
24. Shi C-G, Jia J-H, Jia Y, Li G, Tong M-L. Bulky thiolate-protected silver nanocluster $Ag_{213}(Adm\text{-}S)_{44}Cl_{33}$ with excellent electrocatalytic performance toward oxygen reduction. *CCS Chem* 2023; **5**: 1154–62.
25. Ma M X, Ma X L, Liang G M *et al.* A nanocluster $[Ag_{307}Cl_{62}(SPh^{t}Bu)_{110}]$: chloride intercalation, specific electronic state, and superstability. *J Am Chem Soc* 2021; **143**: 13731–7.
26. Sakthivel NA, Sementa L, Yoon B *et al.* Isomeric thiolate monolayer protected Au_{92} and Au_{102} nanomolecules. *J Phys Chem C* 2019; **124**: 1655–66.
27. Higaki T, Liu C, Zhou M *et al.* Tailoring the structure of 58-electron gold nanoclusters: $Au_{103}S_2(SNap)_{41}$ and its implications. *J Am Chem Soc* 2017; **139**: 9994–10001.

28. Li J, Guan Z, Yuan S, Hu F, Wang Q-M. Enriching structural diversity of alkynyl-protected gold nanoclusters with chlorides. *Angew Chem Int Ed* 2021; **60**: 6699–703.
29. Tang L, Wang L, Wang B, Pei Y, Wang S. Discovering of atomically precise metal nanoclusters by high-throughput syntheses platform. *Chem Eur J* 2024; **30**: e202302602.
30. Guan ZJ, Zeng JL, Yuan SF *et al.* $Au_{57}Ag_{53}(C{\equiv}CPh)_{40}Br_{12}$: a large nanocluster with C_1 symmetry. *Angew Chem Int Ed* 2018; **57**: 5703–7.
31. Zeng JL, Guan ZJ, Du Y *et al.* Chloride-promoted formation of a bimetallic nanocluster $Au_{80}Ag_{30}$ and the total structure determination. *J Am Chem Soc* 2016; **138**: 7848–51.
32. Wang JQ, Shi S, He RL *et al.* Total structure determination of the largest alkynyl-protected fcc gold nanocluster Au_{110} and the study on its ultrafast excited-state dynamics. *J Am Chem Soc* 2020; **142**: 18086–92.
33. Song Y, Li Y, Li H *et al.* Atomically resolved $Au_{52}Cu_{72}(SR)_{55}$ nanoalloy reveals marks decahedron truncation and penrose tiling surface. *Nat Commun* 2020; **11**: 478.
34. Bian G, Chen D, Chen Y *et al.* Remove the innermost atom of a magnetic multi- shell gold nanoparticle for near- unity conversion of CO_2 to CO. *Sci Adv* 2025; **11**: eadu1996.
35. Higaki T, Liu C, Morris DJ *et al.*, $Au_{130-x}Ag_x$ nanoclusters with non-metallicity: a drum of silver-rich sites enclosed in a marks-decahedral cage of gold-Rich sites. *Angew Chem Int Ed* 2019; **58**: 18798–802.
36. Jupally V R, Dass A. Synthesis of $Au_{130}(SR)_{50}$ and $Au_{130-x}Ag_x(SR)_{50}$ nanomolecules through core size conversion of larger metal clusters. *Phys Chem Chem Phys* 2014; **16**: 10473–9.
37. Zeng C, Chen Y, Kirschbaum K *et al.* Structural patterns at all scales in a nonmetallic chiral $Au_{133}(SR)_{52}$ nanoparticle. *Sci Adv* 2015; **1**: e1500045.
38. Dass A, Theivendran S, Nimmala P R *et al.* $Au_{133}(SPh\text{-}t\text{Bu})_{52}$ nanomolecules: X-ray crystallography, optical, electrochemical, and theoretical analysis. *J Am Chem Soc* 2015; **137**: 4610–3.
39. Liu L-J, Alkan F, Zhuang S *et al.* Atomically precise gold nanoclusters at the molecular-to-metallic transition with intrinsic chirality from surface layers. *Nat Commun* 2023; **14**: 2397.
40. Kumara C, Dass A. $(AuAg)_{144}(SR)_{60}$ alloy nanomolecules. *Nanoscale* 2011; **3**: 3064–7.
41. Yan N, Xia N, Liao L *et al.* Unraveling the long-pursued Au_{144} structure by X-ray crystallography. *Sci Adv* 2018; **4**: eaat7259.
42. Lei Z, Li J, Wan X, Zhang W, Wang Q-M. Isolation and total structure determination of an all-alkynyl-protected gold nanocluster Au_{144}. *Angew Chem Int Ed* 2018; **57**: 8639–43.

43. Vergara S, Lukes DA, Martynowycz MW *et al.* MicroED structure of Au_{146}(p-MBA)$_{57}$ at subatomic resolution reveals a twinned fcc cluster. *J Phys Chem Lett* 2017; **8**, 5523–30.
44. Hu F, Guan ZJ, Yang G *et al.* Molecular gold nanocluster Au_{156} showing metallic electron dynamics. *J Am Chem Soc* 2021; **143**: 17059–67.
45. Negishi Y, Sakamoto C, Ohyama T, Tsukuda T. Synthesis and the origin of the stability of thiolate-protected Au_{130} and Au_{187} clusters. *J Phys Chem Lett* 2012; **3**: 1624–8.
46. Fang Y, Wang P, Pei Y, Xiong L. Architectural blueprint of fcc gold nanoclusters: modular assembly of $Au_{188}(SR)_{60}$ and $Au_{110}(SR)_{48}$ from tetrahedral Au_4 and octahedral Au_6 geometric modules. *J Phys Chem Lett* 2025; **16**: 4675–82.
47. Sakthivel NA, Shabaninezhad M, Sementa L *et al.* The missing link: Au_{191}(SPh-tBu)$_{66}$ Janus nanoparticle with molecular and bulk-metal-like properties. *J Am Chem Soc* 2020; **142**: 15799–814.
48. Zhou M, Zeng C, Song Y *et al.* On the non-metallicity of 2.2 nm $Au_{246}(SR)_{80}$ nanoclusters. *Angew Chem Int Ed* 2017; **56**, 16257–61.
49. Yan J, Malola S, Hu C *et al.* Co-crystallization of atomically precise metal nanoparticles driven by magic atomic and electronic shells. *Nat Commun* 2018; **9**: 3357.
50. Sakthivel NA, Theivendran S, Ganeshraj V, Oliver AG, Dass A. Crystal structure of Faradaurate-279: Au_{279}(SPh-tBu)$_{84}$ plasmonic nanocrystal molecules. *J Am Chem Soc* 2017; **139**, 15450–9.
51. Kumara C, Dass A. $Au_{329}(SR)_{84}$ nanomolecules: compositional assignment of the 76.3 kDa plasmonic faradaurates. *Anal Chem* 2014; **86**: 4227–32.
52. Zhou M, Zeng C, Chen Y *et al.* Evolution from the plasmon to exciton state in ligand-protected atomically precise gold nanoparticles. *Nat Commun* 2016; **7**: 13240.
53. Kumara C, Zuo X, Ilavsky J *et al.* Super-stable, highly monodisperse plasmonic Faradaurate-500 nanocrystals with 500 gold atoms: $Au_{\sim500}(SR)_{\sim120}$. *J Am Chem Soc* 2014; **136**: 7410–7.
54. Chanaka K, Xiaobing Z, A. CD, Amala D. Faradaurate-940: synthesis, mass spectrometry, electron microscopy, high-energy X-ray diffraction, and X-ray scattering study of $Au_{\sim940\pm20}(SR)_{\sim160\pm4}$ nanocrystals. *ACS Nano* 2014; **8**: 6431–9.
55. Roper D K, Ahn W, Hoepfner. Microscale heat transfer transduced by surface plasmon resonant gold nanoparticles. *J Phys Chem C* 2007; **111**: 3636–41.
